# Supplementary material for: Tetrahydroxanthohumol, a xanthohumol derivative, attenuates high-fat diet-induced hepatic steatosis by antagonizing PPARγ
Source: eLife. 2021 Jun 15;10:e66398. doi: 10.7554/eLife.66398 (PMC8205491; doi:10.7554/eLife.66398)
Supplement: Figure 2—source data 1. — This zip archive contains the following: (1) One Comma Separated Values file named ‘phenome_feeding.csv’ contains food intake and weight entries. (2) One Excel workbook named ‘2019TXN_repeated_measures_YZGB.xlsx’ contains repeated measures analyses. (3) The Jupyter Notebook contains scripts used for statistical analysis and generation of Figure 2. (4) Figure 2—figure supplement 1 folder. A Comma Separated Values file named ‘AUC2.csv’ phenotypic data directly pertaining to Figure 2—figure supplement 1. • A Comma Separated Values file named ‘fast.csv’ phenotypic data directly pertaining to Figure 2—figure supplement 1. A Jupyter Notebook file contains scripts used for statistical analysis and generation of Figure 2—figure supplement 1. A Comma Separated Values file named ‘GTT2.csv’ phenotypic data directly pertaining to Figure 2—figure supplement 1. A pdf file named ‘GTT.pdf’. [file elife-66398-fig2-data1.zip › Figure2/fig2.pdf]

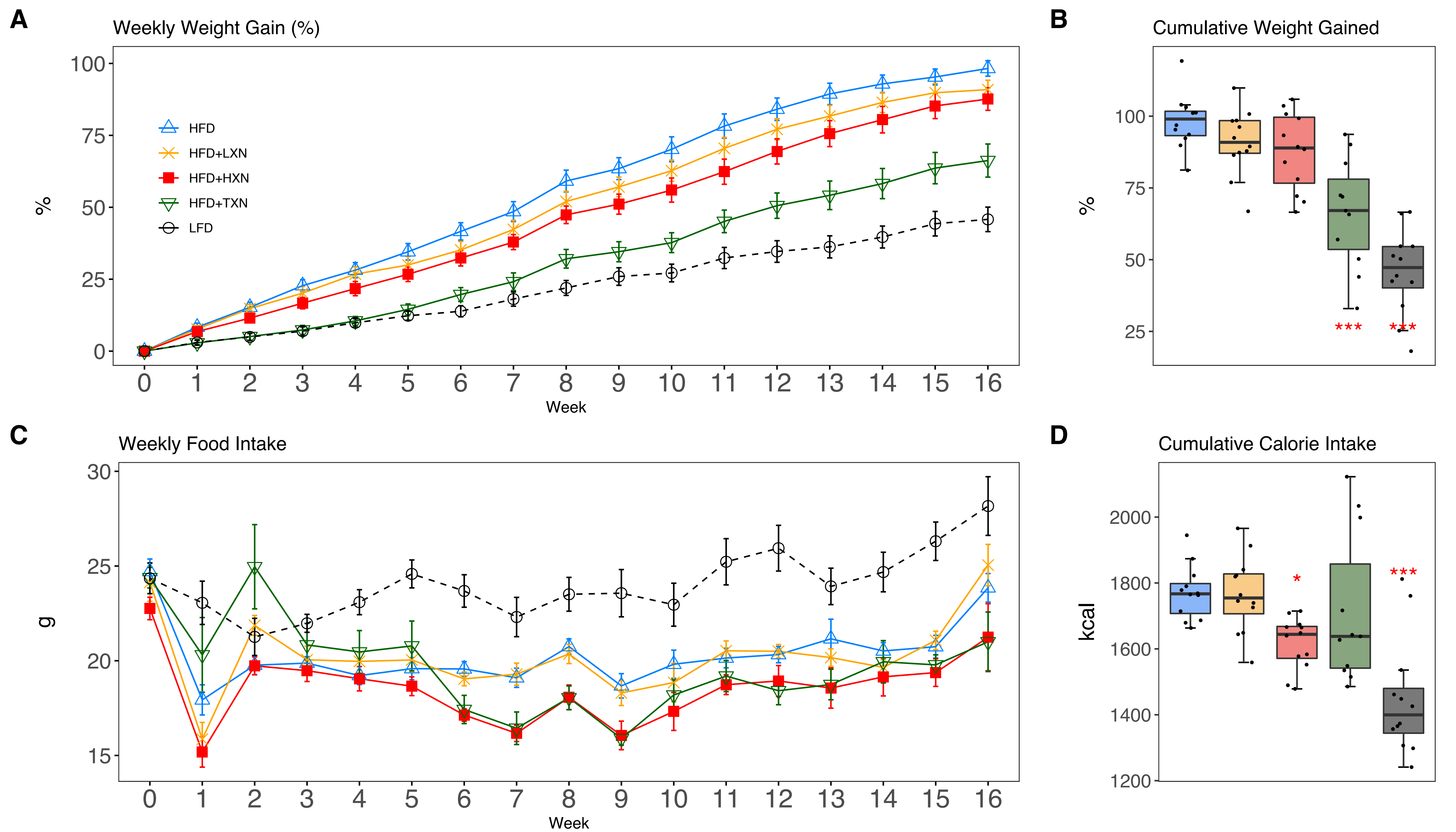
A
Weekly Weight Gain (%)
%
Week
HFD
HFD+LXN
HFD+HXN
HFD+TXN
LFD
B
Cumulative Weight Gained
%
***
***
C
Weekly Food Intake
g
Week
D
Cumulative Calorie Intake
kcal
*
***
