# Supplementary figures and images for: Tetrahydroxanthohumol, a xanthohumol derivative, attenuates high-fat diet-induced hepatic steatosis by antagonizing PPARγ
Source: eLife. 2021 Jun 15;10:e66398. doi: 10.7554/eLife.66398 (PMC8205491; doi:10.7554/eLife.66398)

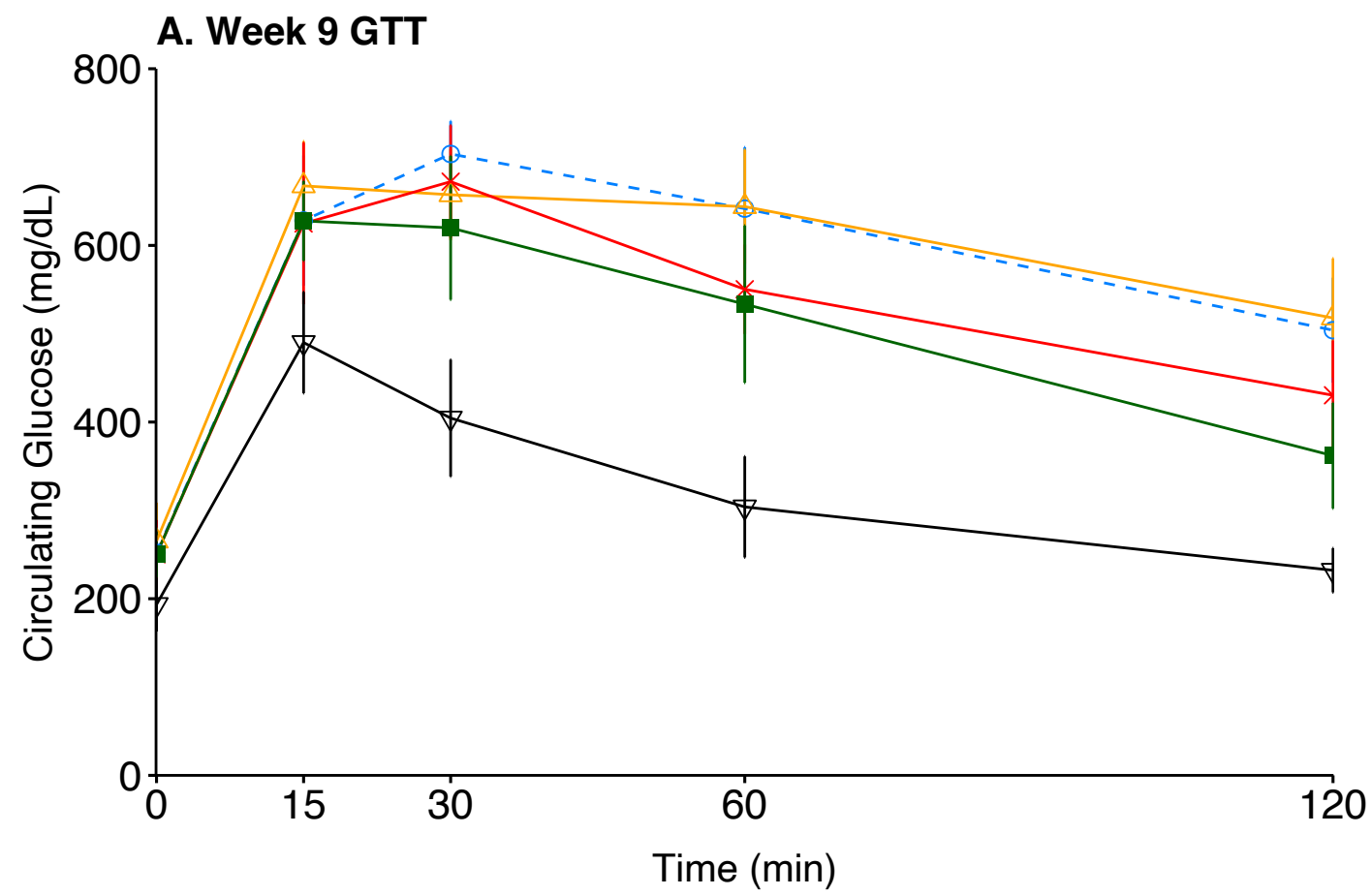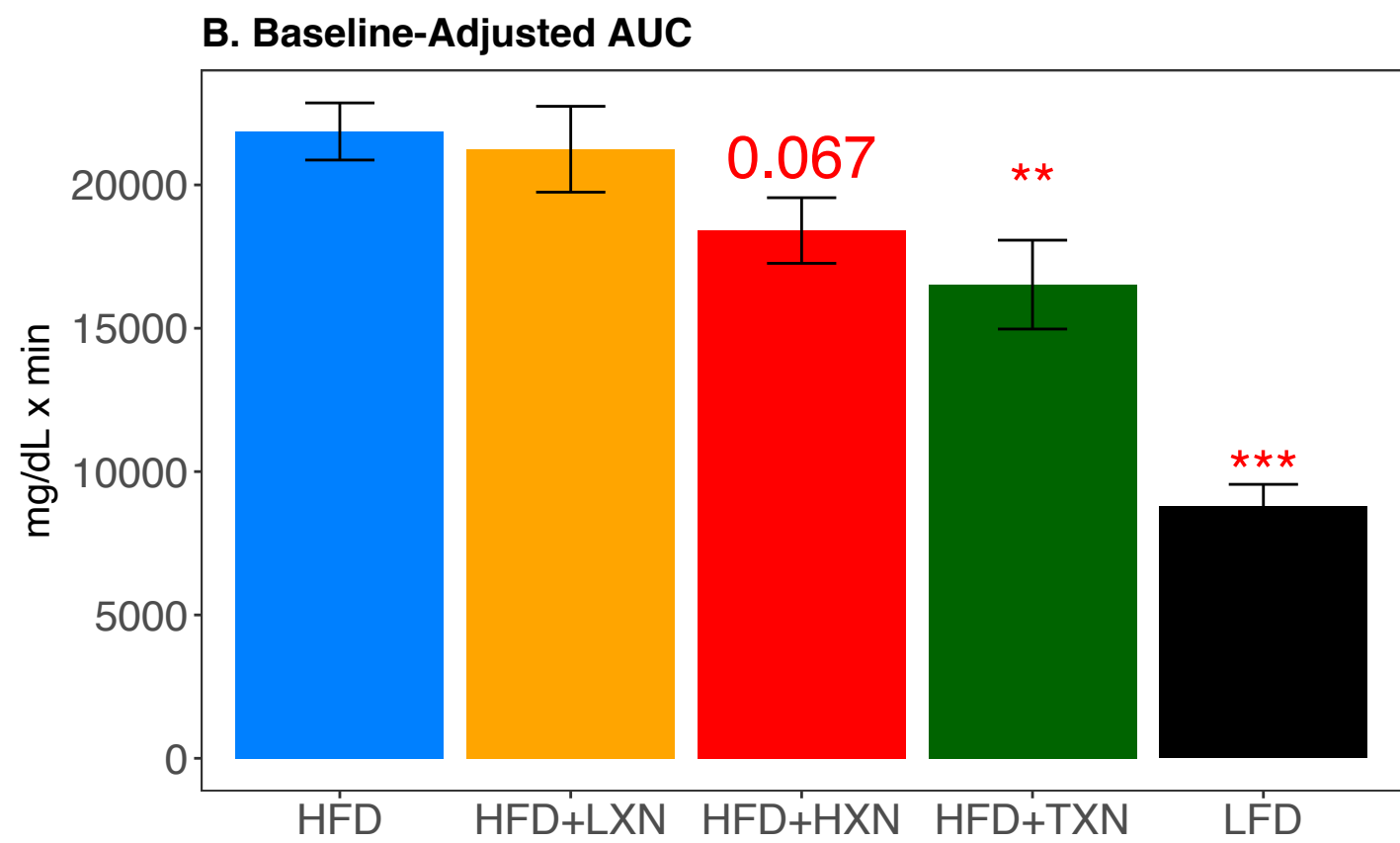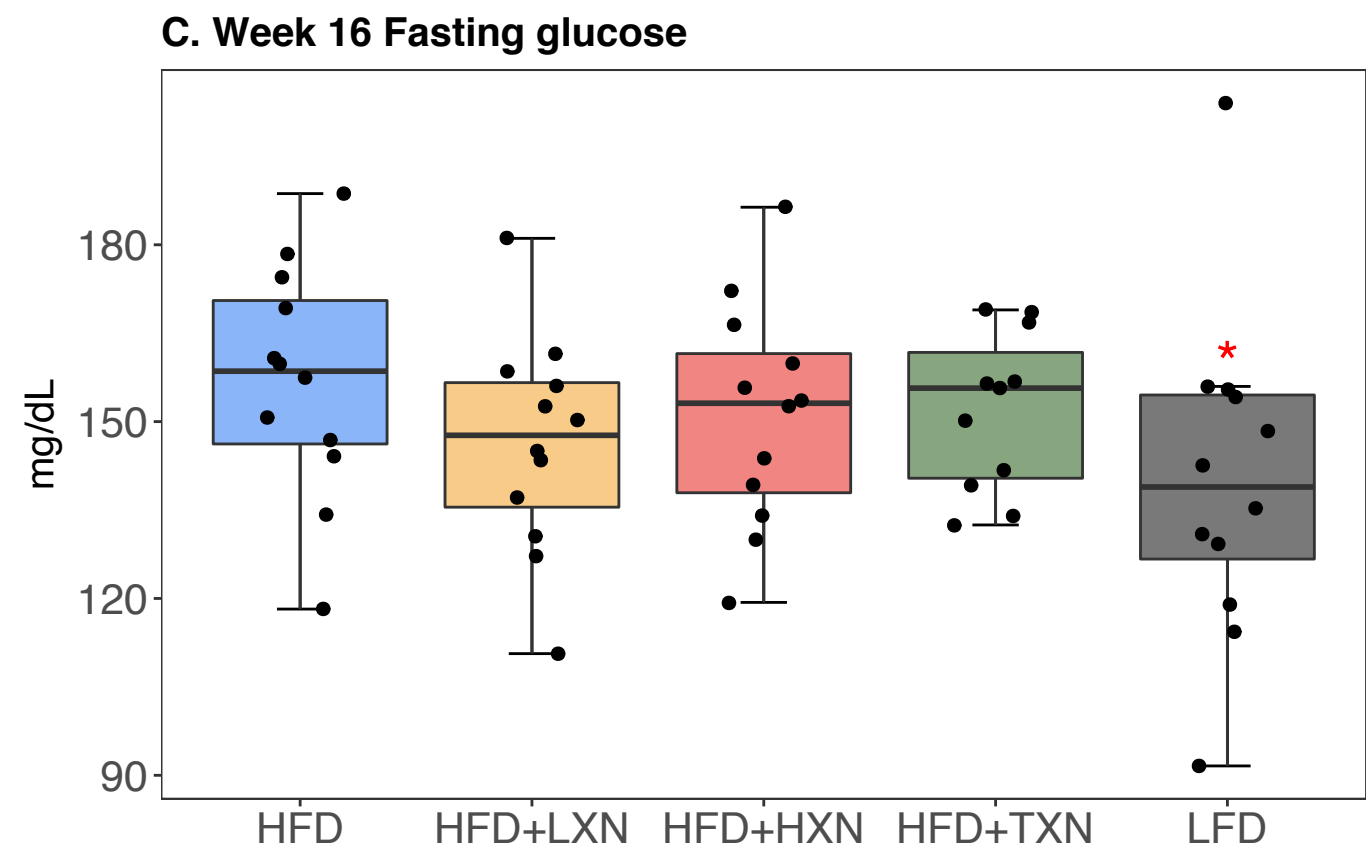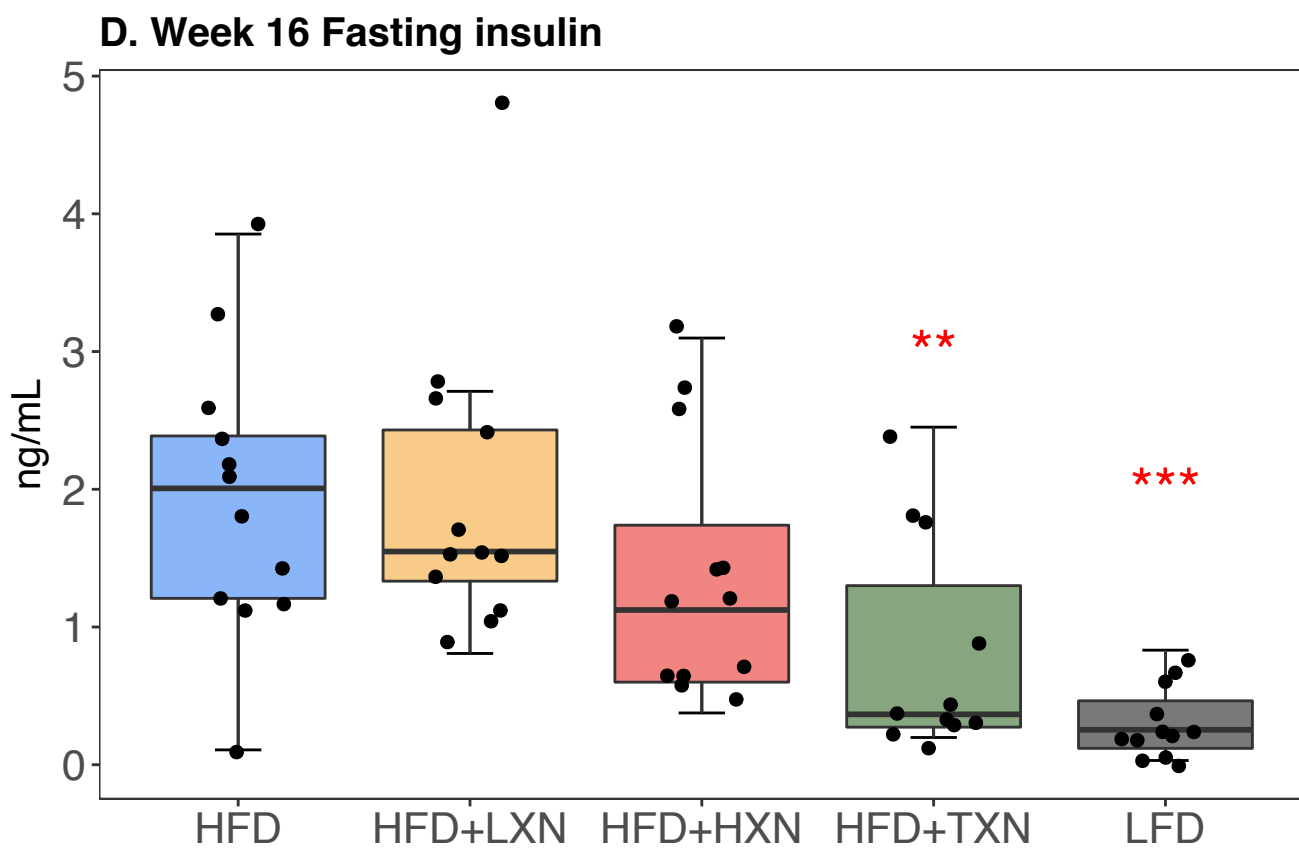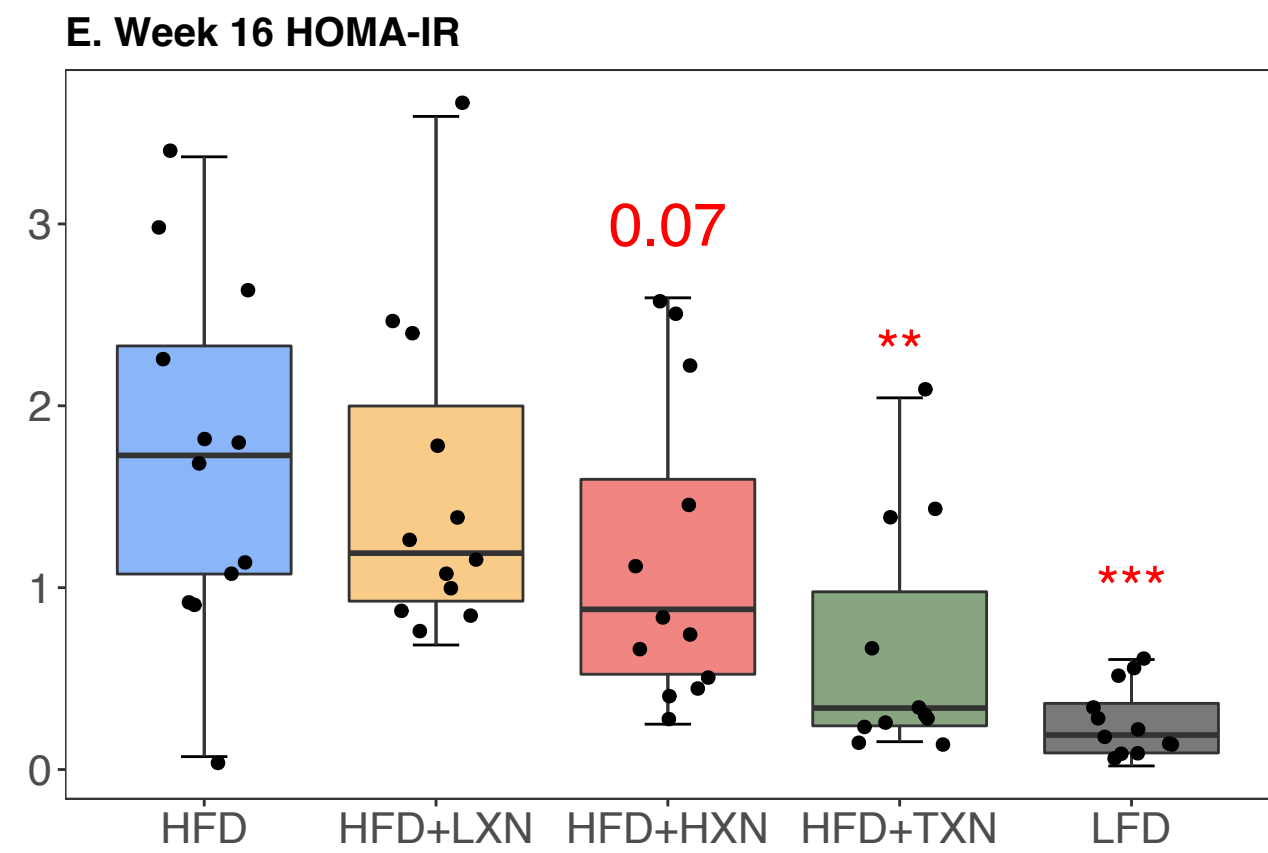

Supplement: Figure 2—source data 1. — This zip archive contains the following: (1) One Comma Separated Values file named ‘phenome_feeding.csv’ contains food intake and weight entries. (2) One Excel workbook named ‘2019TXN_repeated_measures_YZGB.xlsx’ contains repeated measures analyses. (3) The Jupyter Notebook contains scripts used for statistical analysis and generation of Figure 2. (4) Figure 2—figure supplement 1 folder. A Comma Separated Values file named ‘AUC2.csv’ phenotypic data directly pertaining to Figure 2—figure supplement 1. • A Comma Separated Values file named ‘fast.csv’ phenotypic data directly pertaining to Figure 2—figure supplement 1. A Jupyter Notebook file contains scripts used for statistical analysis and generation of Figure 2—figure supplement 1. A Comma Separated Values file named ‘GTT2.csv’ phenotypic data directly pertaining to Figure 2—figure supplement 1. A pdf file named ‘GTT.pdf’. [file elife-66398-fig2-data1.zip › Figure2/Figure 2-supplement 1/GTT.pdf]

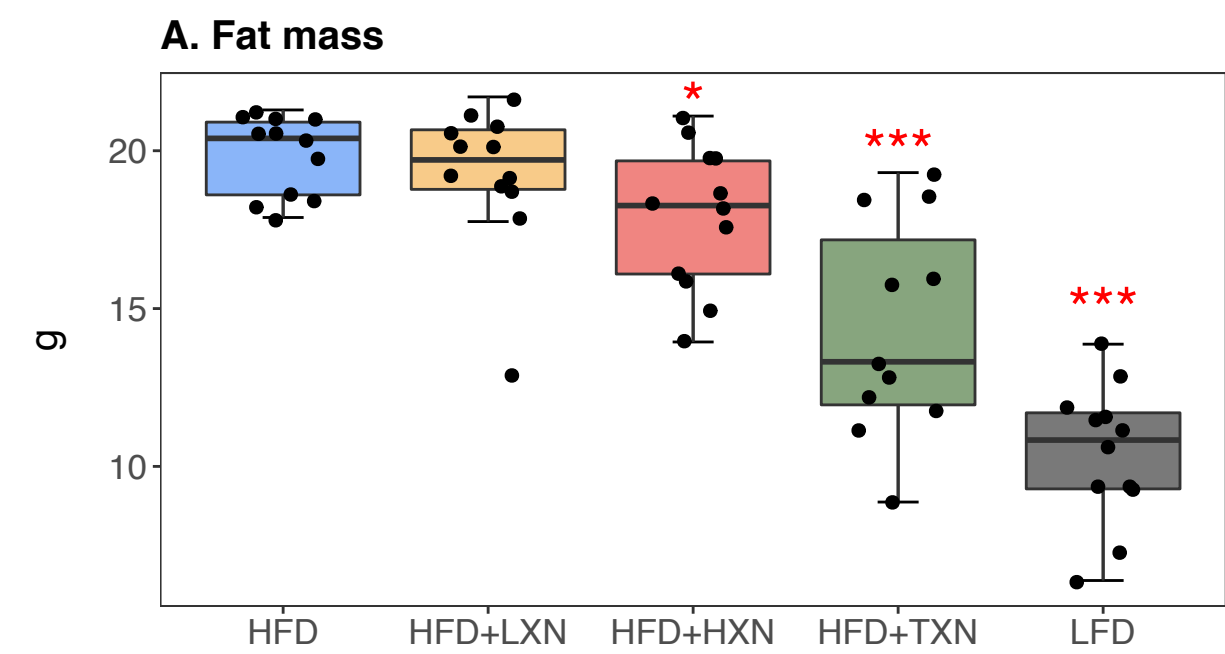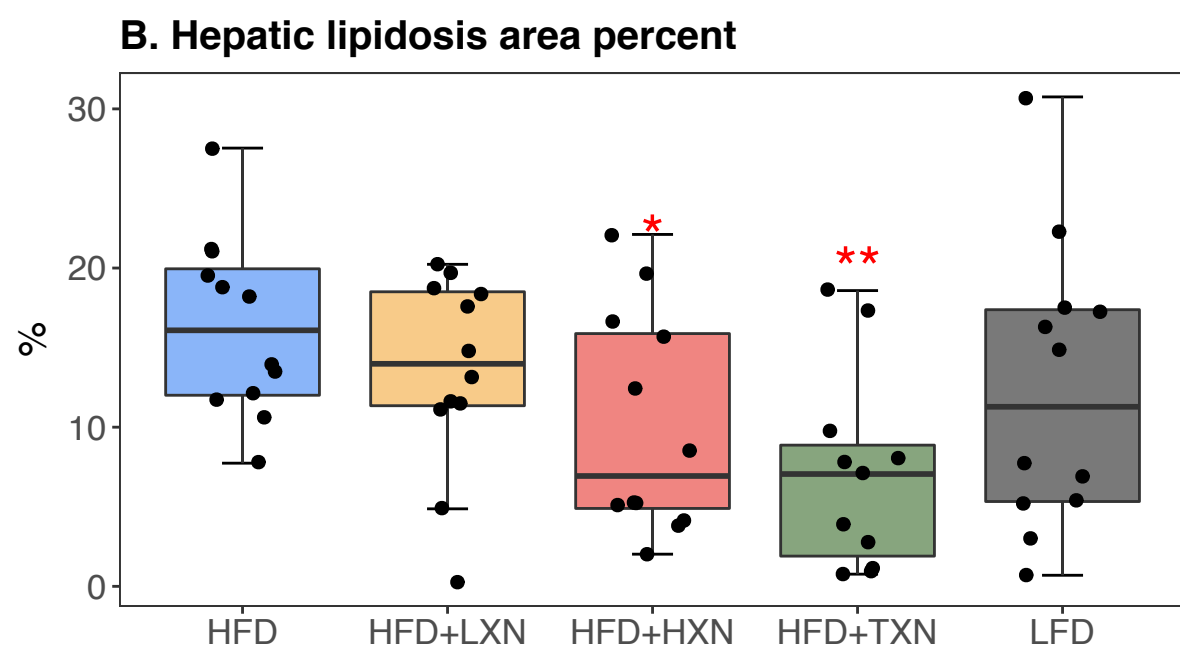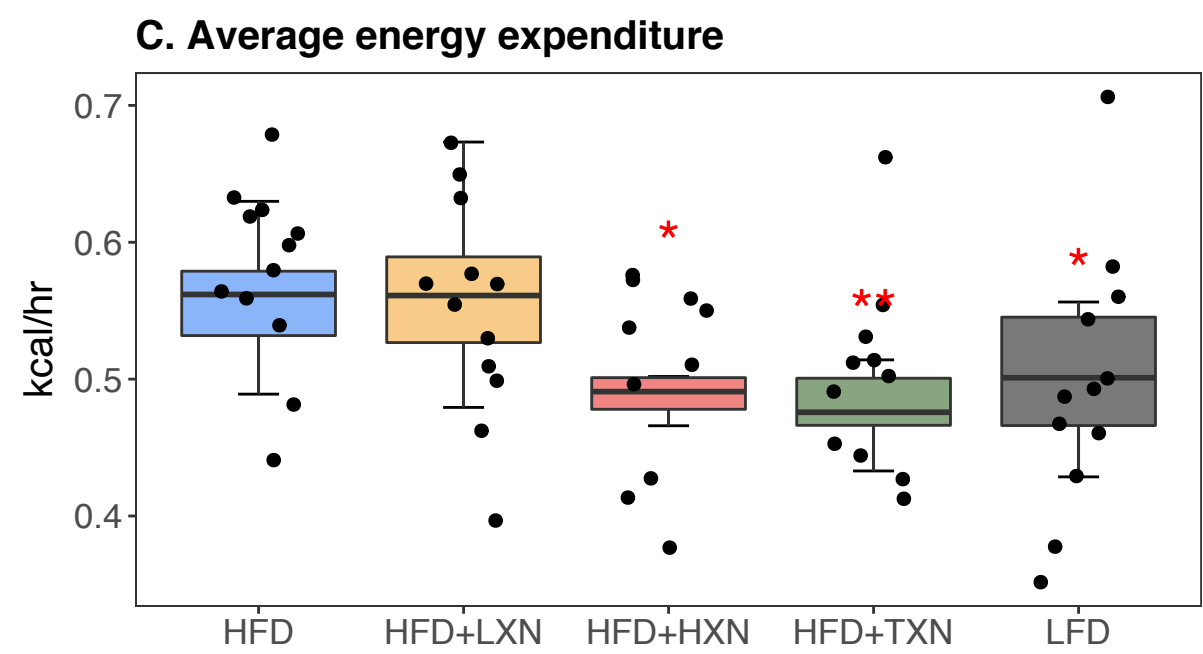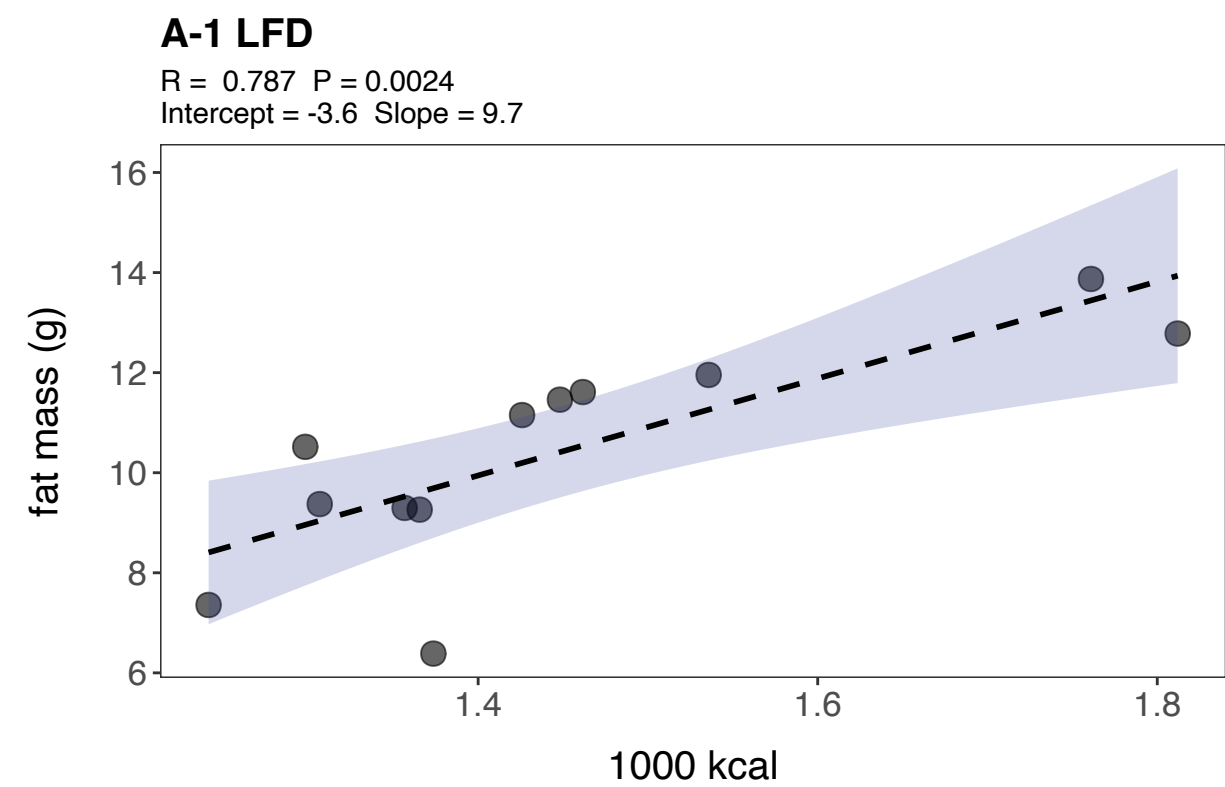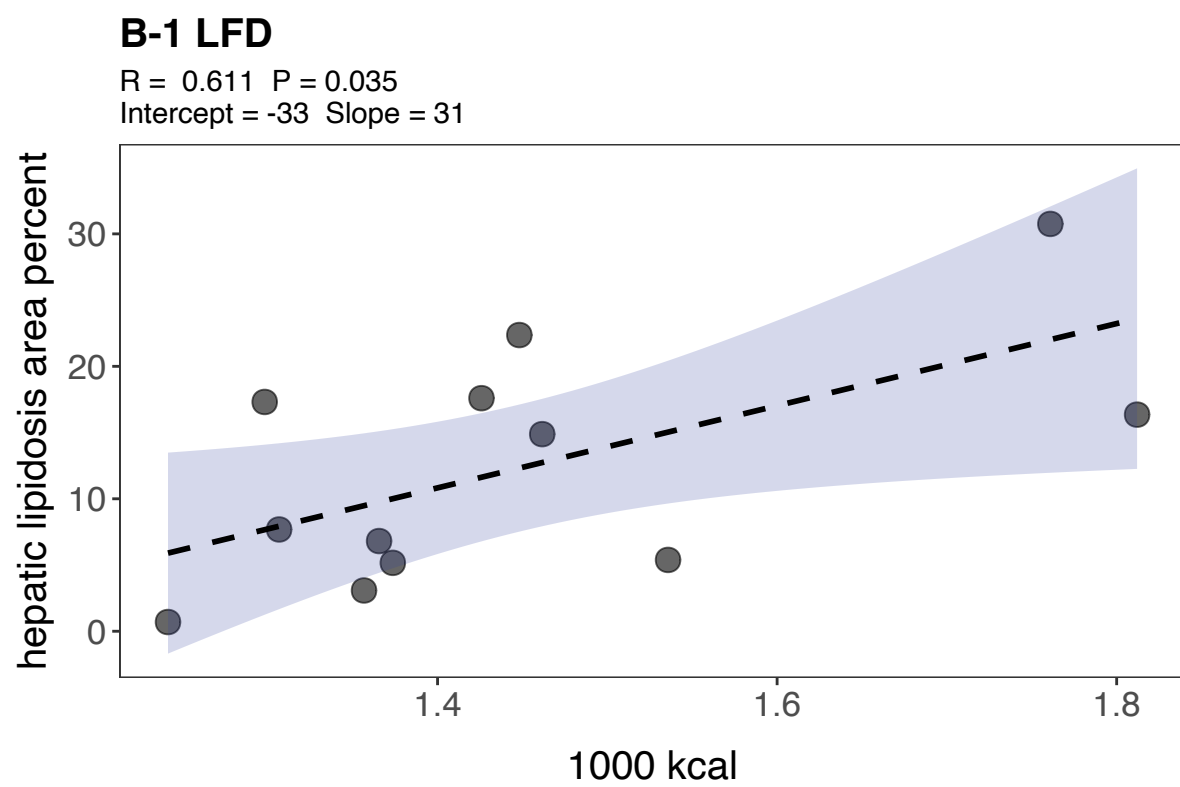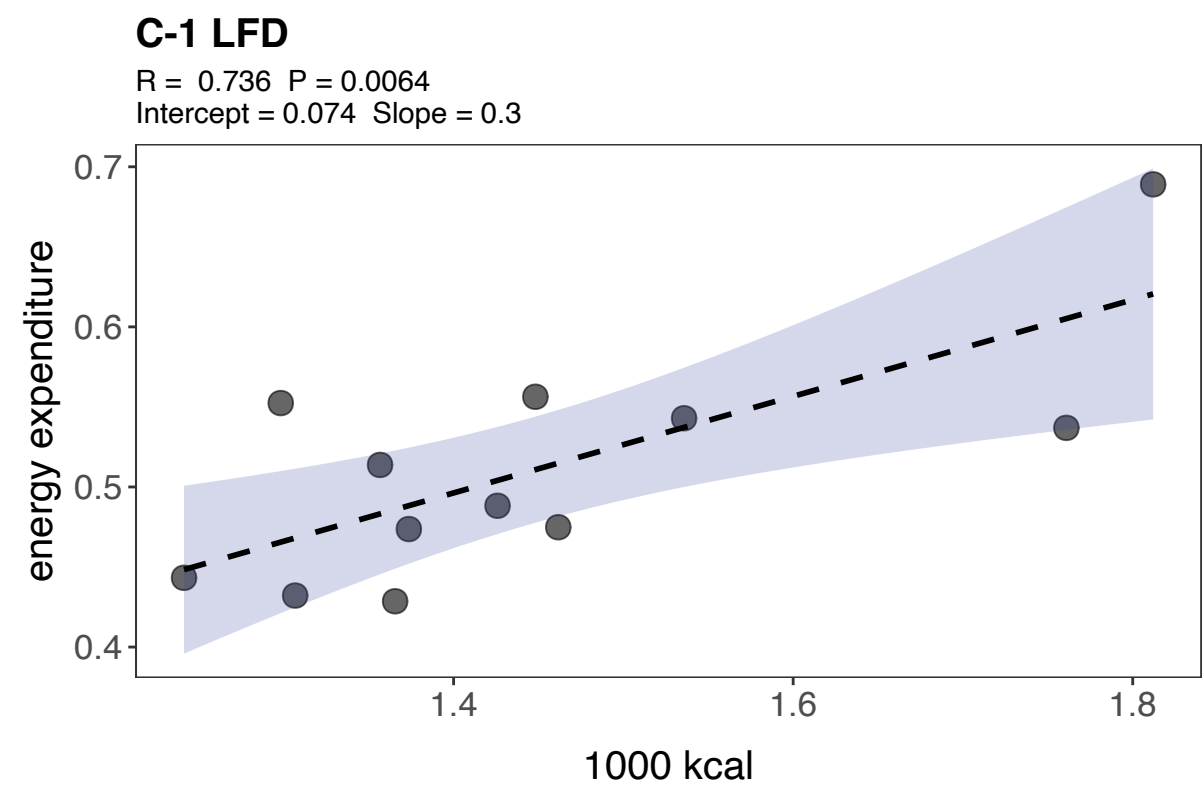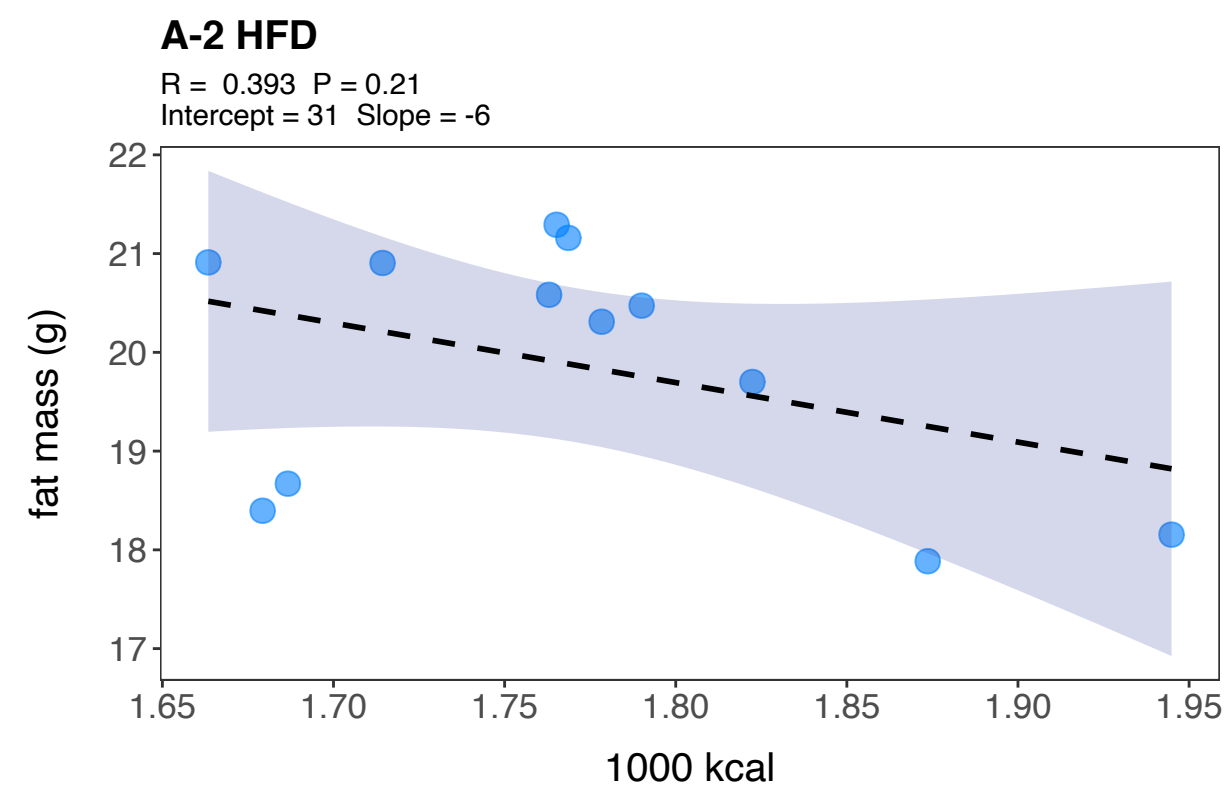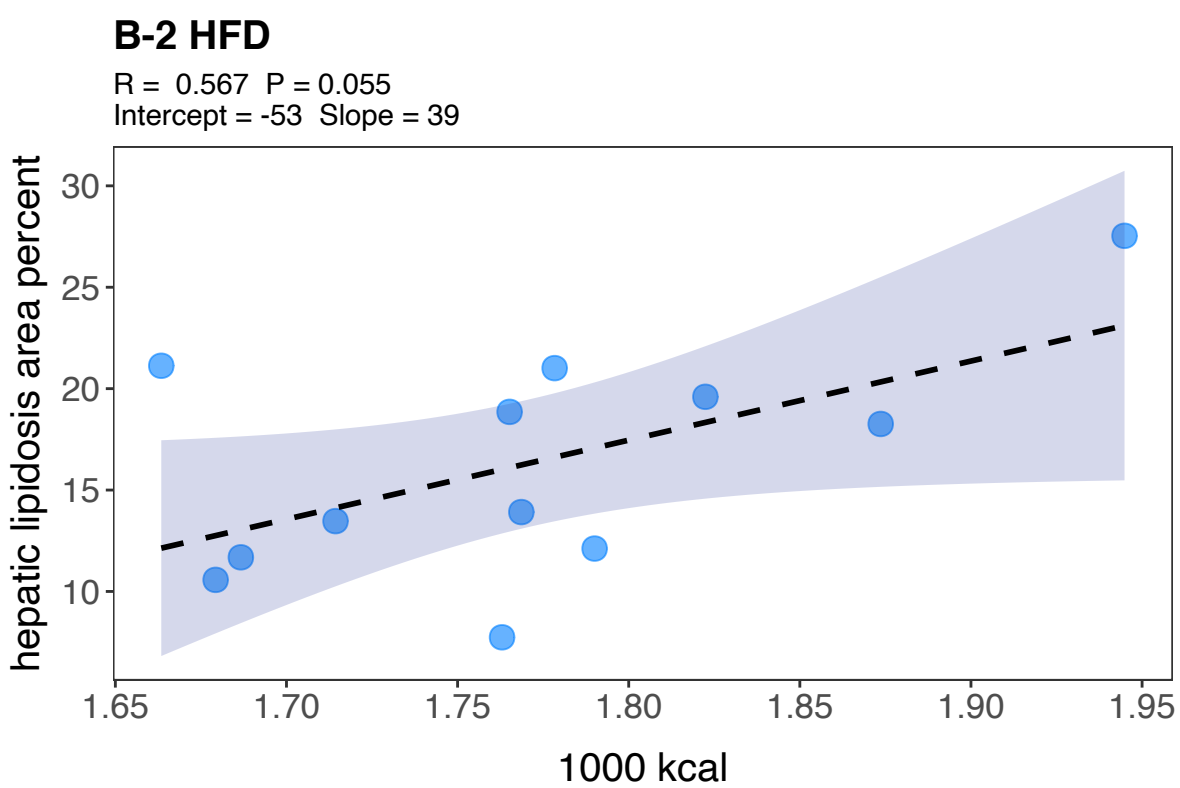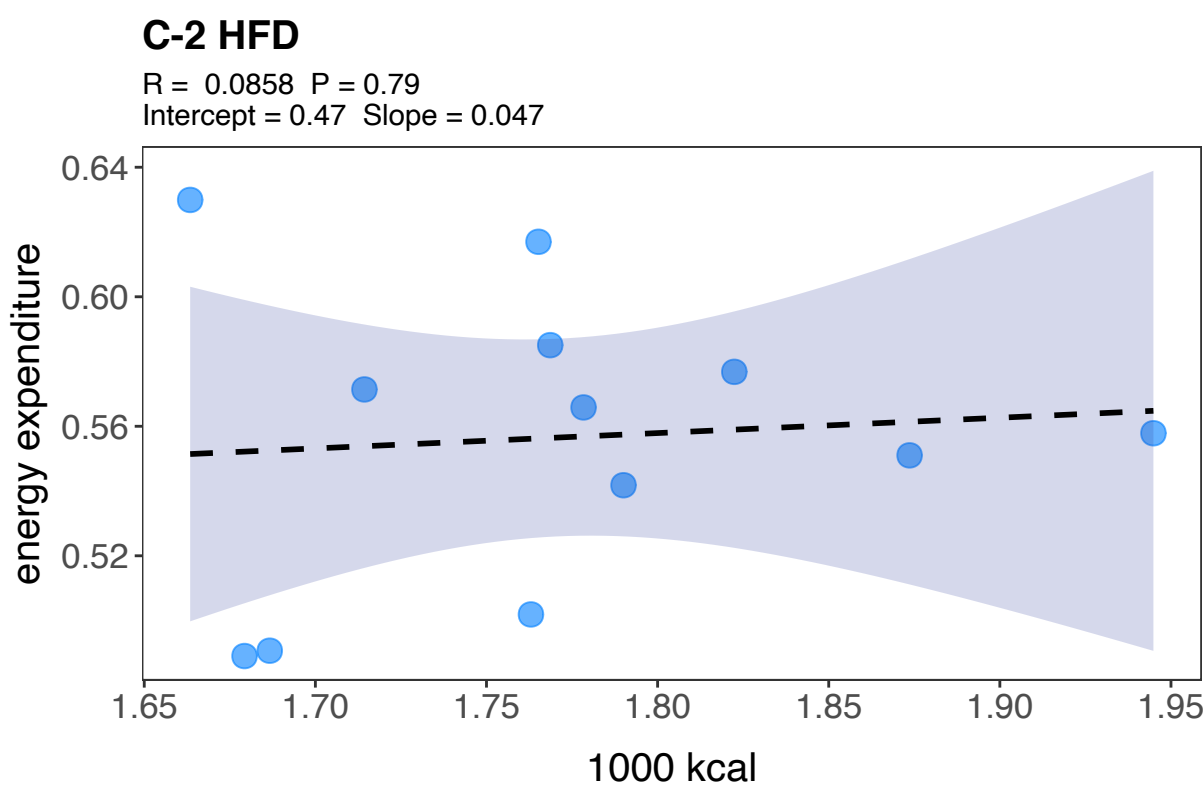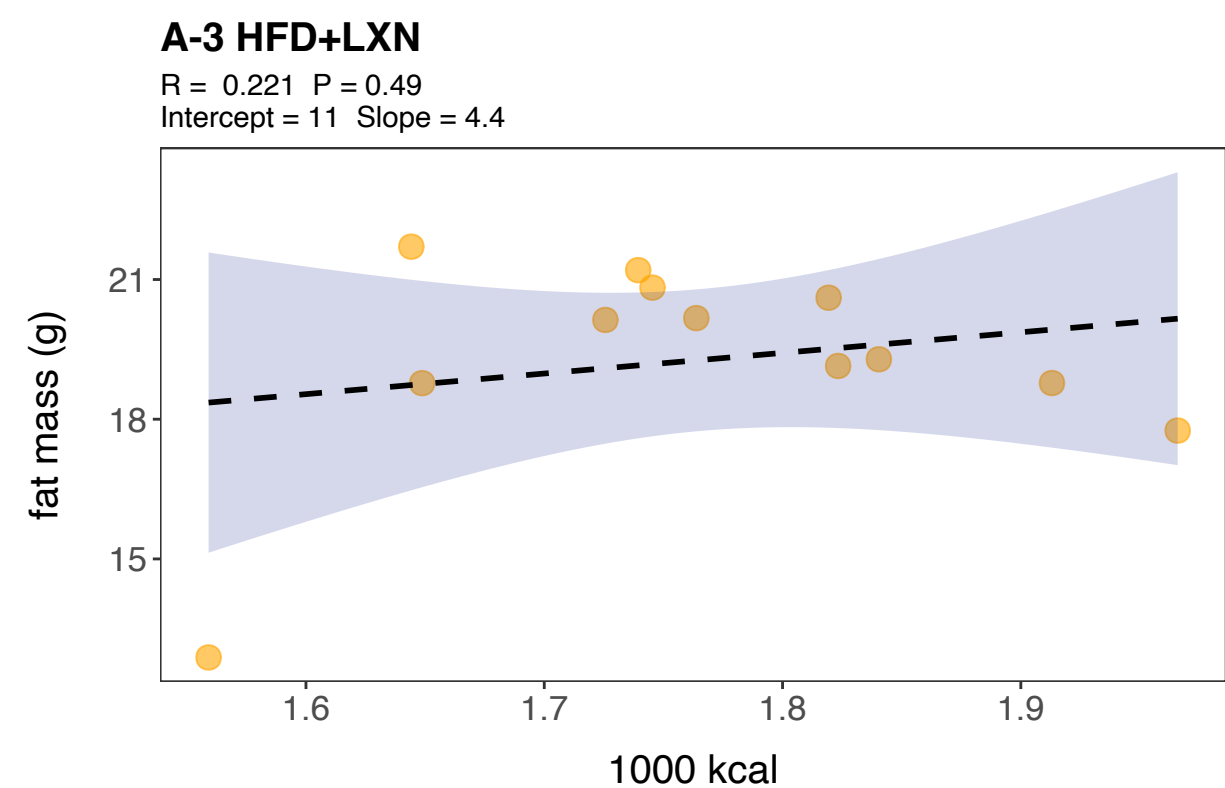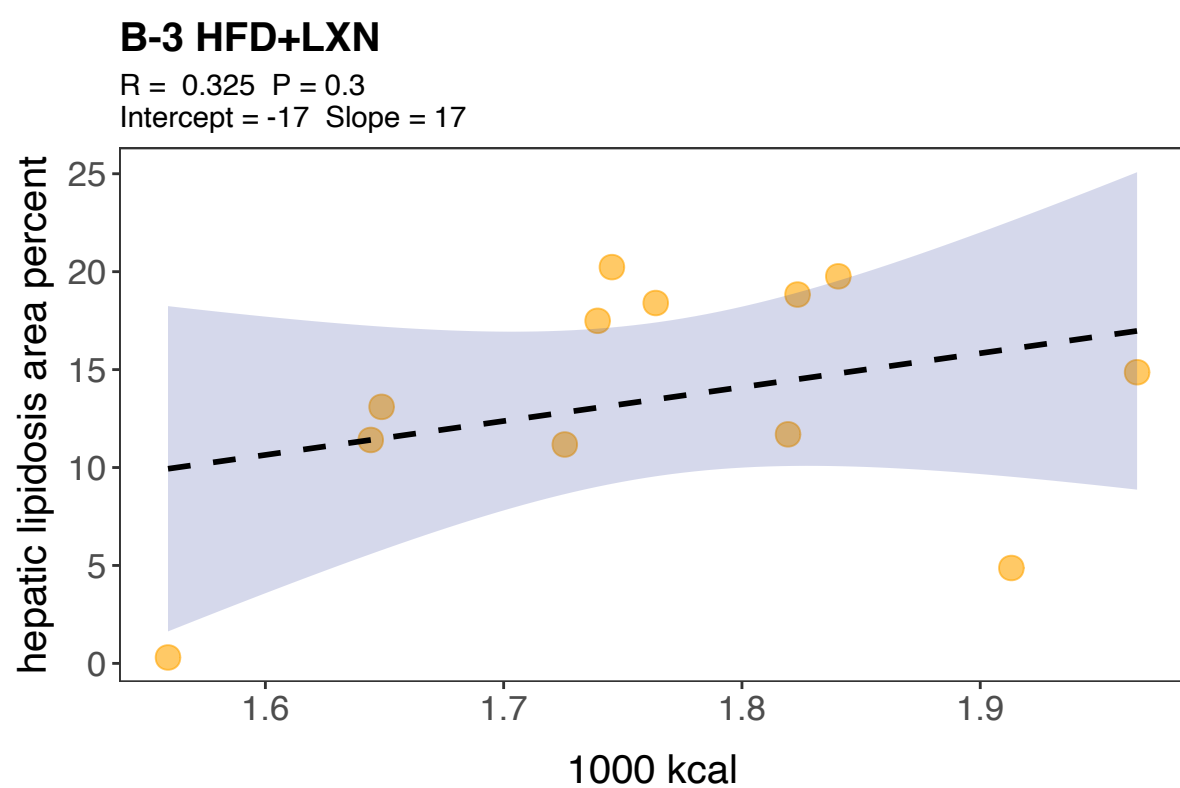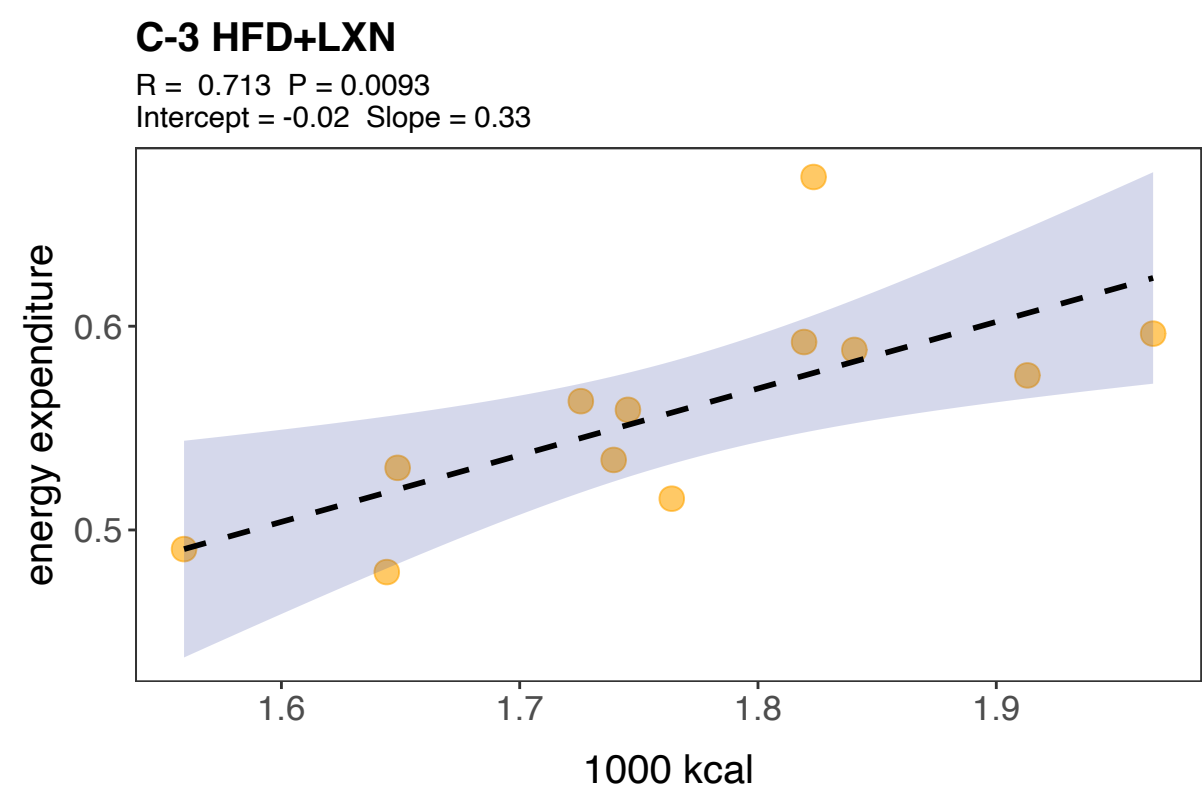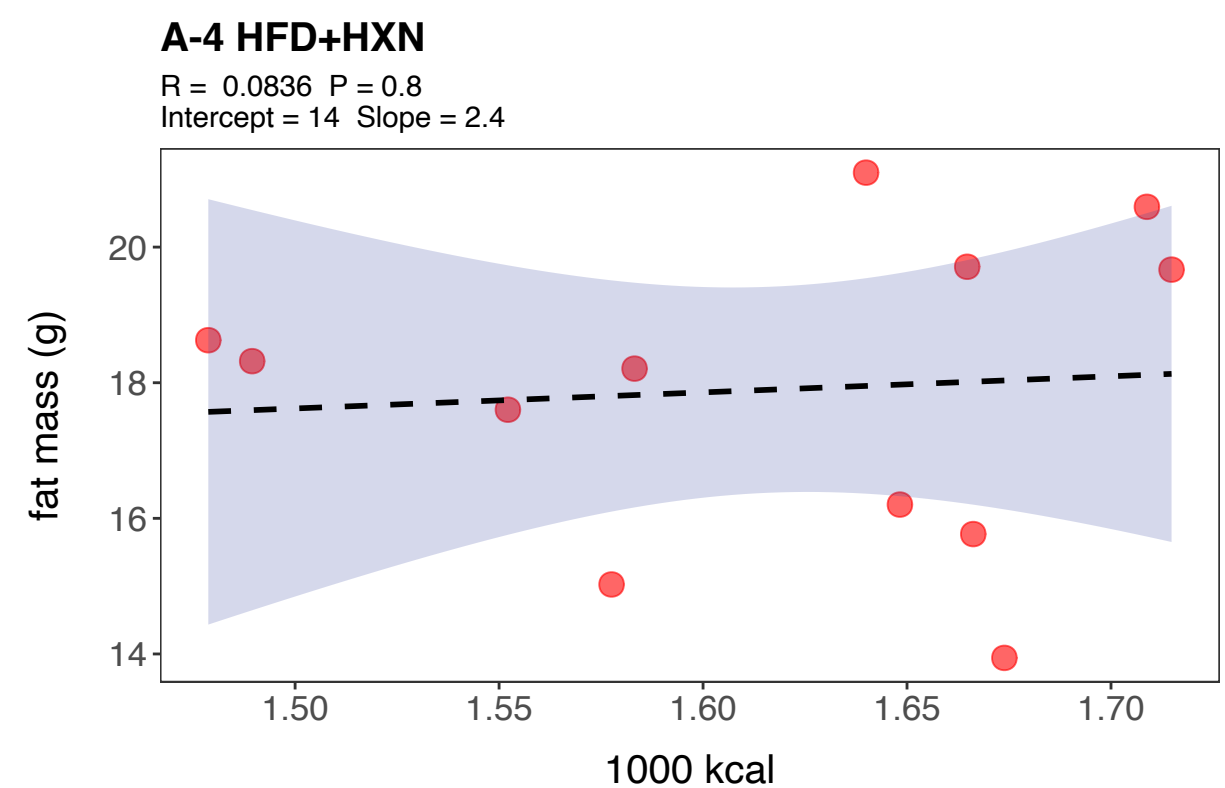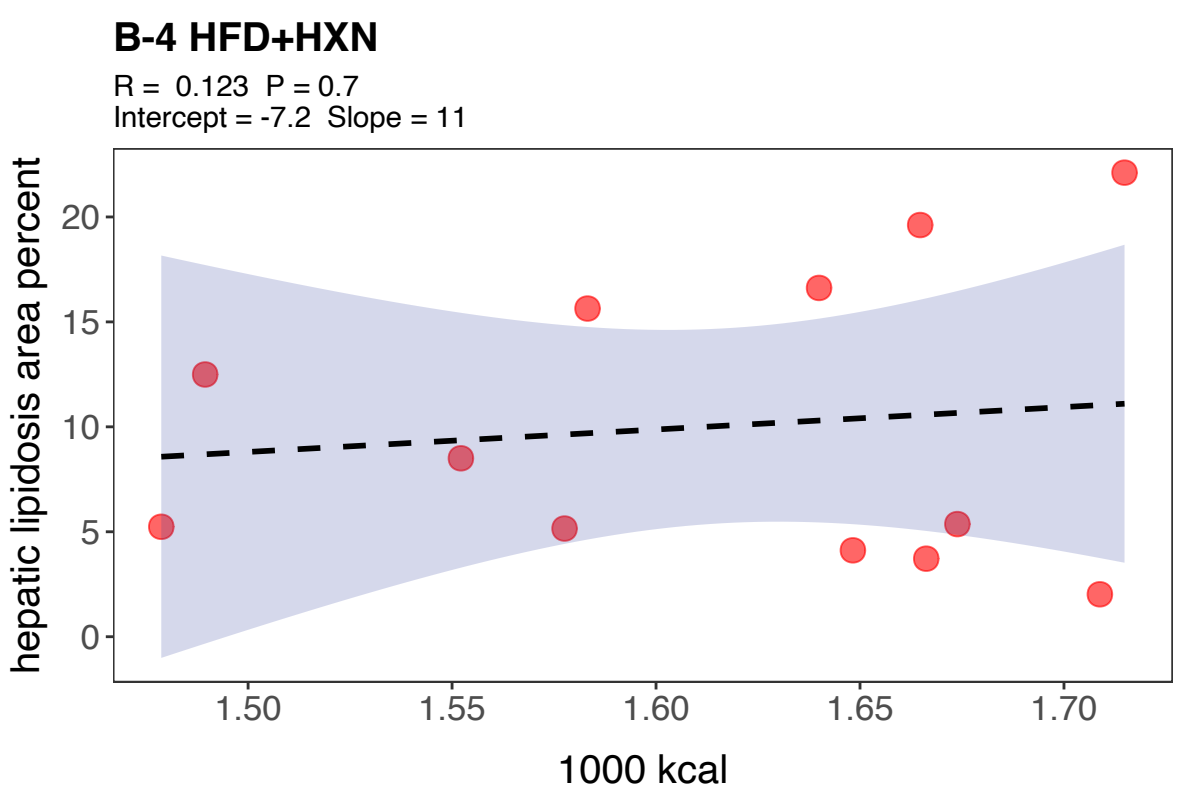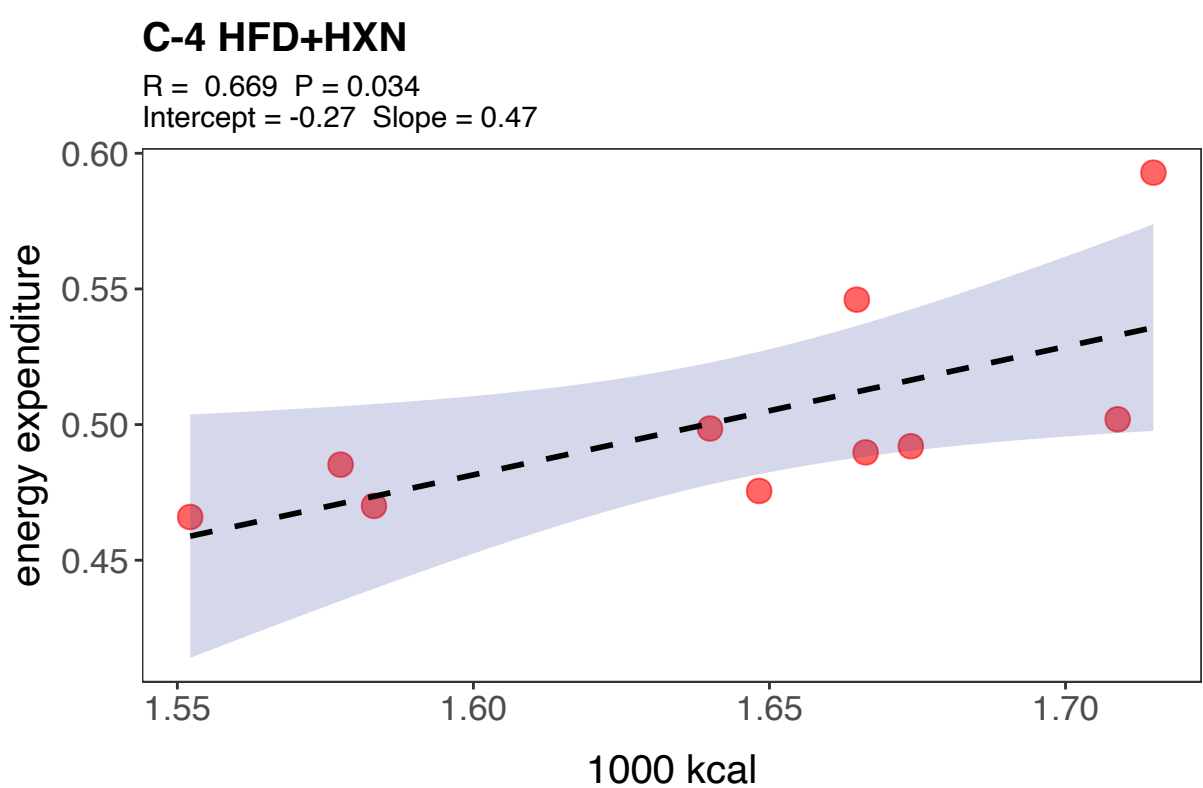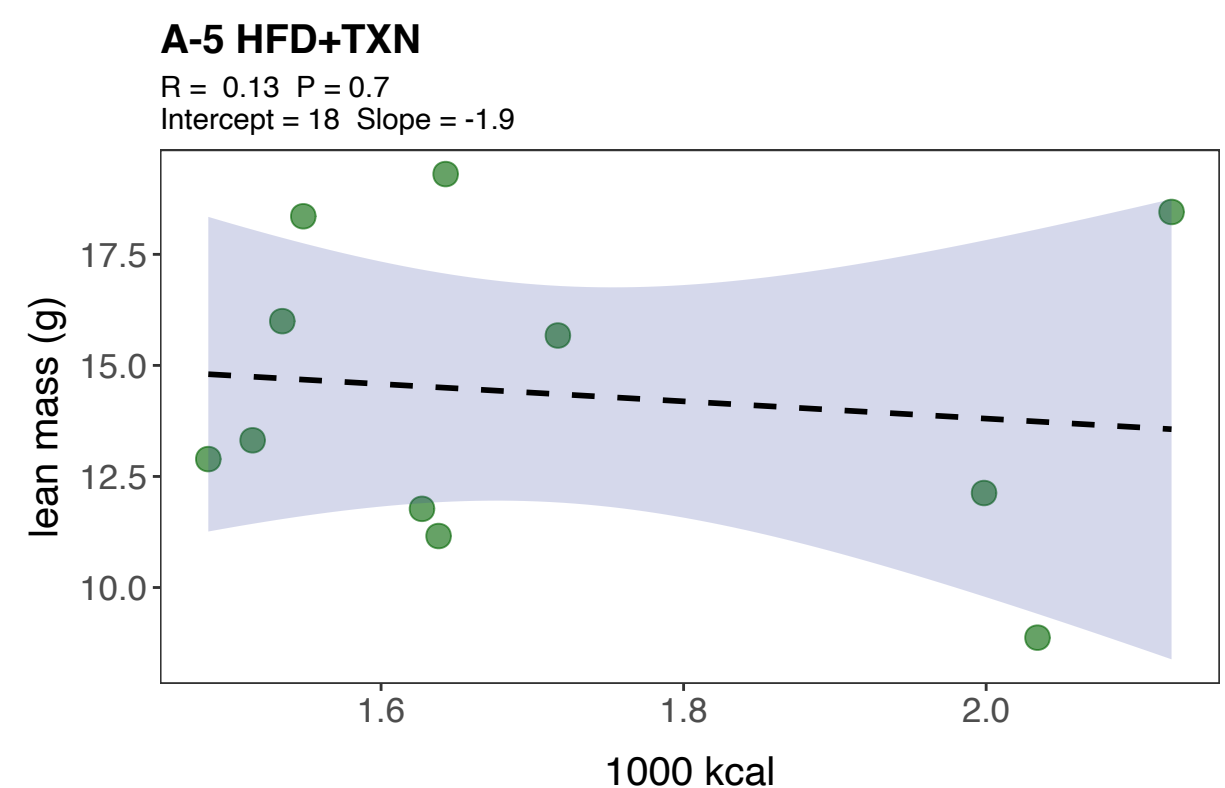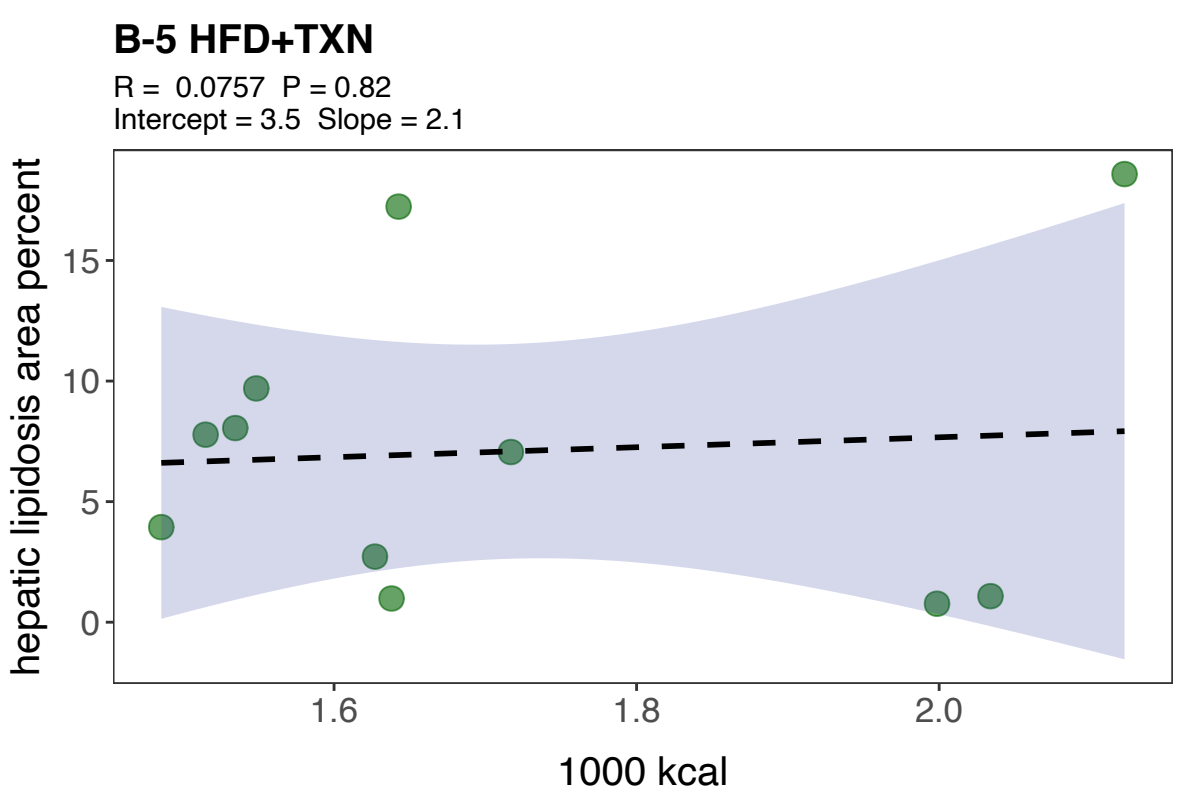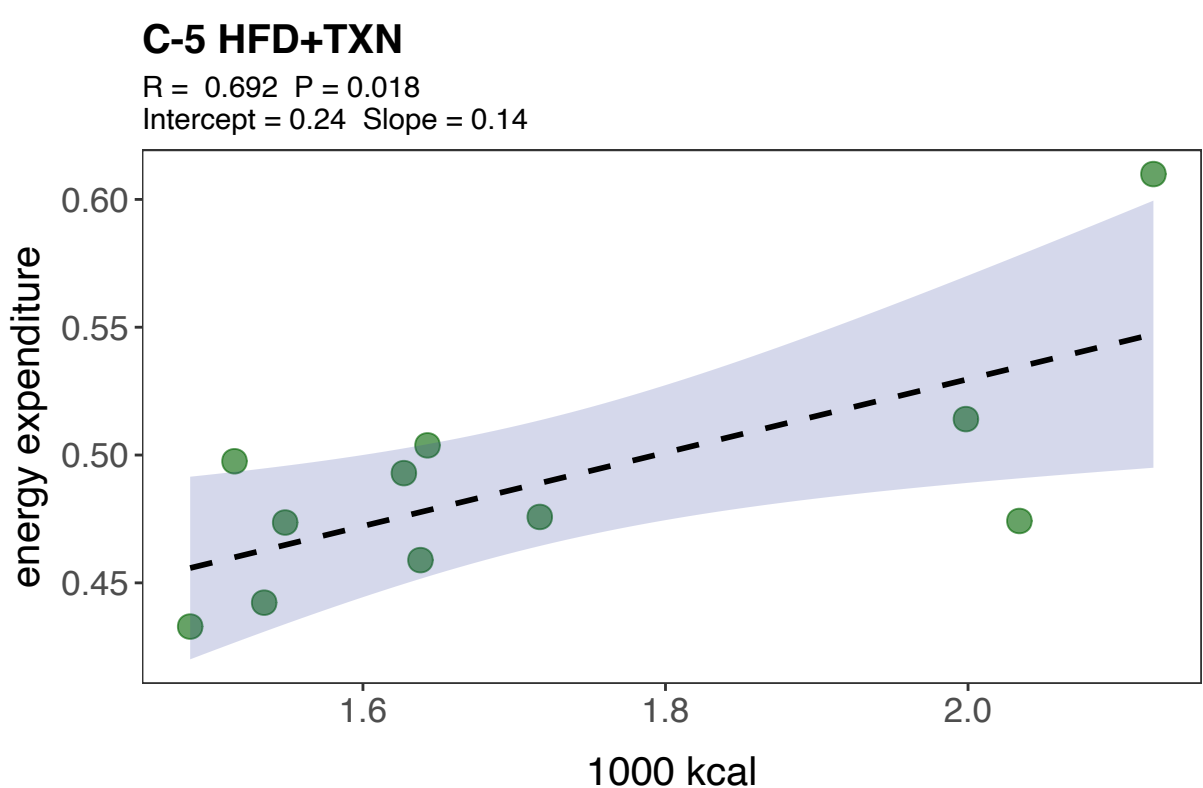

Supplement: Figure 3—source data 1. — This zip archive contains the following: (1) One Comma Separated Values file named ‘metabolicGasExchange.csv’ contains metabolic cage gas exchange data. (2) One Comma Separated Values file named ‘fig3_table.csv’ contains phenotypic data directly pertaining to Figure 3. (3) One Comma Separated Values file named ‘fig3_stat_corrected.csv’ contains corrected metabolic cage gas exchange data directly pertaining to Figure 3. (4) A Jupyter Notebook file contains scripts used for statistical analysis and generation of Figure 3. (5) An R script file ‘ggplotRegression.R’. (6) A folder named ‘Figure 3—figure supplement 1’ containing Figure 3—figure supplement 1. (a) One Comma Separated Values file named ‘metabolicGasExchange.csv’ contains metabolic cage gas exchange data. (b) One Comma Separated Values file named ‘supplement1Table.csv’ contains phenotypic data directly pertaining to Figure 3—figure supplement 1. (c) An R script file “ggplotRegression.R. (d) A Jupyter Notebook file contains scripts used for statistical analysis and generation of Figure 3—figure supplement 1. (7) A folder named ‘Fig3Sup2’ containing Figure 3—figure supplement 2. (a) One Comma Separated Values file named ‘supplement2Table.csv’ contains phenotypic data directly pertaining to Figure 3—figure supplement 2. (b) An R script file ggplotRegression.R. (c) A Jupyter Notebook file contains scripts used for statistical analysis and generation of Figure 3—figure supplement 2. [file elife-66398-fig3-data1.zip › Figure3/Figure3.pdf]

**A. LFD vs. HFD**

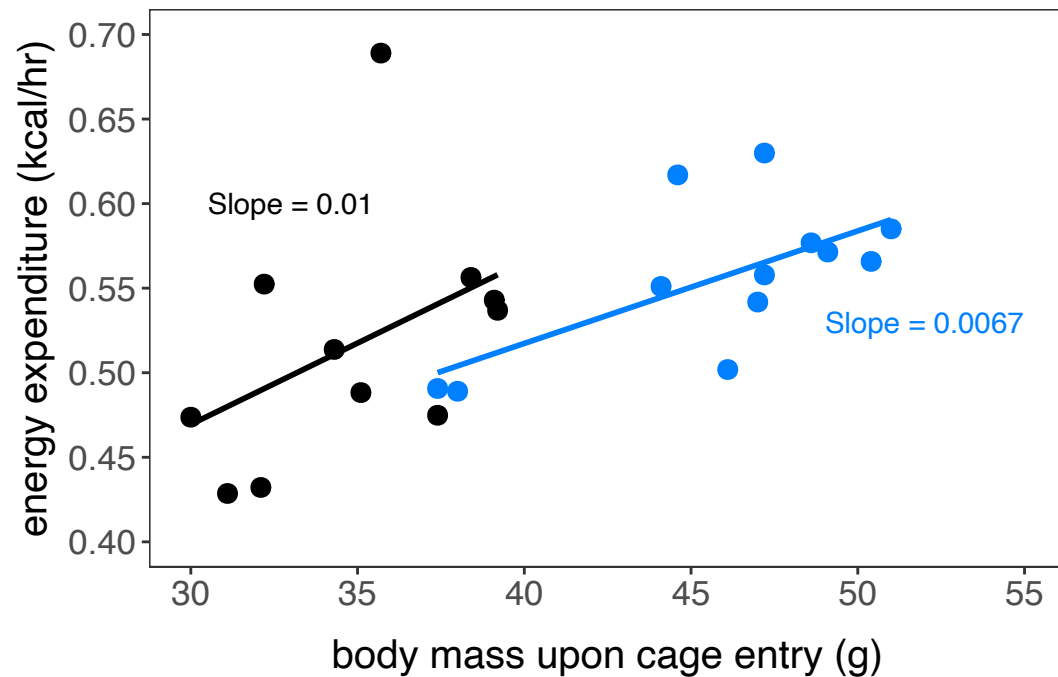

**B. LXXN vs. HFD**

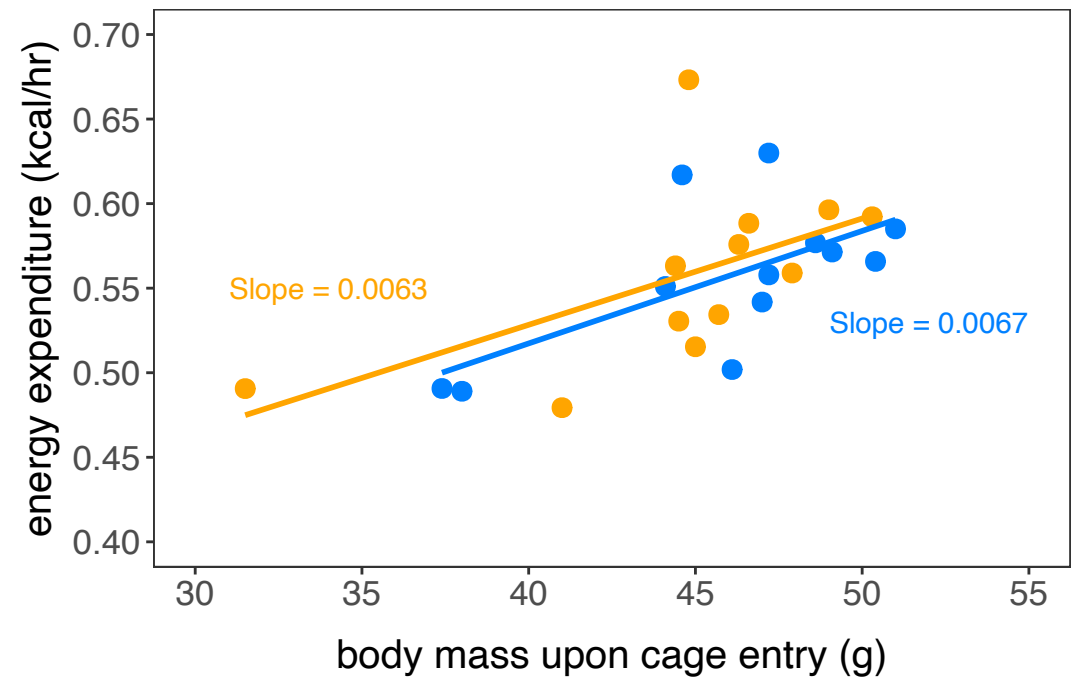

**C. HXXN vs. HFD**

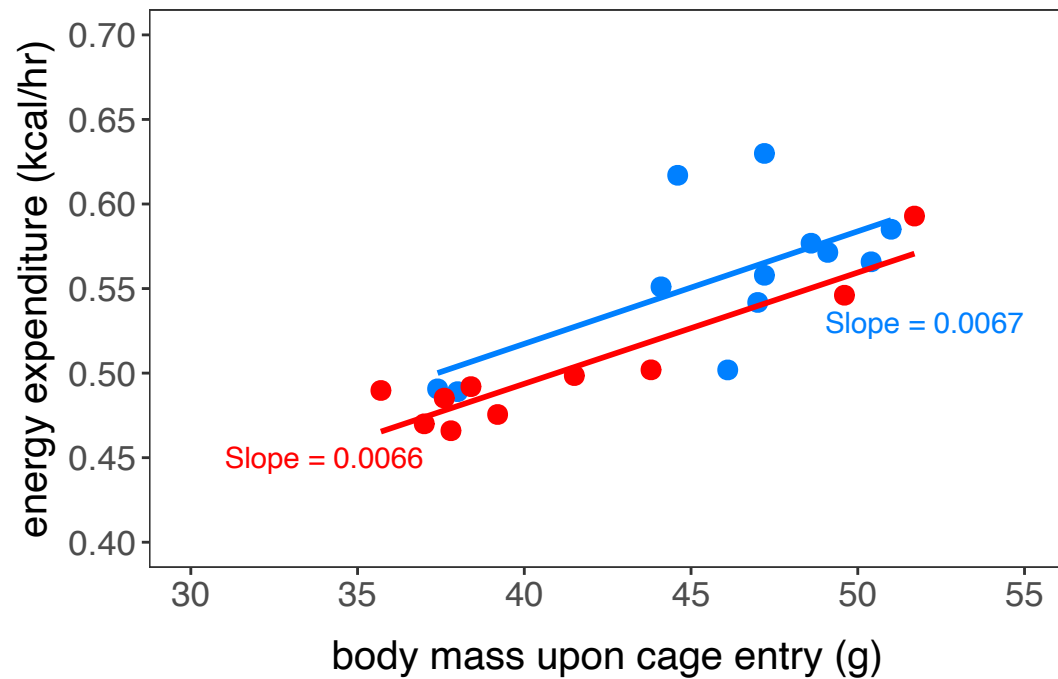

**D. TXN vs. HFD**

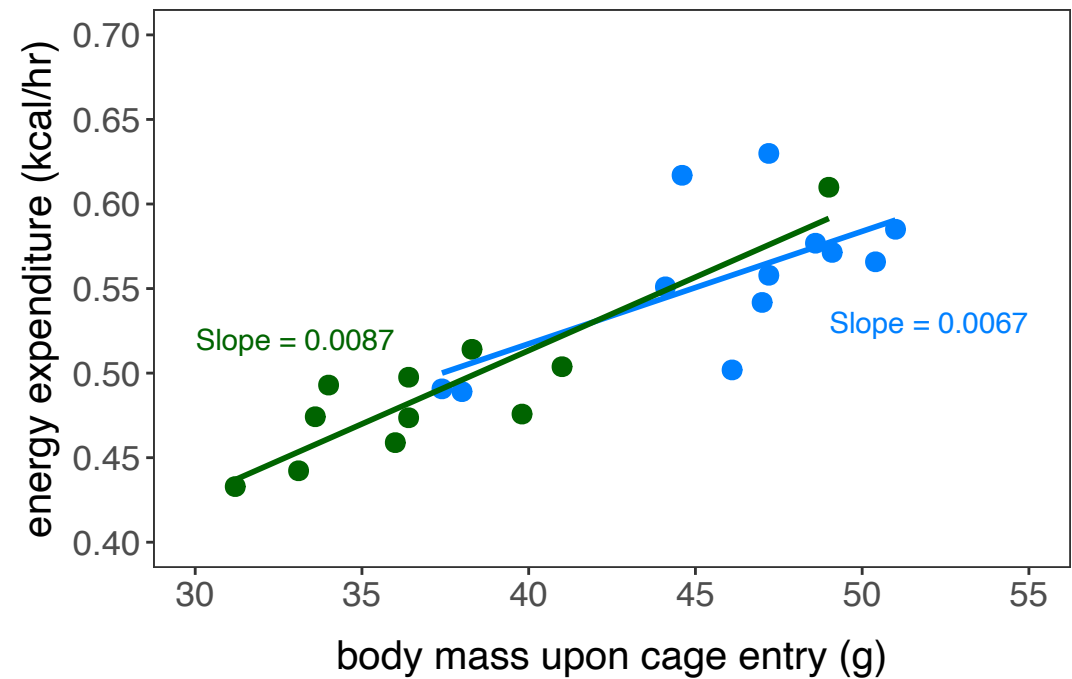

Supplement: Figure 3—source data 1. — This zip archive contains the following: (1) One Comma Separated Values file named ‘metabolicGasExchange.csv’ contains metabolic cage gas exchange data. (2) One Comma Separated Values file named ‘fig3_table.csv’ contains phenotypic data directly pertaining to Figure 3. (3) One Comma Separated Values file named ‘fig3_stat_corrected.csv’ contains corrected metabolic cage gas exchange data directly pertaining to Figure 3. (4) A Jupyter Notebook file contains scripts used for statistical analysis and generation of Figure 3. (5) An R script file ‘ggplotRegression.R’. (6) A folder named ‘Figure 3—figure supplement 1’ containing Figure 3—figure supplement 1. (a) One Comma Separated Values file named ‘metabolicGasExchange.csv’ contains metabolic cage gas exchange data. (b) One Comma Separated Values file named ‘supplement1Table.csv’ contains phenotypic data directly pertaining to Figure 3—figure supplement 1. (c) An R script file “ggplotRegression.R. (d) A Jupyter Notebook file contains scripts used for statistical analysis and generation of Figure 3—figure supplement 1. (7) A folder named ‘Fig3Sup2’ containing Figure 3—figure supplement 2. (a) One Comma Separated Values file named ‘supplement2Table.csv’ contains phenotypic data directly pertaining to Figure 3—figure supplement 2. (b) An R script file ggplotRegression.R. (c) A Jupyter Notebook file contains scripts used for statistical analysis and generation of Figure 3—figure supplement 2. [file elife-66398-fig3-data1.zip › Figure3/Fig3Sup1/fig3Sup1.pdf]

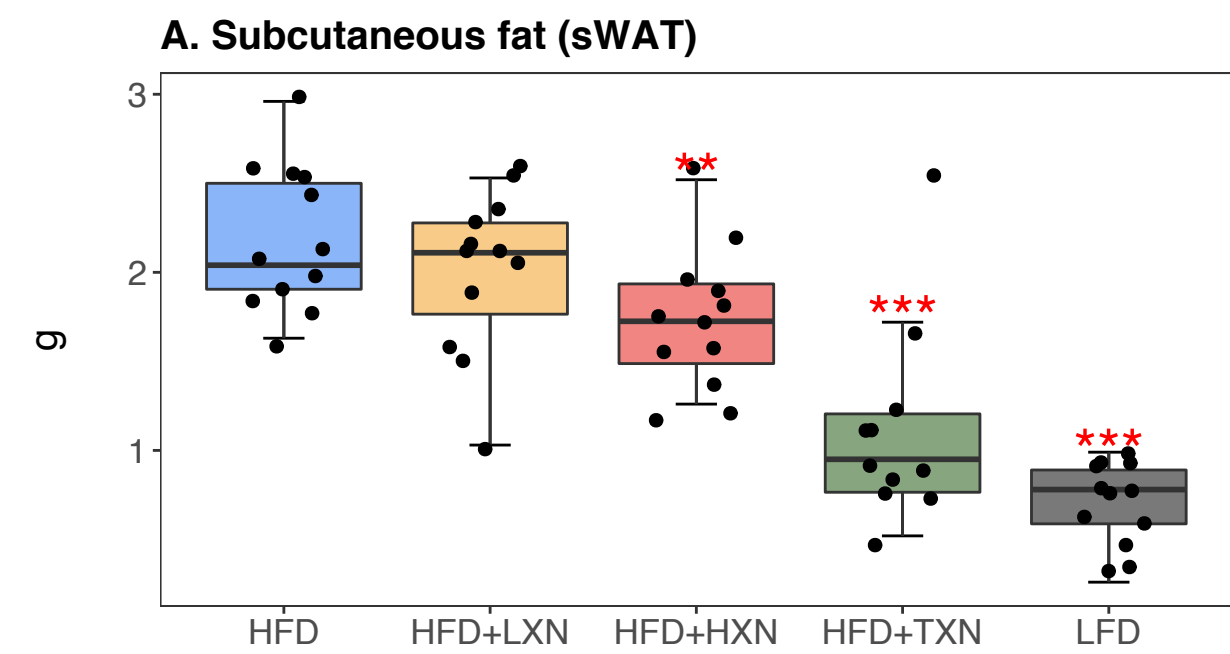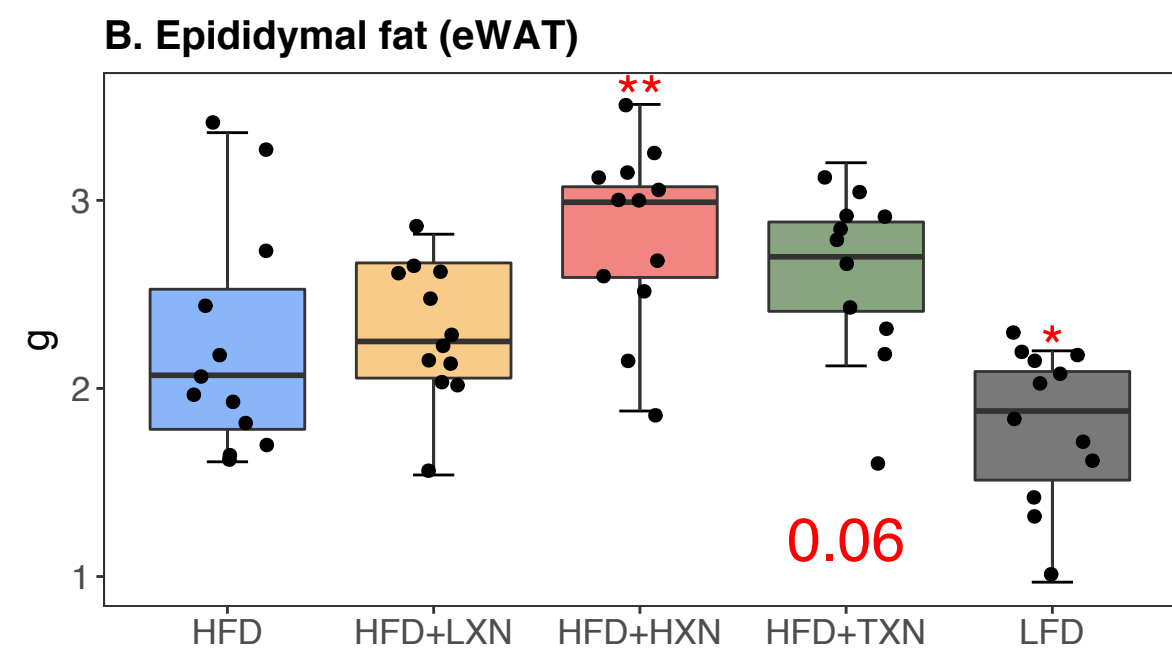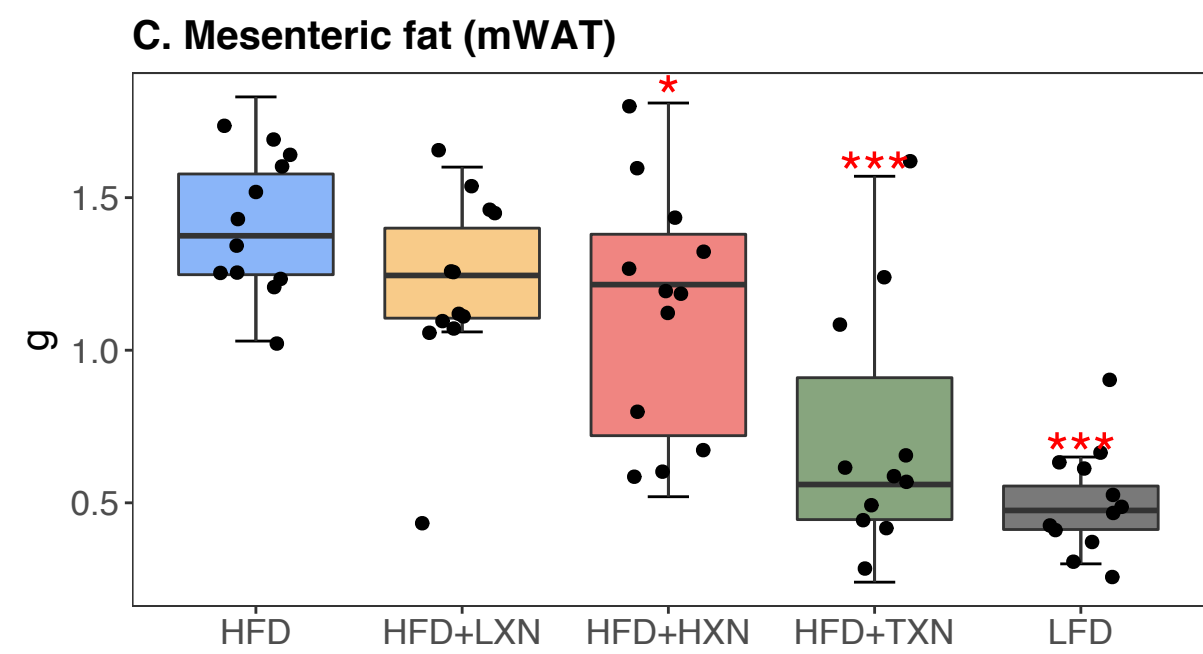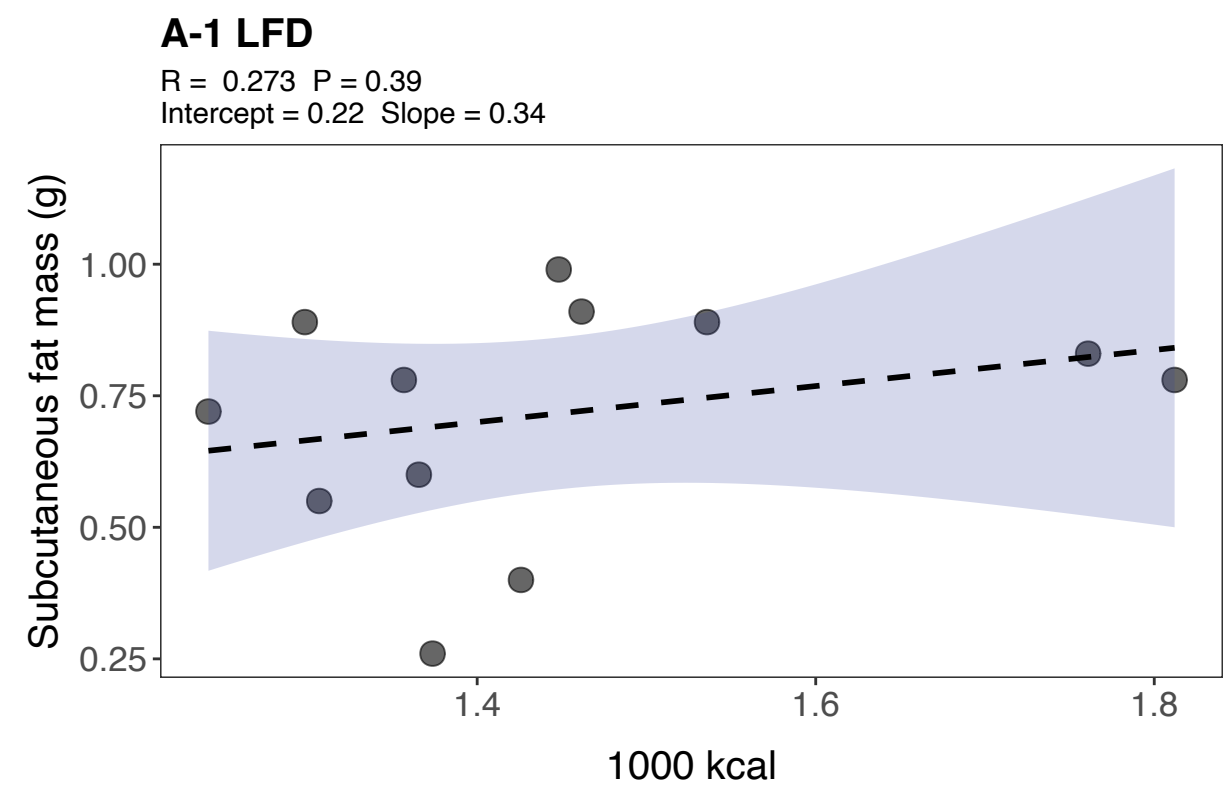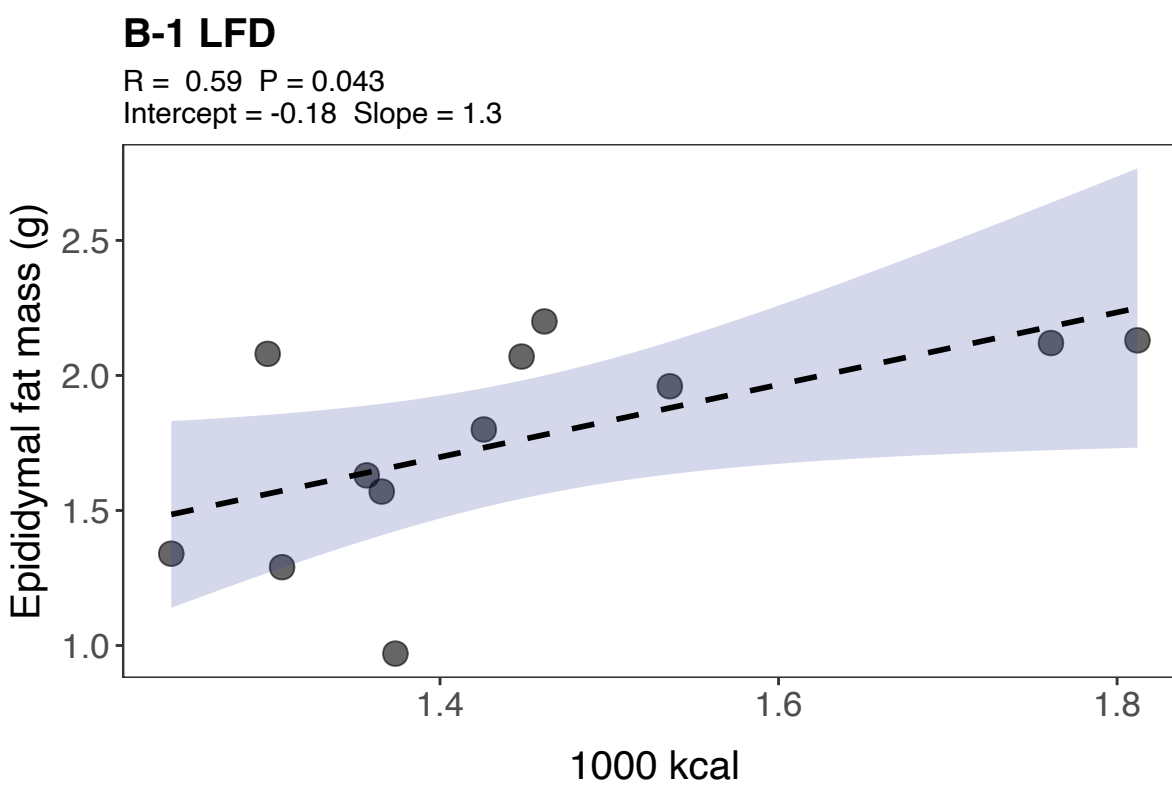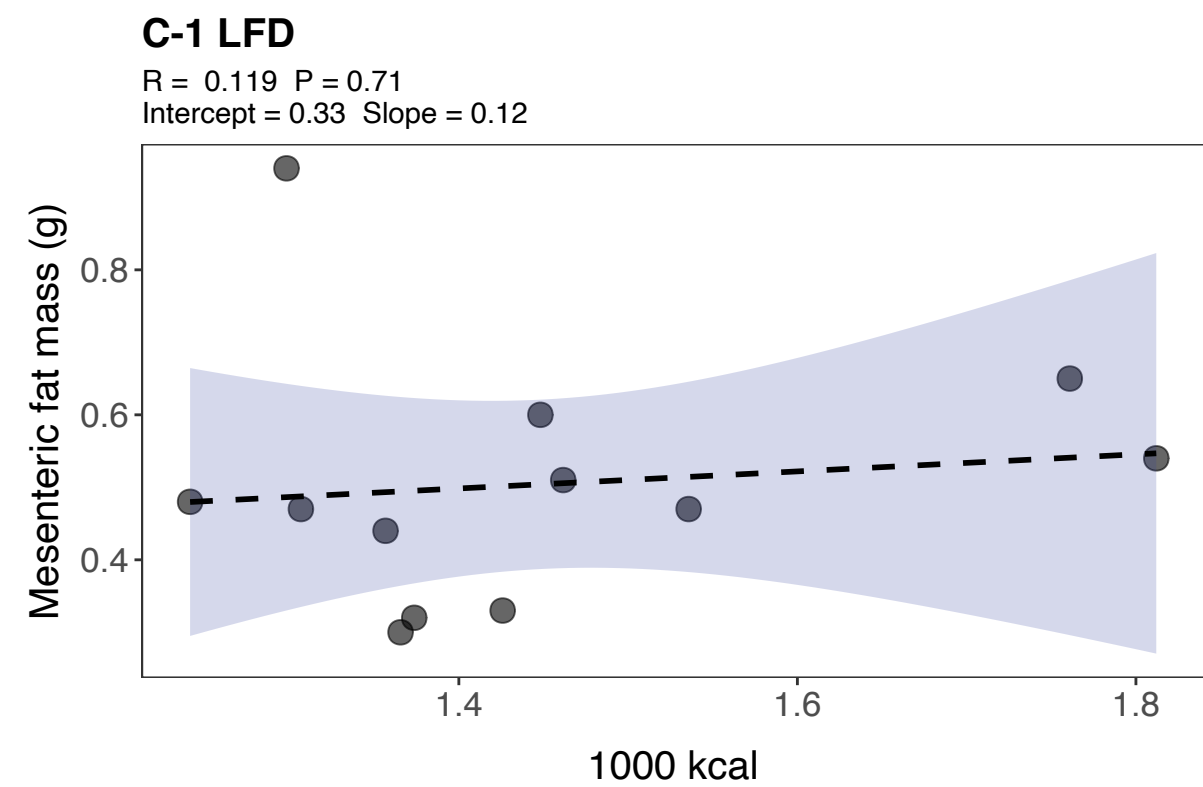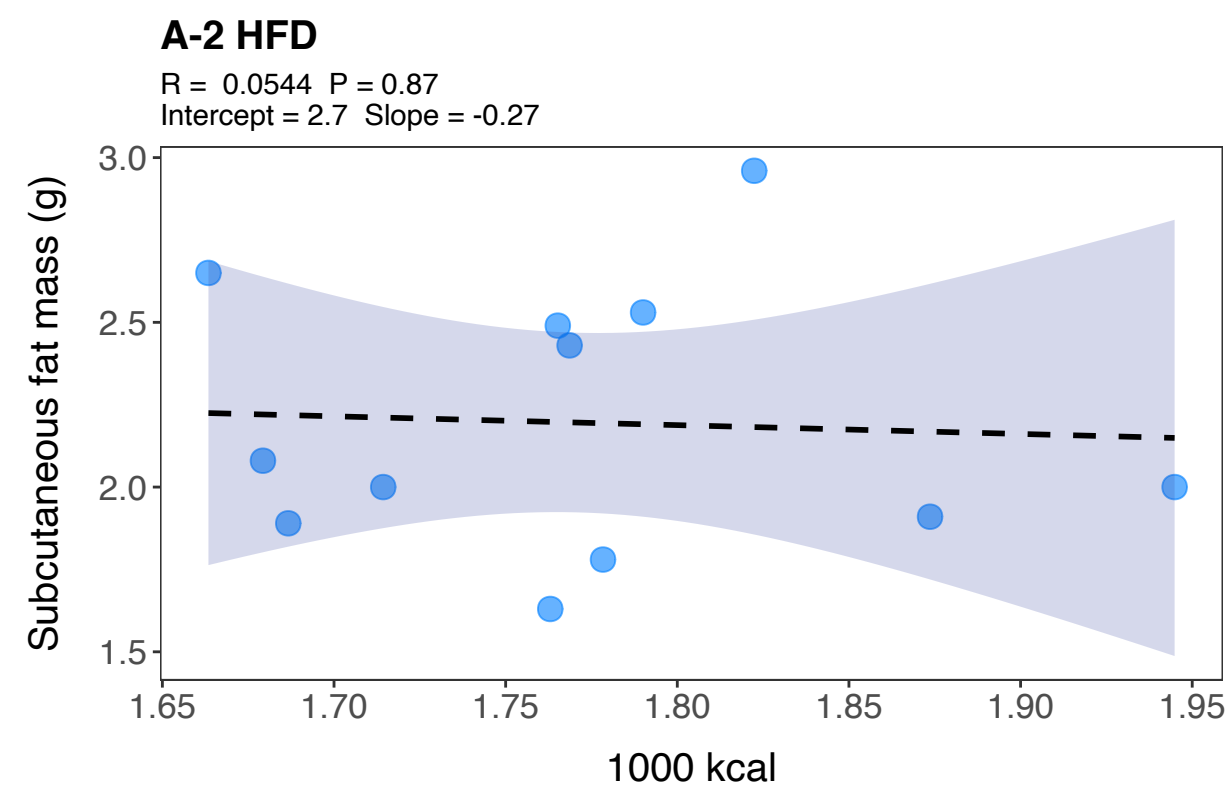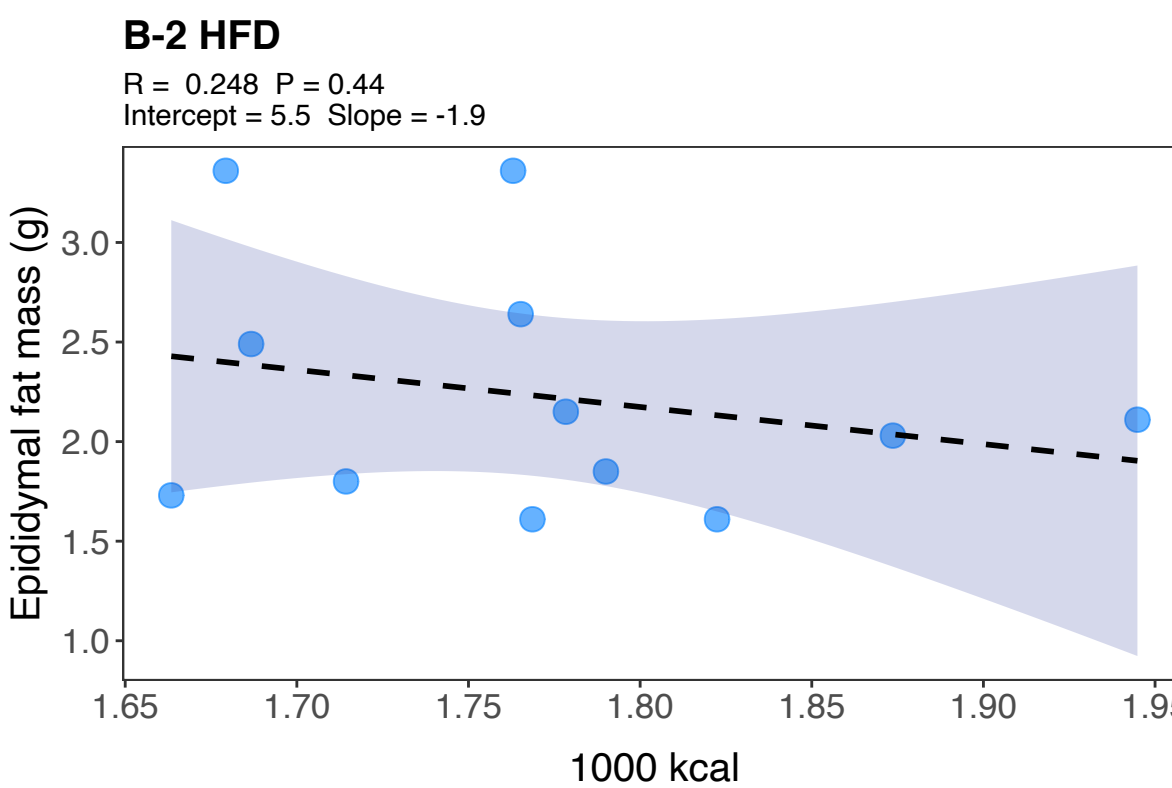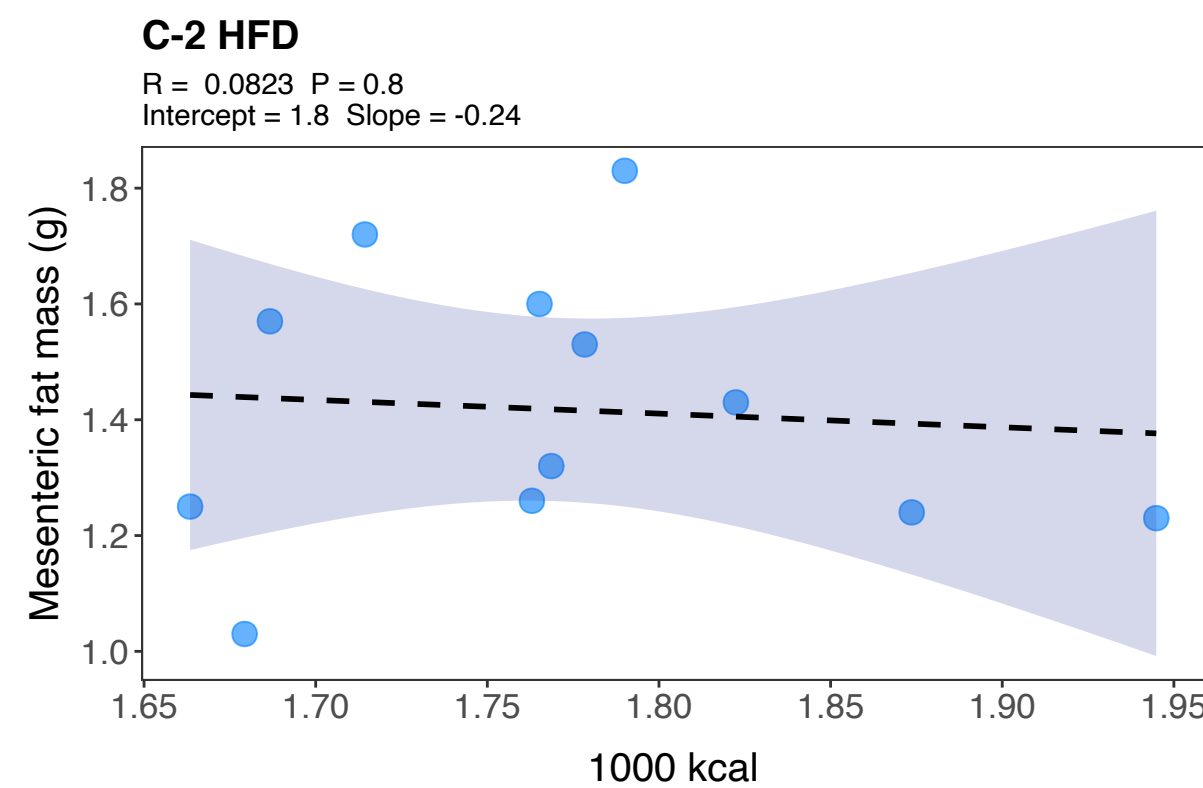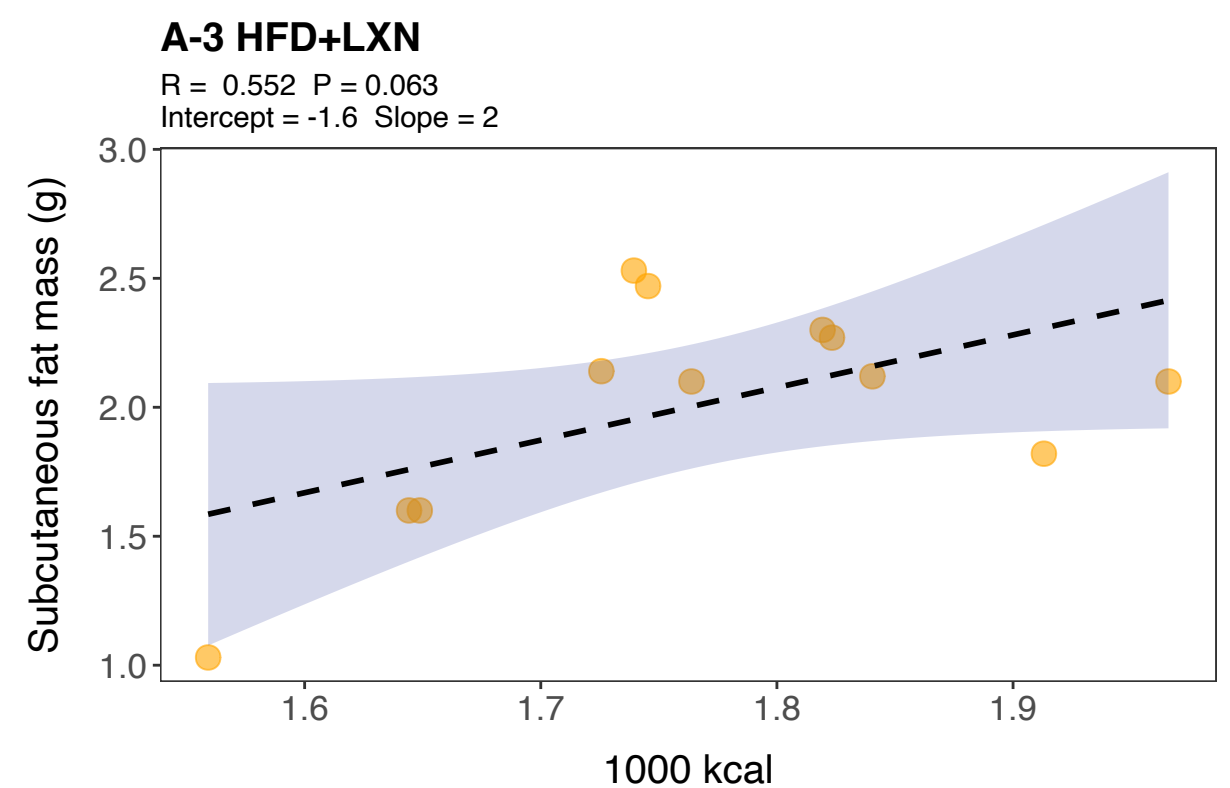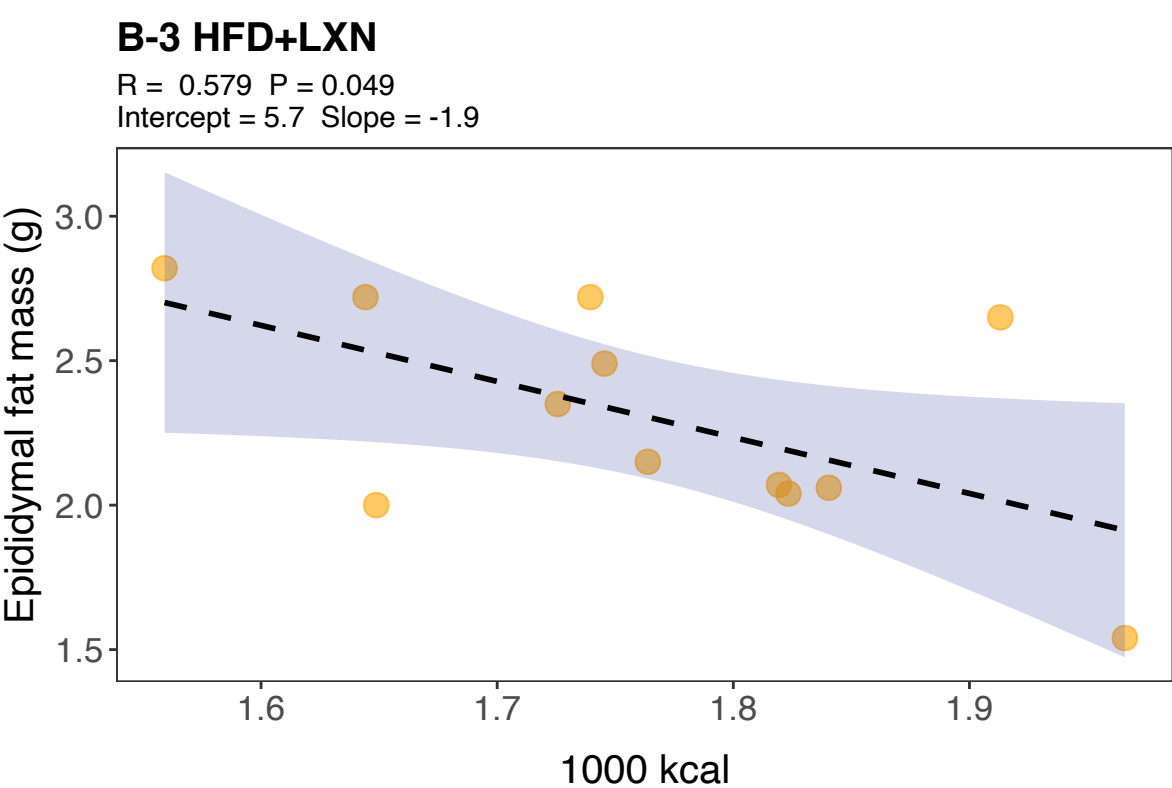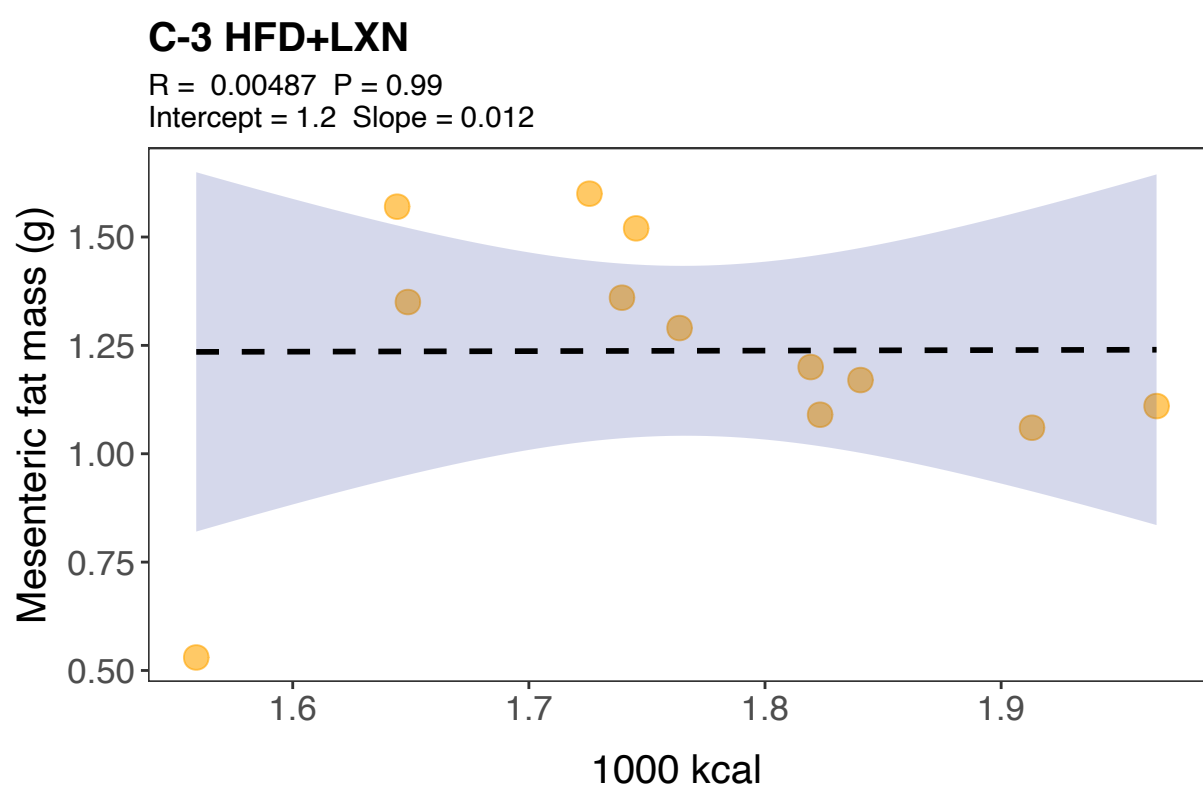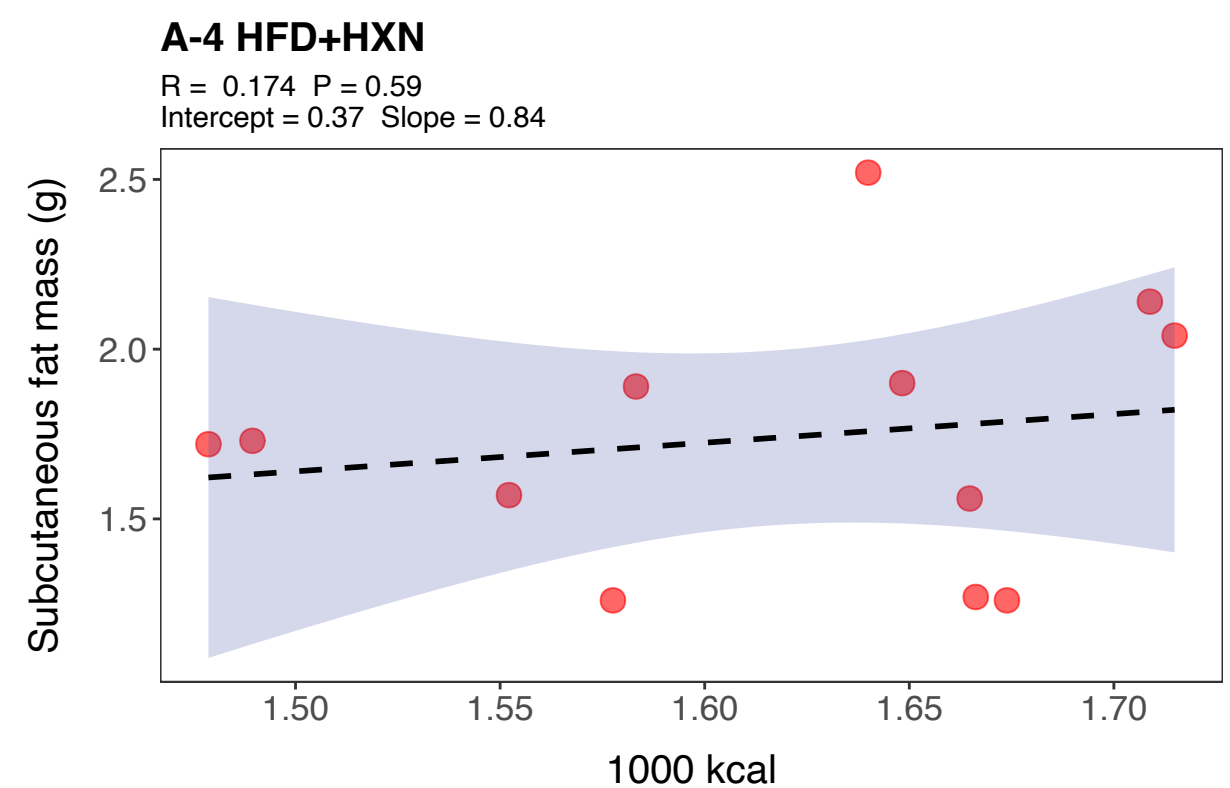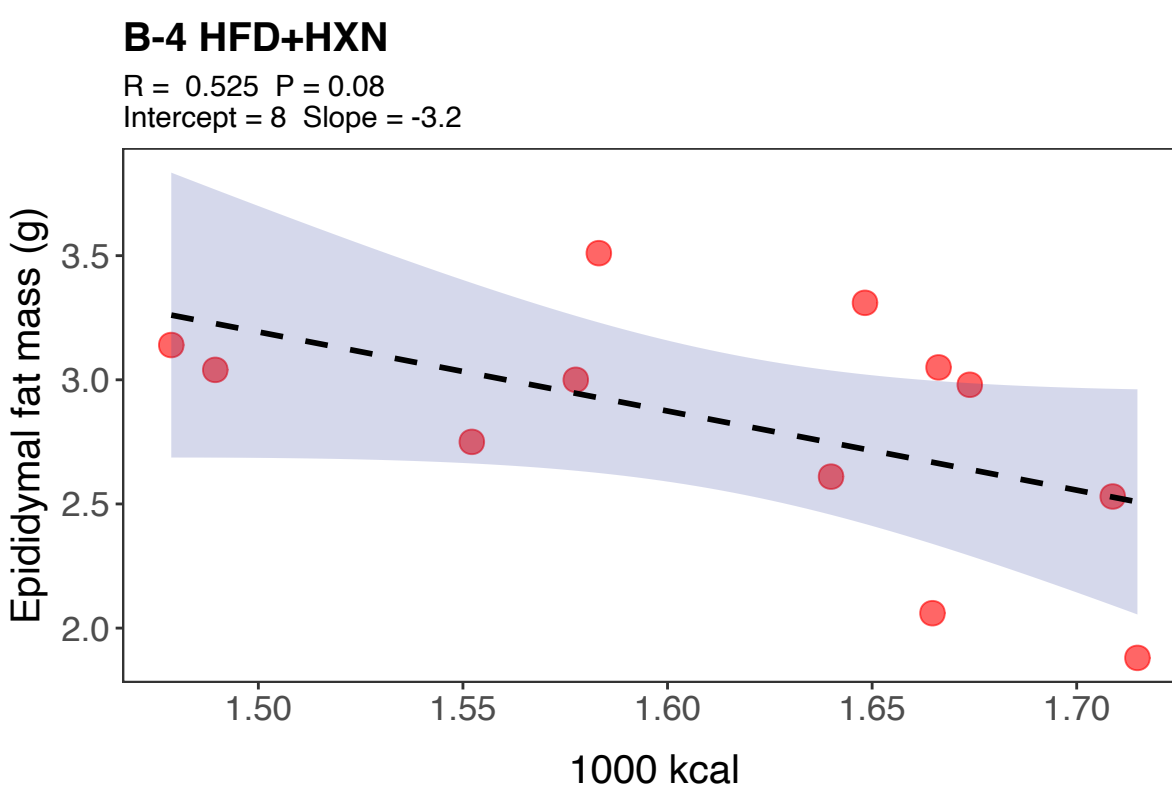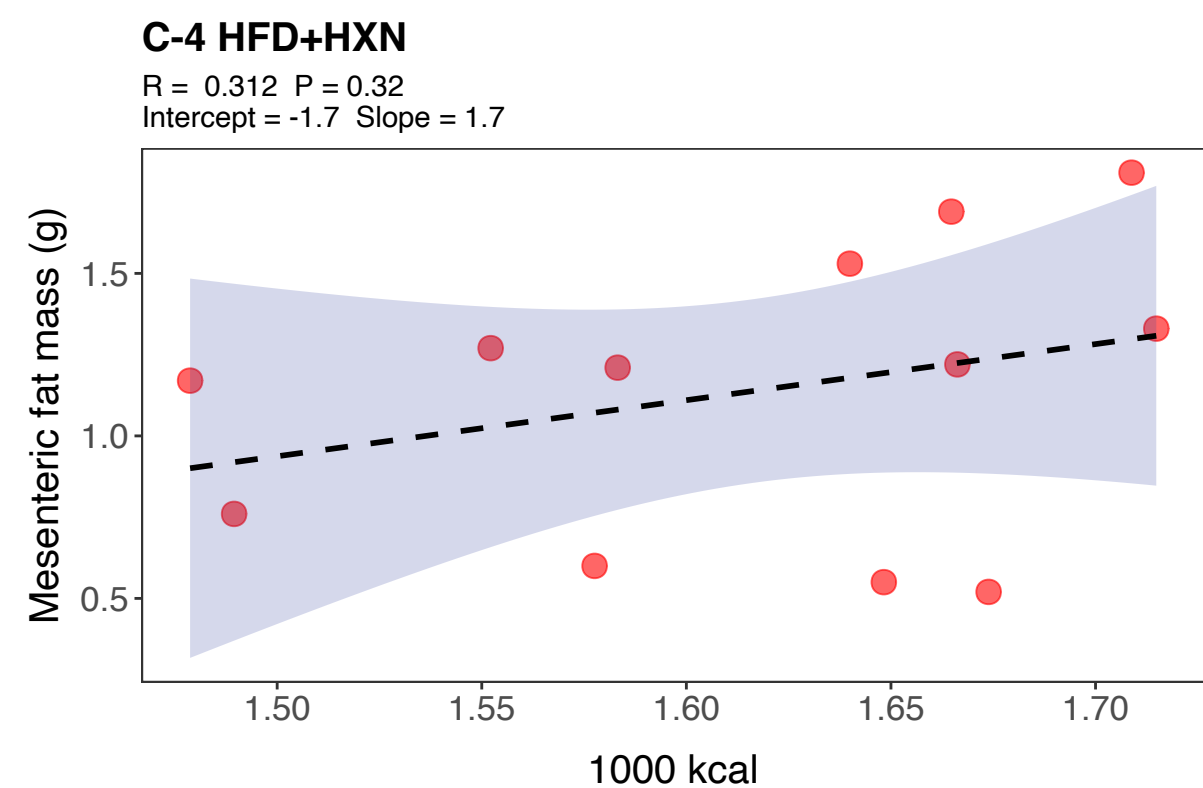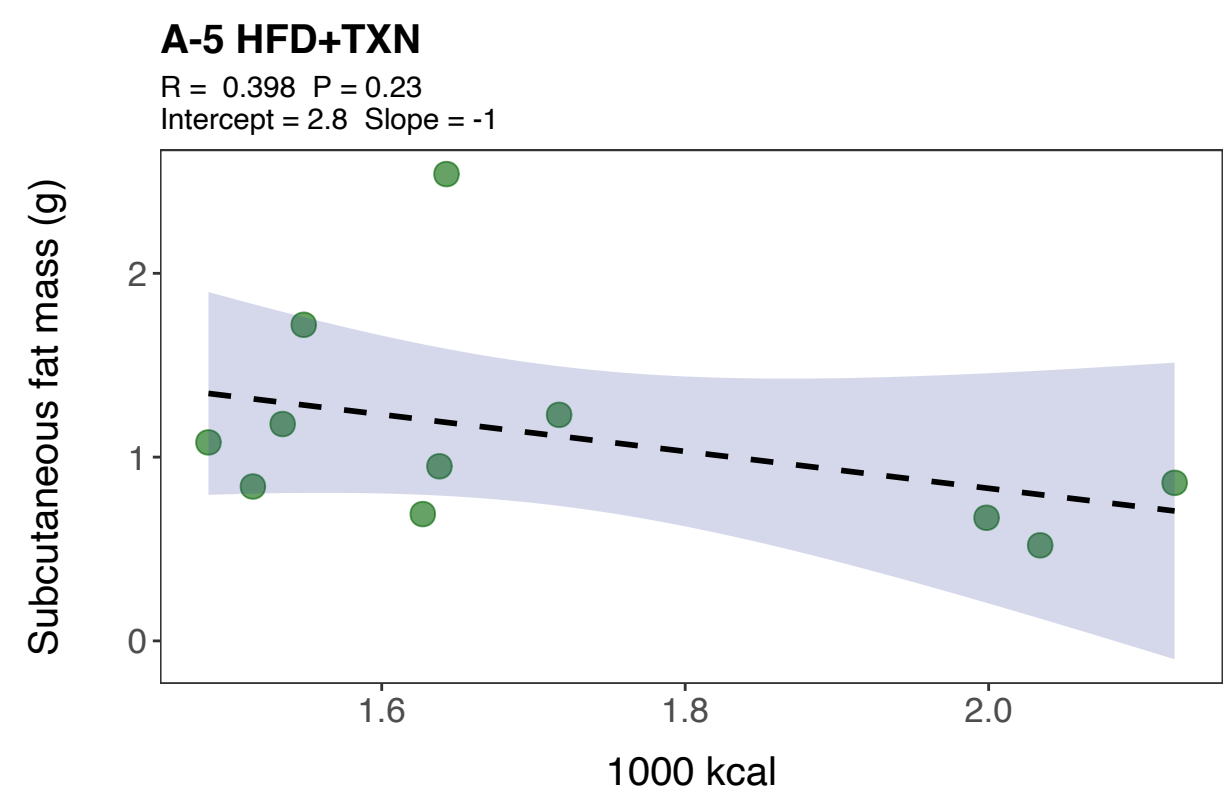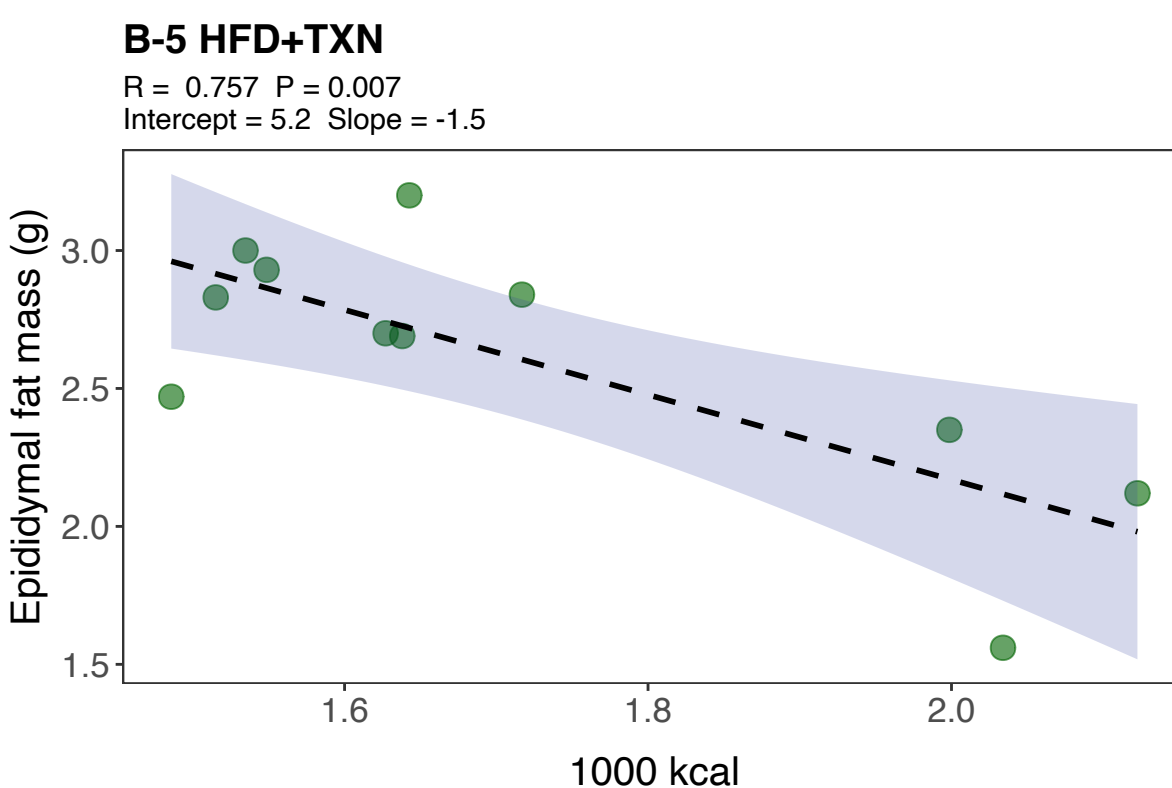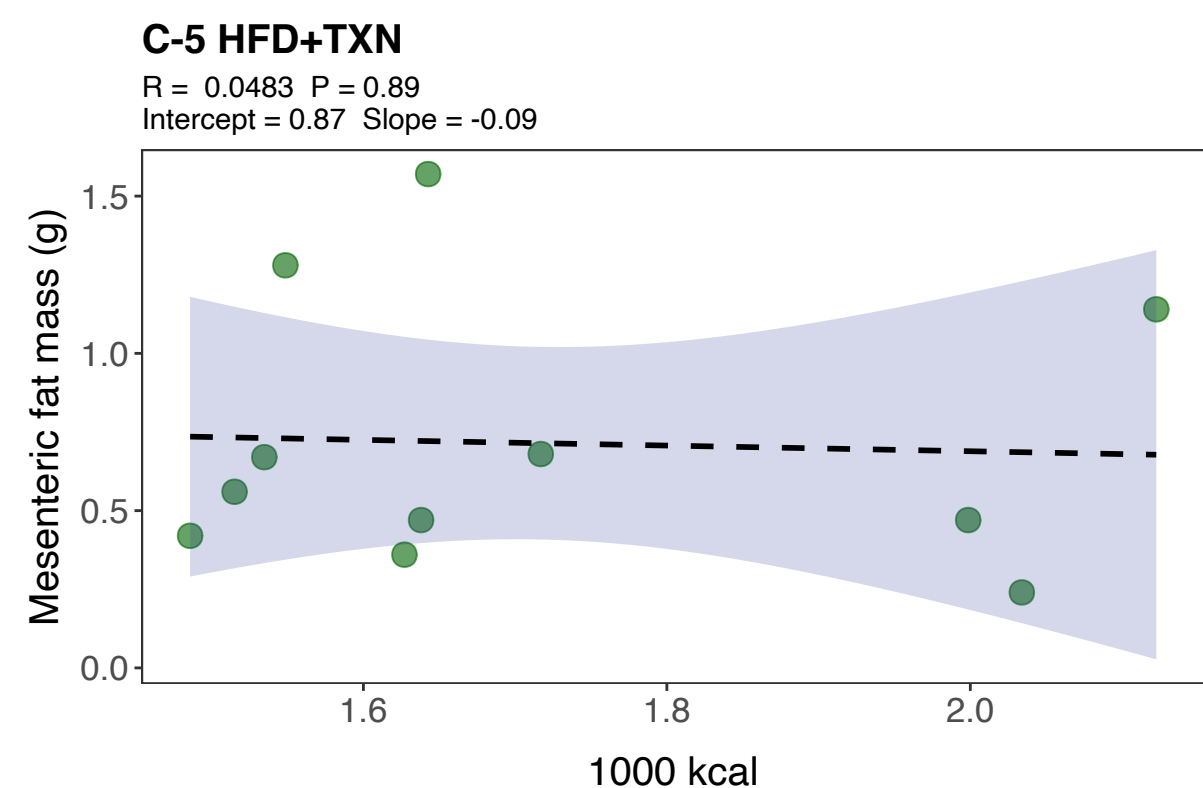

Supplement: Figure 5—source data 1. — This zip archive contains the following: (1) One Comma Separated Values file named ‘fig5_table.csv’ phenotypic data directly pertaining to Figure 5. (2) An R script file ‘ggplotRegression.R’. (3) A Jupyter Notebook file contains scripts used for statistical analysis and generation of Figure 5. [file elife-66398-fig5-data1.zip › Figure5/figure5.pdf]

# Liver mass relative to body weight

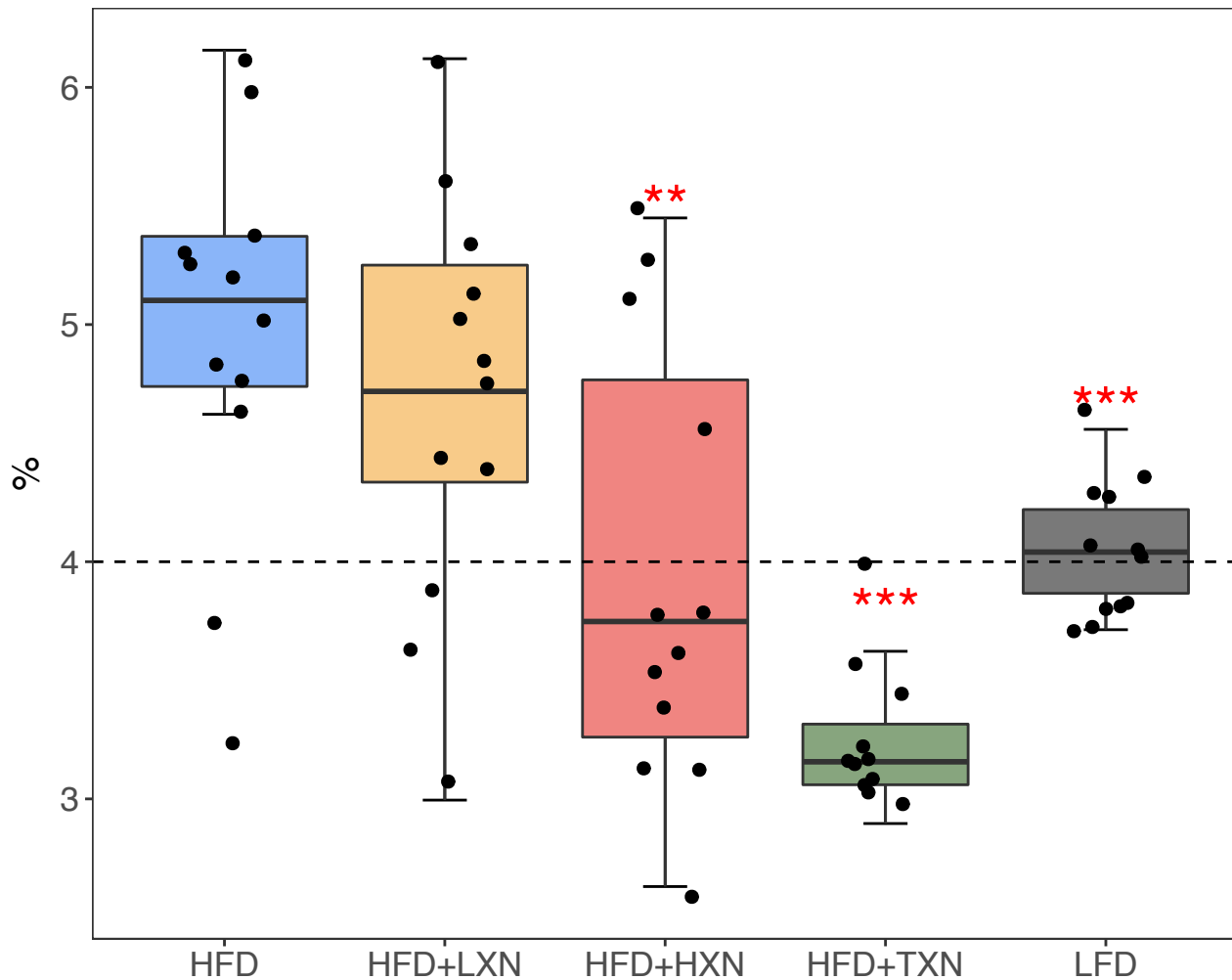

Supplement: Figure 6—source data 1. — A folder called “TXN prevents HFD-induced liver steatosis in mice” containing histology images in TIFF format (n = 59), used for histology scoring and Excel spreadsheet with scores and sample IDs. Figshare link that contains raw images: https://doi.org/10.6084/m9.figshare.13619273. [file elife-66398-fig6-data1.zip › Figure6/B.pdf]

# Liver TAG

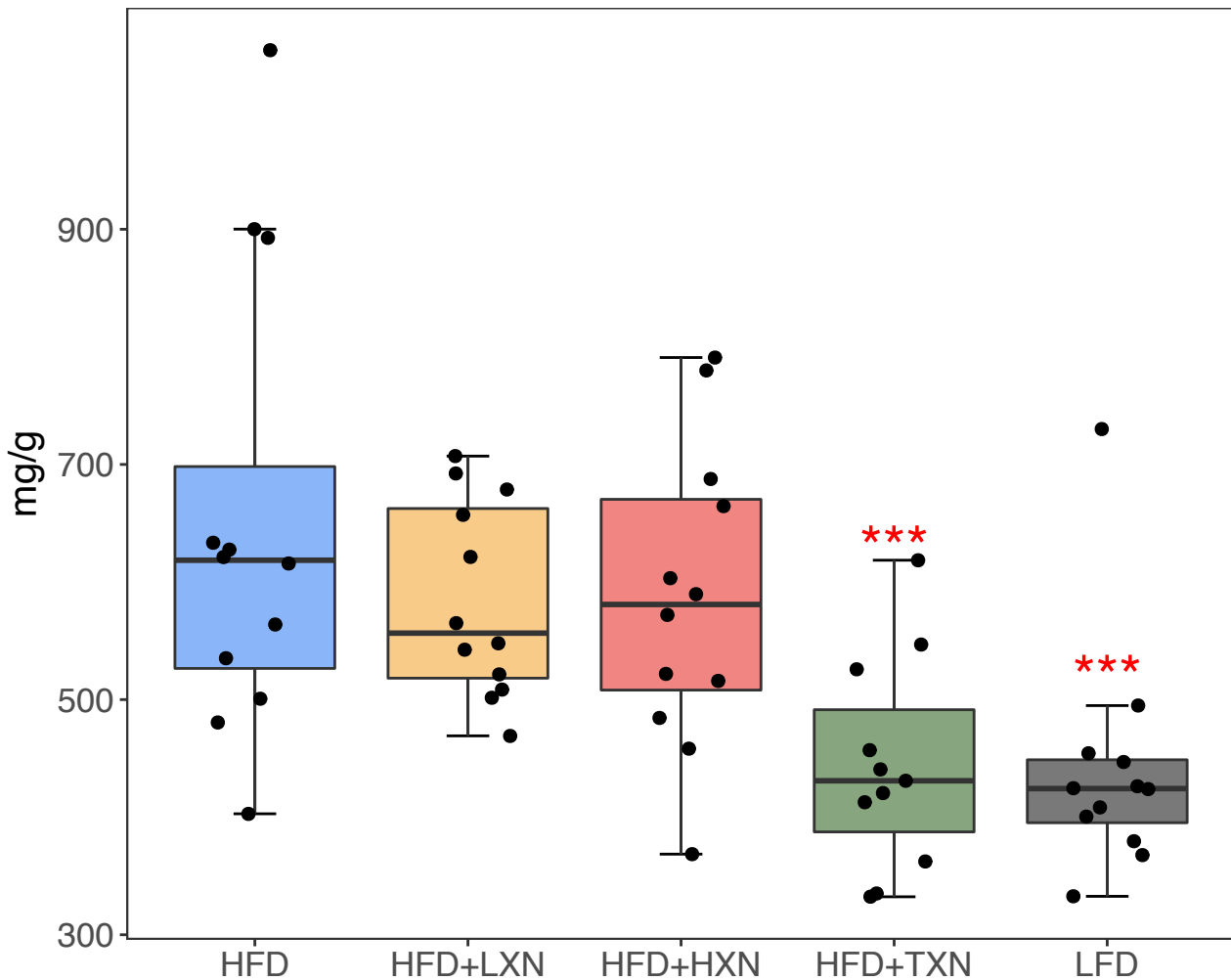

Supplement: Figure 6—source data 1. — A folder called “TXN prevents HFD-induced liver steatosis in mice” containing histology images in TIFF format (n = 59), used for histology scoring and Excel spreadsheet with scores and sample IDs. Figshare link that contains raw images: https://doi.org/10.6084/m9.figshare.13619273. [file elife-66398-fig6-data1.zip › Figure6/C.pdf]

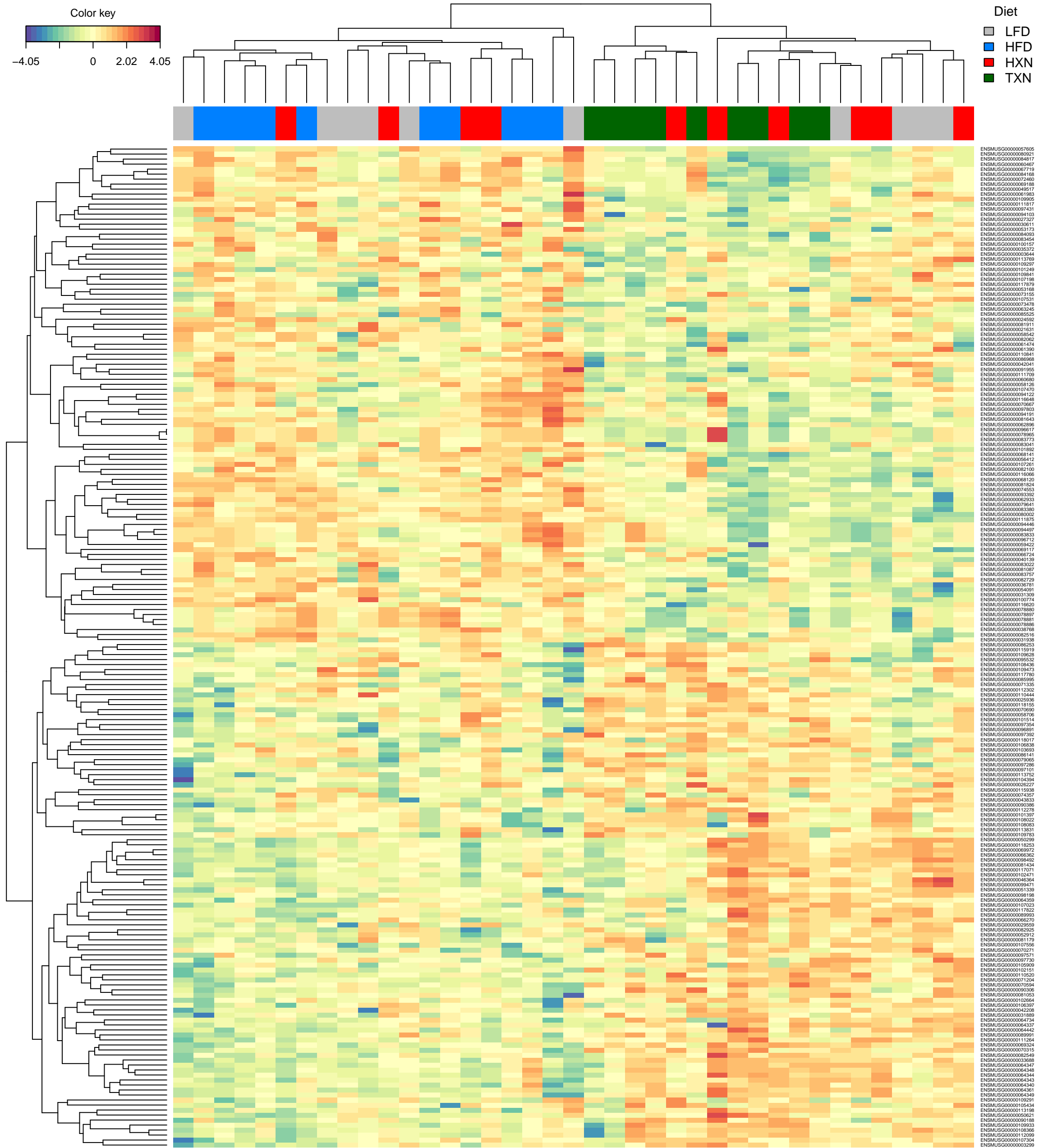

Supplement: Figure 7—source data 1. — This zip archive contains the following: (1) A Jupyter Notebook file contains scripts used for statistical analysis and generation of Figure 7. (2) A R object file in Rds format named ‘y_keep.rds’. (3) An R script used to generate the ‘y_keep.rds’ file. [file elife-66398-fig7-data1.zip › Figure7/heatmap.pdf]

# HXN vs. HFD

## Number of DEGs: 6

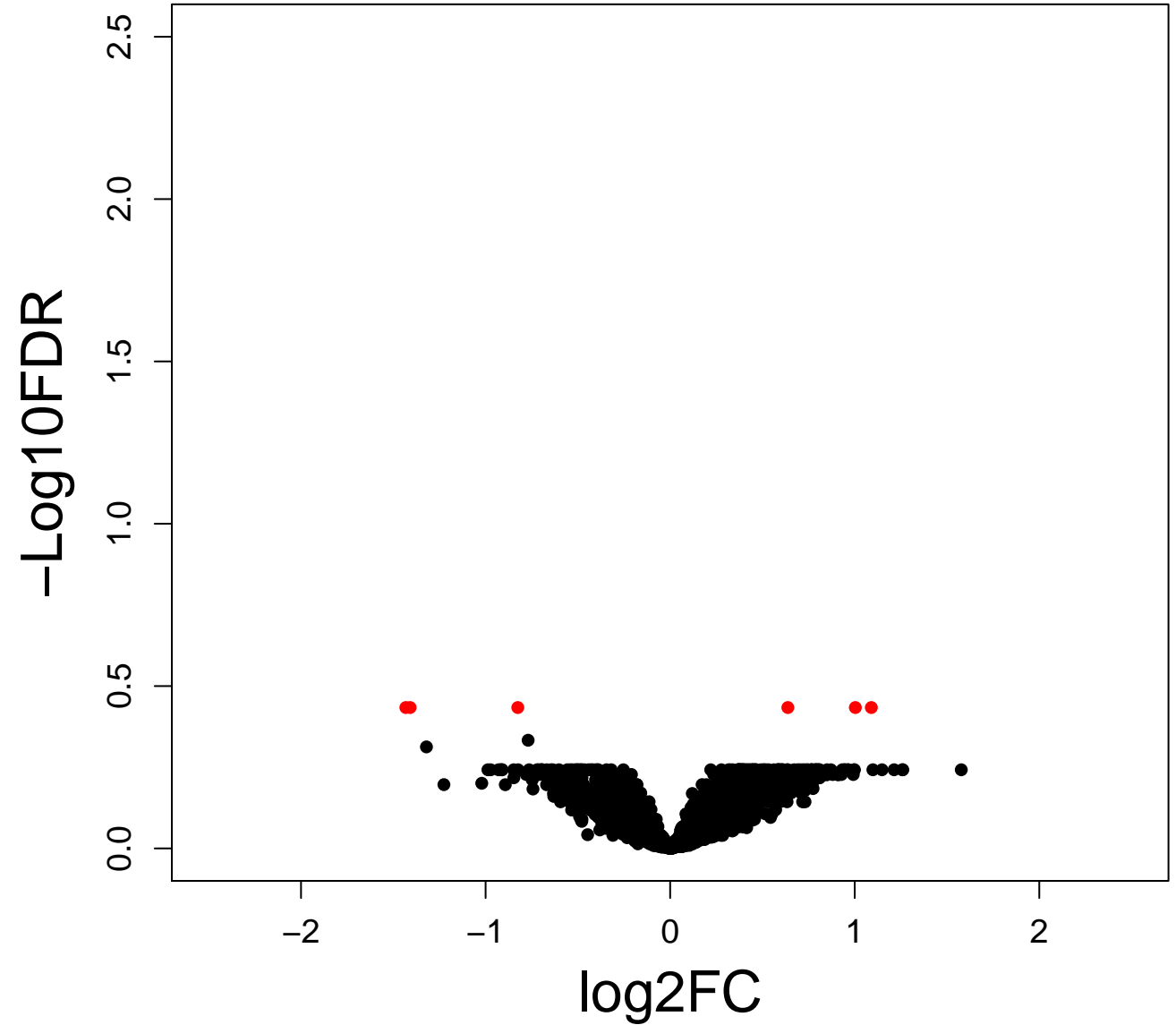

Supplement: Figure 7—source data 1. — This zip archive contains the following: (1) A Jupyter Notebook file contains scripts used for statistical analysis and generation of Figure 7. (2) A R object file in Rds format named ‘y_keep.rds’. (3) An R script used to generate the ‘y_keep.rds’ file. [file elife-66398-fig7-data1.zip › Figure7/volplot-hxn-hfd.pdf]

# LFD vs. HFD

## Number of DEGs: 212

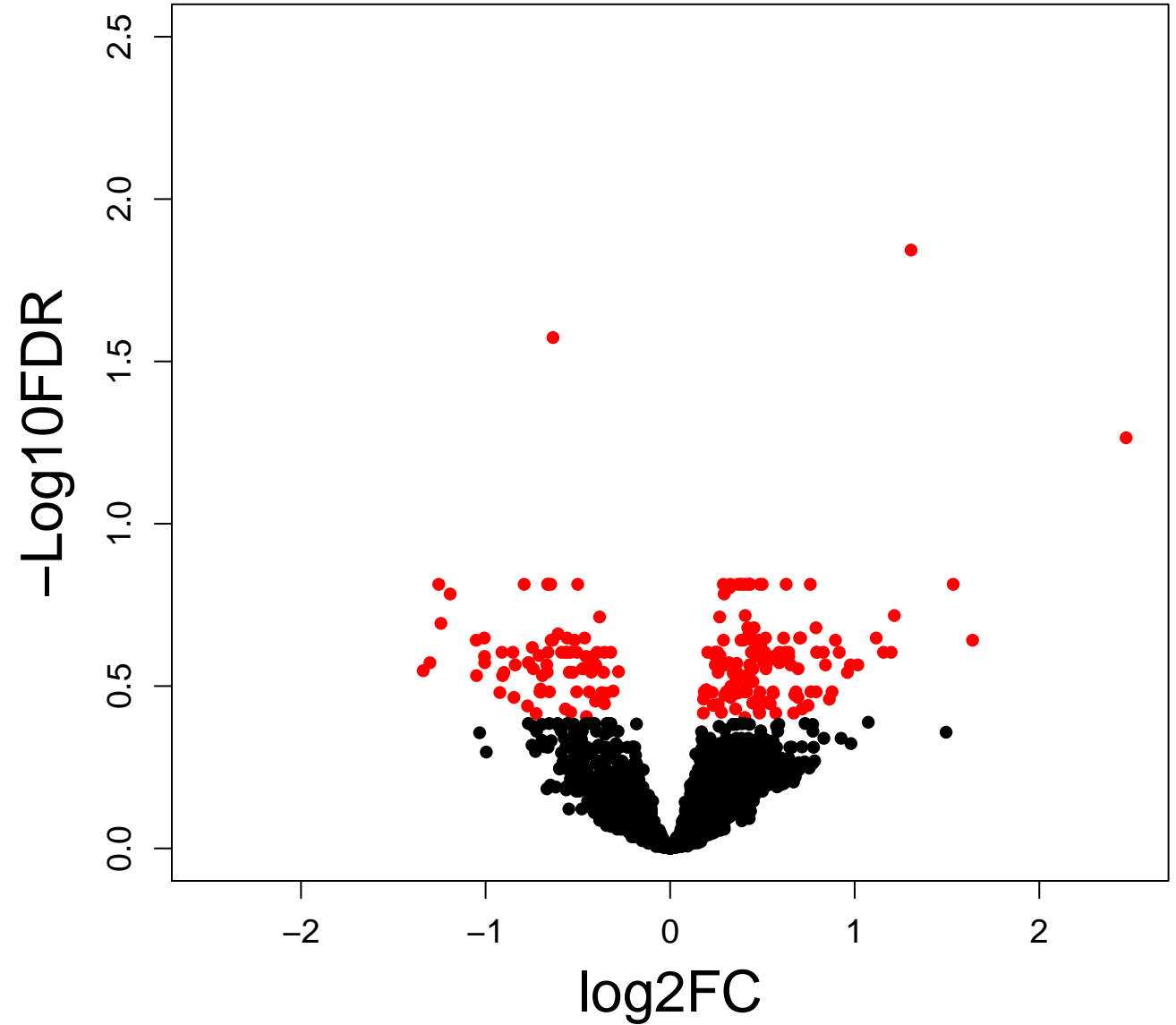

Supplement: Figure 7—source data 1. — This zip archive contains the following: (1) A Jupyter Notebook file contains scripts used for statistical analysis and generation of Figure 7. (2) A R object file in Rds format named ‘y_keep.rds’. (3) An R script used to generate the ‘y_keep.rds’ file. [file elife-66398-fig7-data1.zip › Figure7/volplot-lfd-hfd.pdf]

# TXN vs. HFD

Number of DEGs: 295

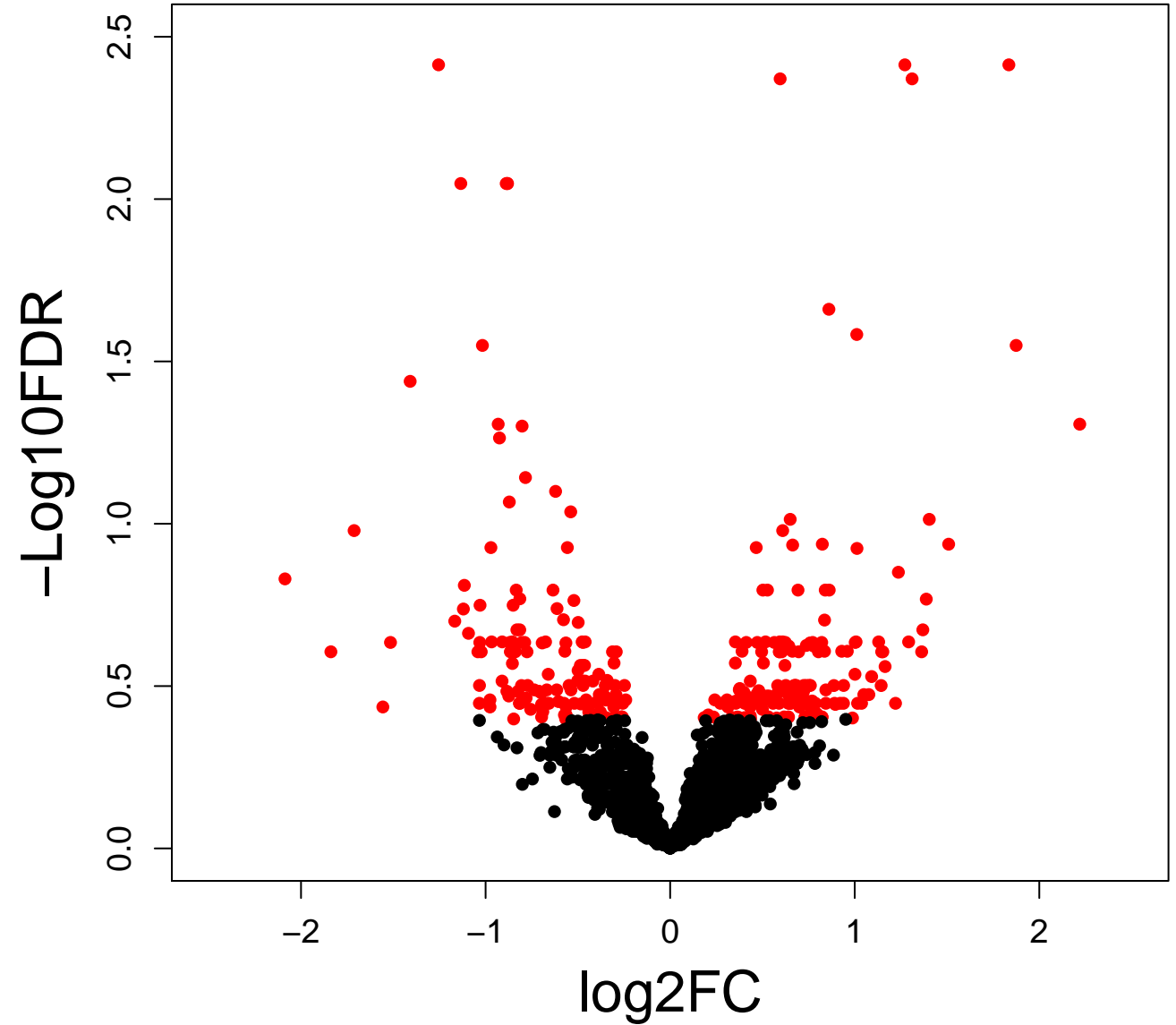

Supplement: Figure 7—source data 1. — This zip archive contains the following: (1) A Jupyter Notebook file contains scripts used for statistical analysis and generation of Figure 7. (2) A R object file in Rds format named ‘y_keep.rds’. (3) An R script used to generate the ‘y_keep.rds’ file. [file elife-66398-fig7-data1.zip › Figure7/volplot-txn-hfd.pdf]

| RReliefF importance | Number of features |
|---------------------|--------------------|
| 0.1                 | 0                  |
| 0.19                | 10                 |
| 0.2                 | 20                 |
| 0.24                | 30                 |
| 0.26                | 40                 |
| 0.31                | 50                 |
| 0.31                | 60                 |
| 0.32                | 70                 |
| 0.38                | 80                 |
| 0.50                | 90                 |
| 0.55                | 100                |
| 0.58                | 110                |
| 0.65                | 120                |

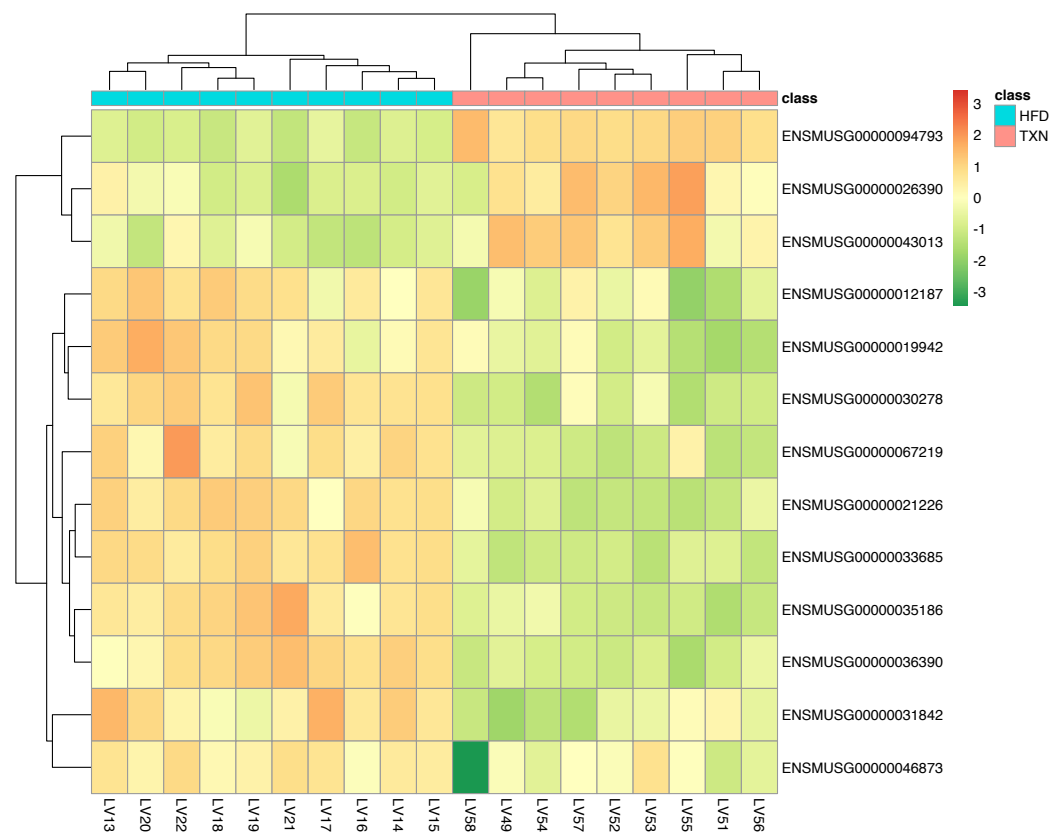

Supplement: Figure 9—source data 1. — This zip archive contains the following: (1) A Comma Separated Values file named ‘colData_hftxn.csv’ contains experiment metadata. (2) A Comma Separated Values file named ‘countMatrix_hftxn.csv’ contains raw counts in HFD and HFD+TXN groups. (3) A tab-delimited text file named ‘dfimportance_hftxn_lgcpm.txt’. (4) A Jupyter Notebook file contains scripts used for statistical analysis and generation of Figure 9. (5) A pdf file named ‘leftPanel.pdf’. (6) A pdf file named ‘rightPanel.pdf’. (7) A PowerPoint file named ‘fig9.pptx’. (8) A pdf file named ‘fig9.pdf’. [file elife-66398-fig9-data1.zip › Figure9/fig9.pdf]

## Slide 1
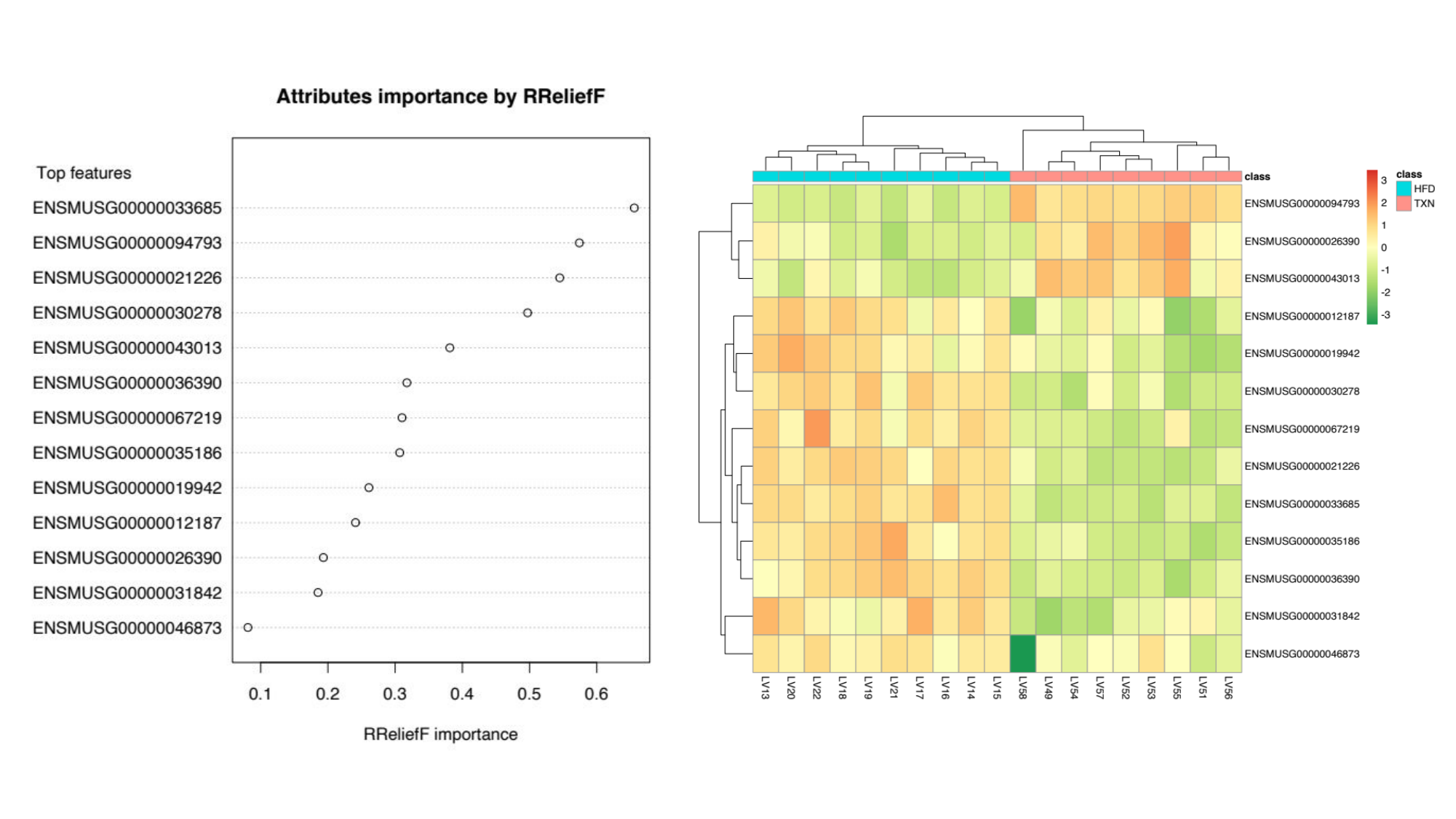

Supplement: Figure 9—source data 1. — This zip archive contains the following: (1) A Comma Separated Values file named ‘colData_hftxn.csv’ contains experiment metadata. (2) A Comma Separated Values file named ‘countMatrix_hftxn.csv’ contains raw counts in HFD and HFD+TXN groups. (3) A tab-delimited text file named ‘dfimportance_hftxn_lgcpm.txt’. (4) A Jupyter Notebook file contains scripts used for statistical analysis and generation of Figure 9. (5) A pdf file named ‘leftPanel.pdf’. (6) A pdf file named ‘rightPanel.pdf’. (7) A PowerPoint file named ‘fig9.pptx’. (8) A pdf file named ‘fig9.pdf’. [file elife-66398-fig9-data1.zip › Figure9/fig9.pptx]

## Attributes importance by RReliefF

Top features

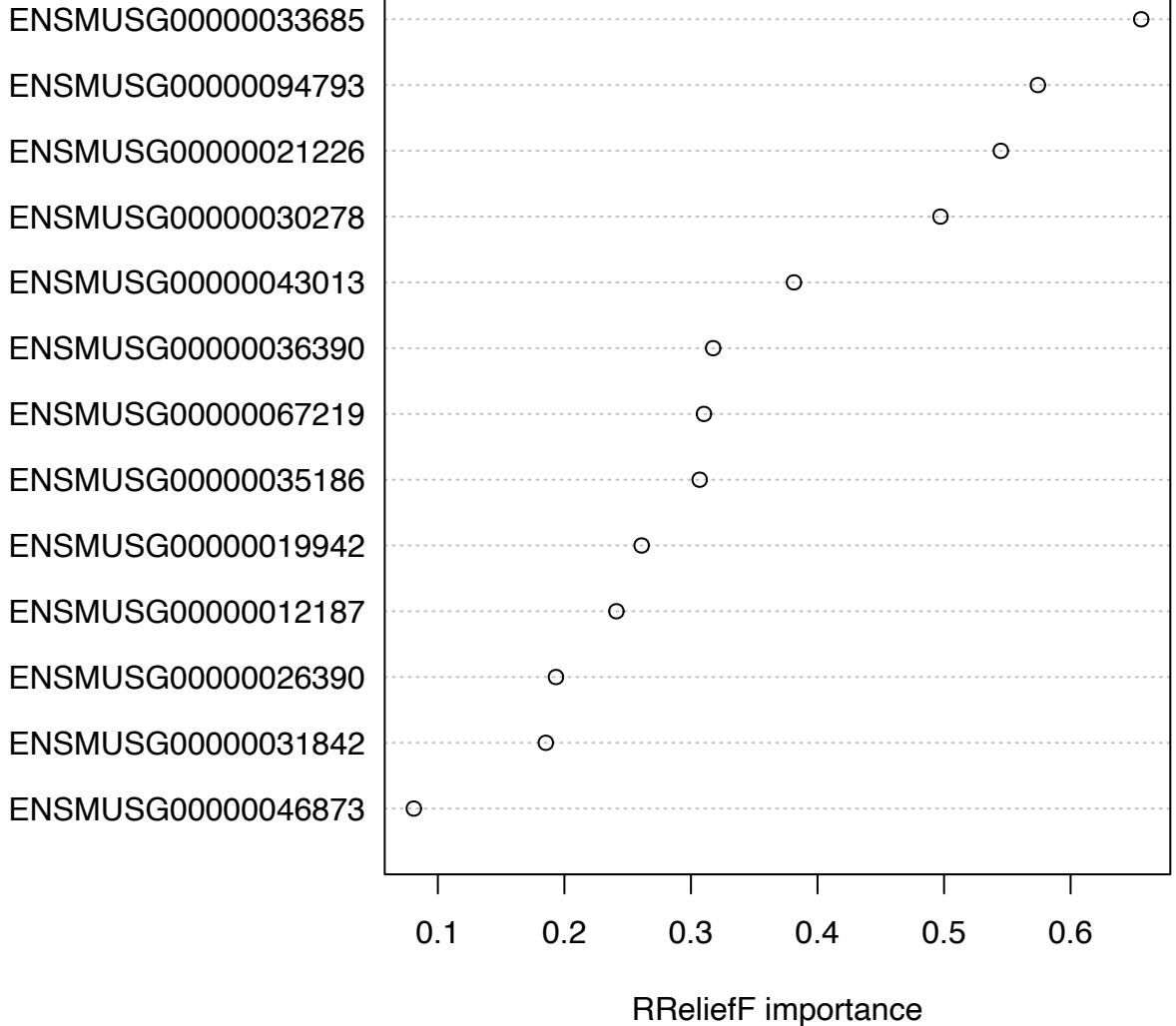

Supplement: Figure 9—source data 1. — This zip archive contains the following: (1) A Comma Separated Values file named ‘colData_hftxn.csv’ contains experiment metadata. (2) A Comma Separated Values file named ‘countMatrix_hftxn.csv’ contains raw counts in HFD and HFD+TXN groups. (3) A tab-delimited text file named ‘dfimportance_hftxn_lgcpm.txt’. (4) A Jupyter Notebook file contains scripts used for statistical analysis and generation of Figure 9. (5) A pdf file named ‘leftPanel.pdf’. (6) A pdf file named ‘rightPanel.pdf’. (7) A PowerPoint file named ‘fig9.pptx’. (8) A pdf file named ‘fig9.pdf’. [file elife-66398-fig9-data1.zip › Figure9/leftPanel.pdf]

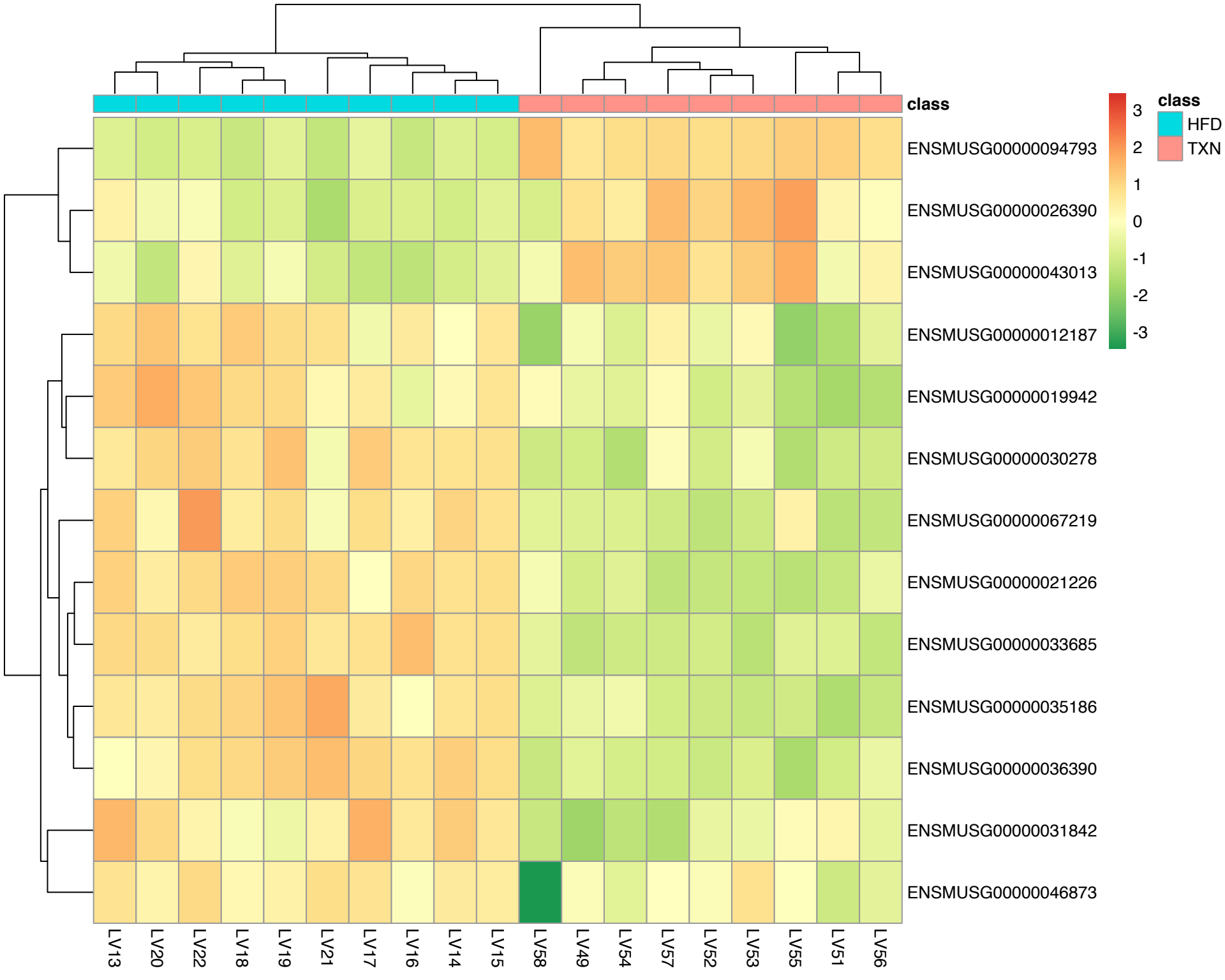

Supplement: Figure 9—source data 1. — This zip archive contains the following: (1) A Comma Separated Values file named ‘colData_hftxn.csv’ contains experiment metadata. (2) A Comma Separated Values file named ‘countMatrix_hftxn.csv’ contains raw counts in HFD and HFD+TXN groups. (3) A tab-delimited text file named ‘dfimportance_hftxn_lgcpm.txt’. (4) A Jupyter Notebook file contains scripts used for statistical analysis and generation of Figure 9. (5) A pdf file named ‘leftPanel.pdf’. (6) A pdf file named ‘rightPanel.pdf’. (7) A PowerPoint file named ‘fig9.pptx’. (8) A pdf file named ‘fig9.pdf’. [file elife-66398-fig9-data1.zip › Figure9/rightPanel.pdf]

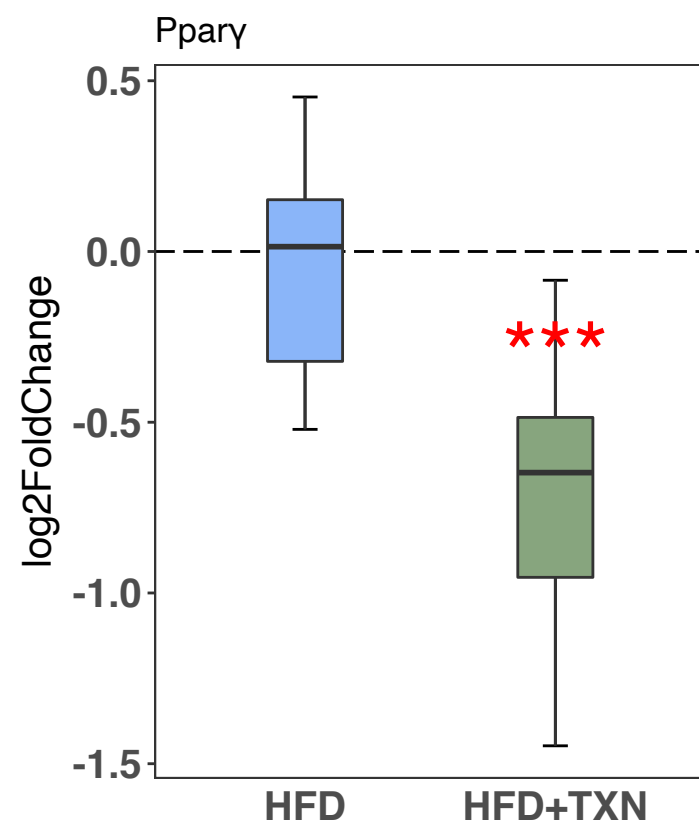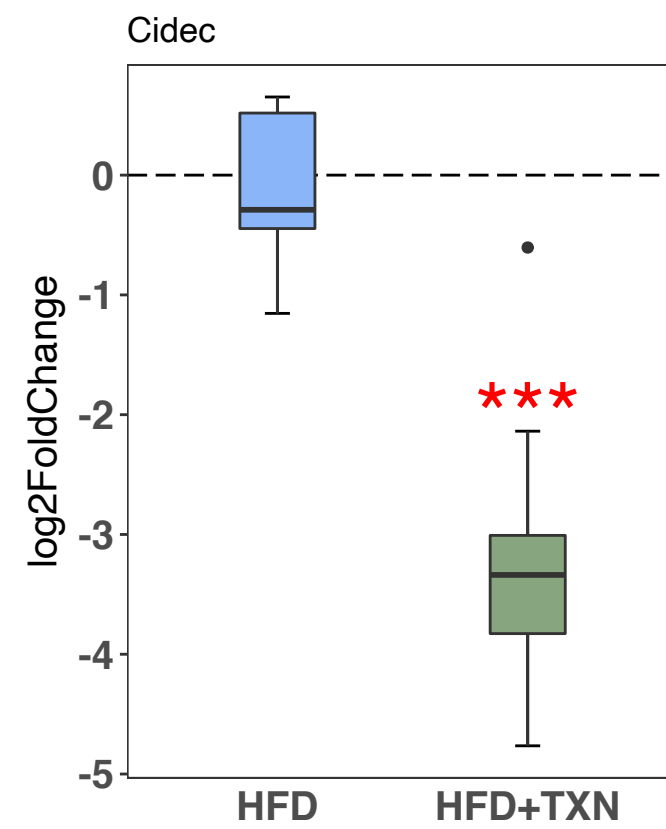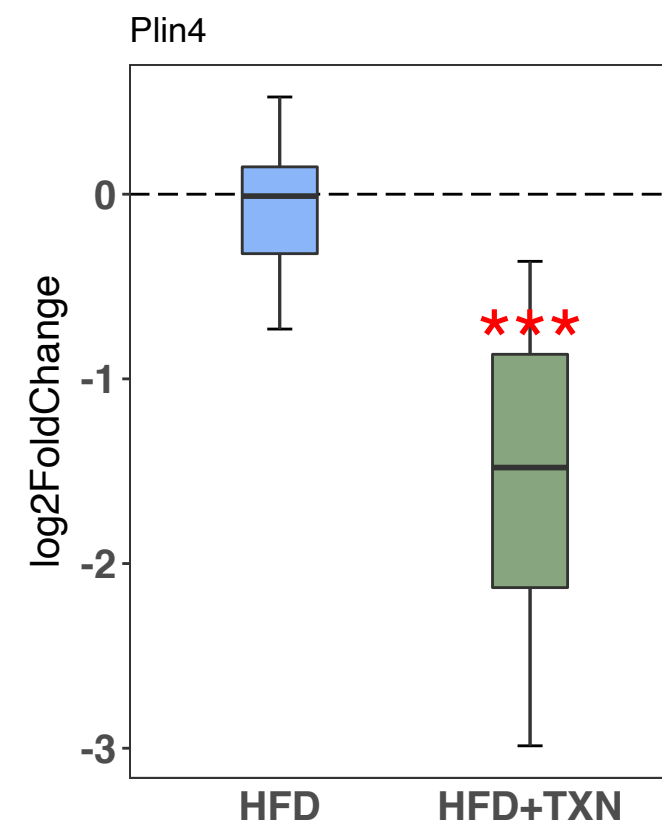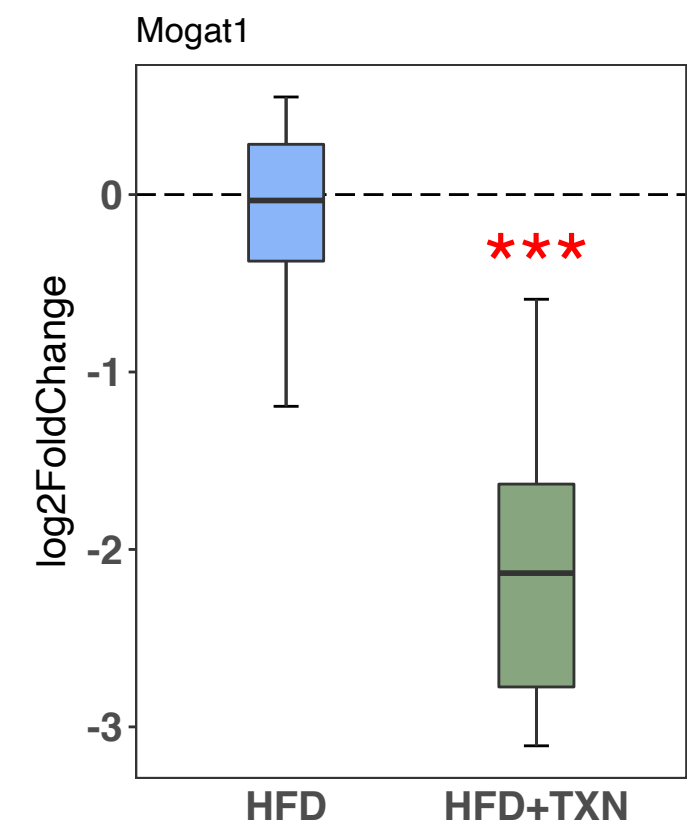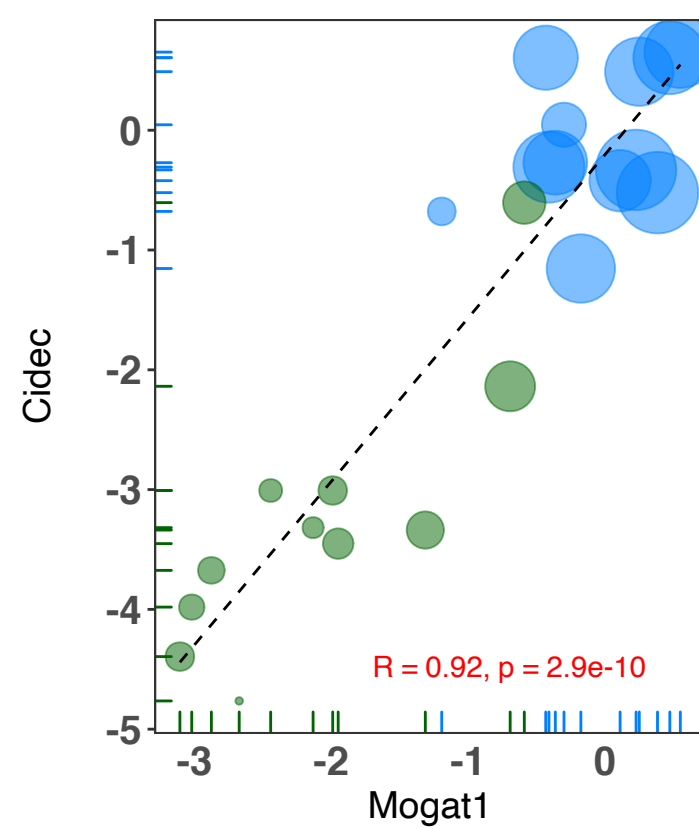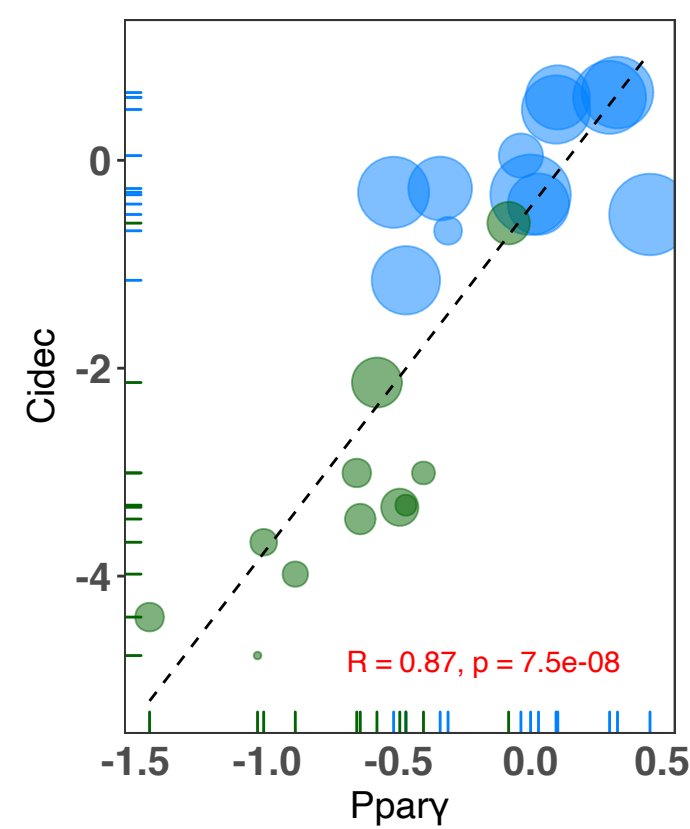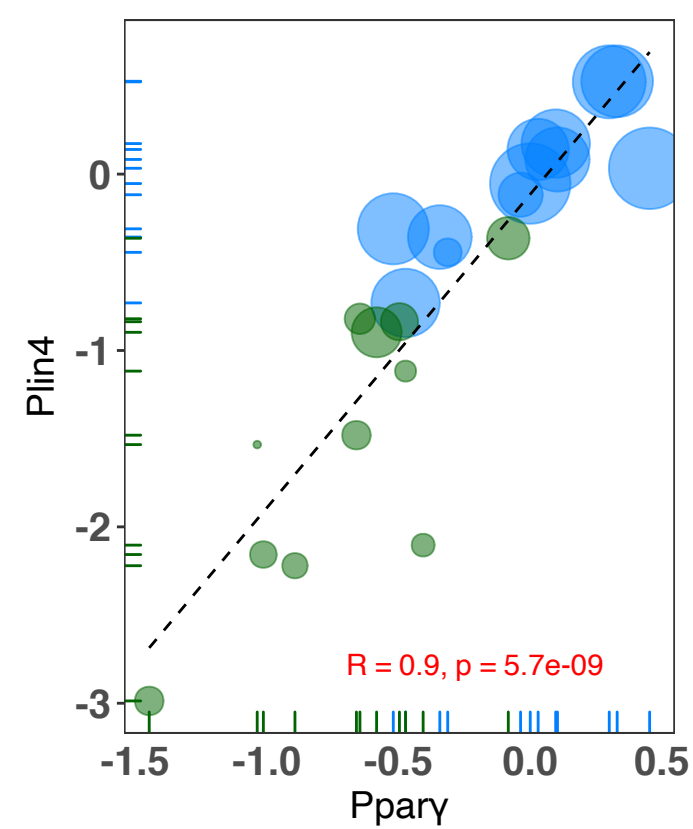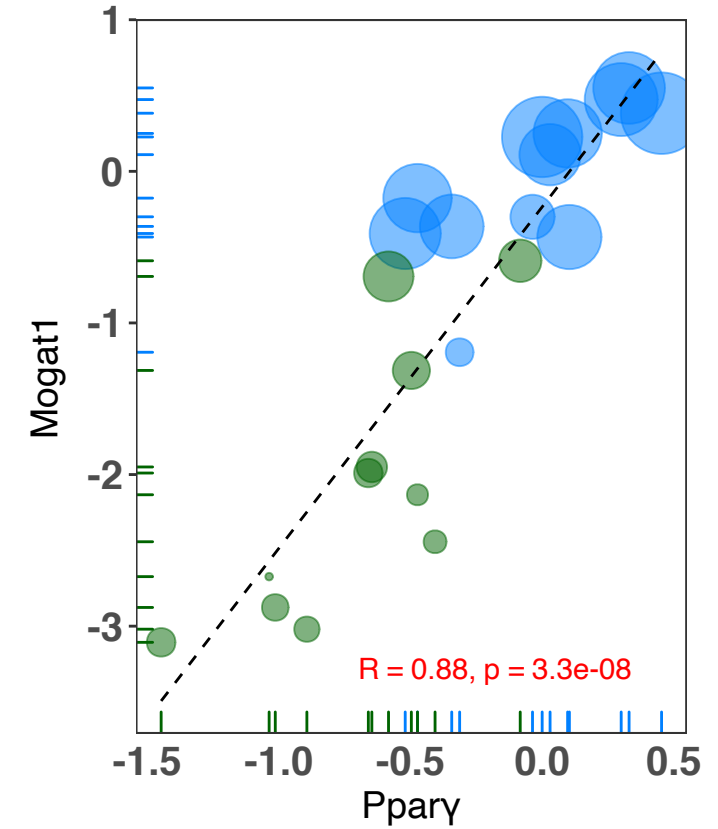

Liver mass:BW (%)

3 4 5 6

Supplement: Figure 10—source data 1. — This zip archive contains the following: (1) A Comma Separated Values file named ‘fig10_table.csv’ phenotypic data directly pertaining to Figure 10. (2) A Excel workbook named ‘PCR_lv_raw.xlsx’ contains raw PCR cycle number data, and the calculation of fold change. (3) A Jupyter Notebook file contains scripts used for statistical analysis and generation of Figure 10. (4) A pdf file named ‘fig10.pdf’. [file elife-66398-fig10-data1.zip › Figure10/figure10.pdf]

A

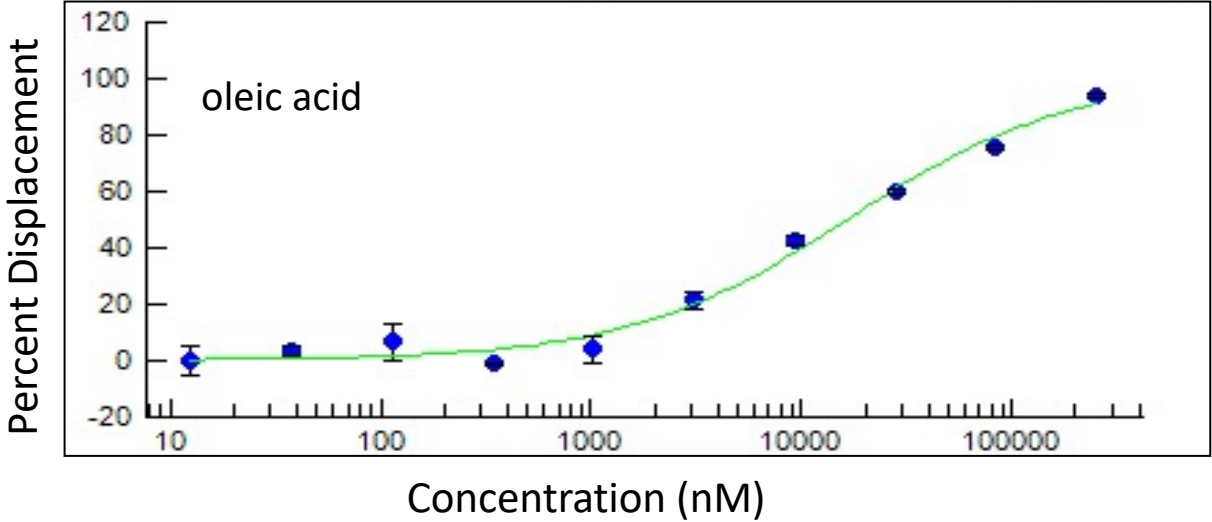

B

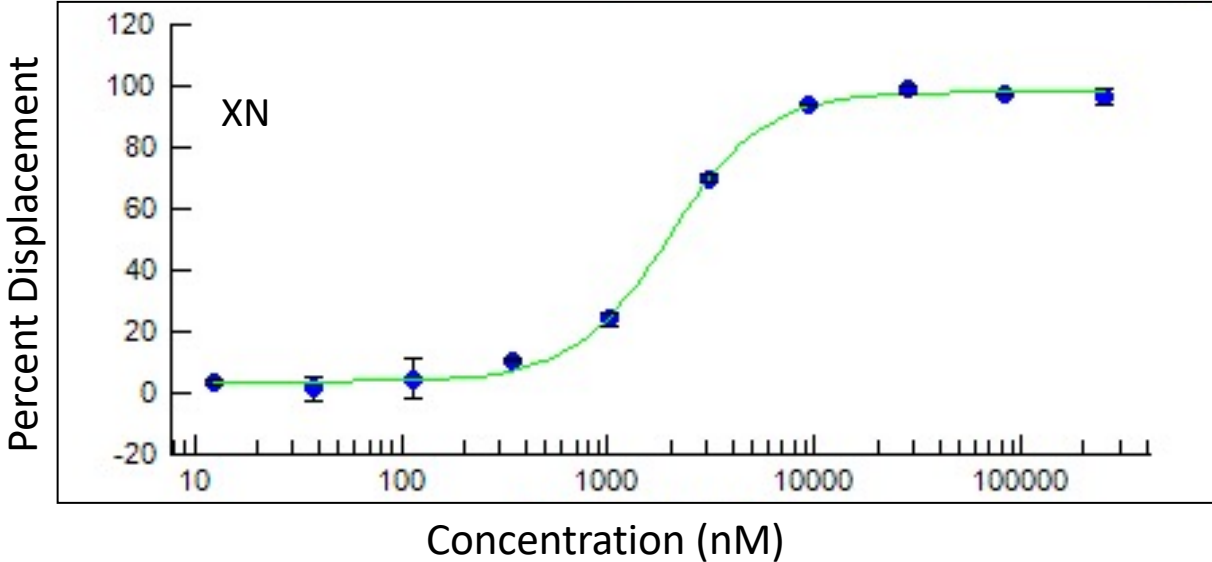

C

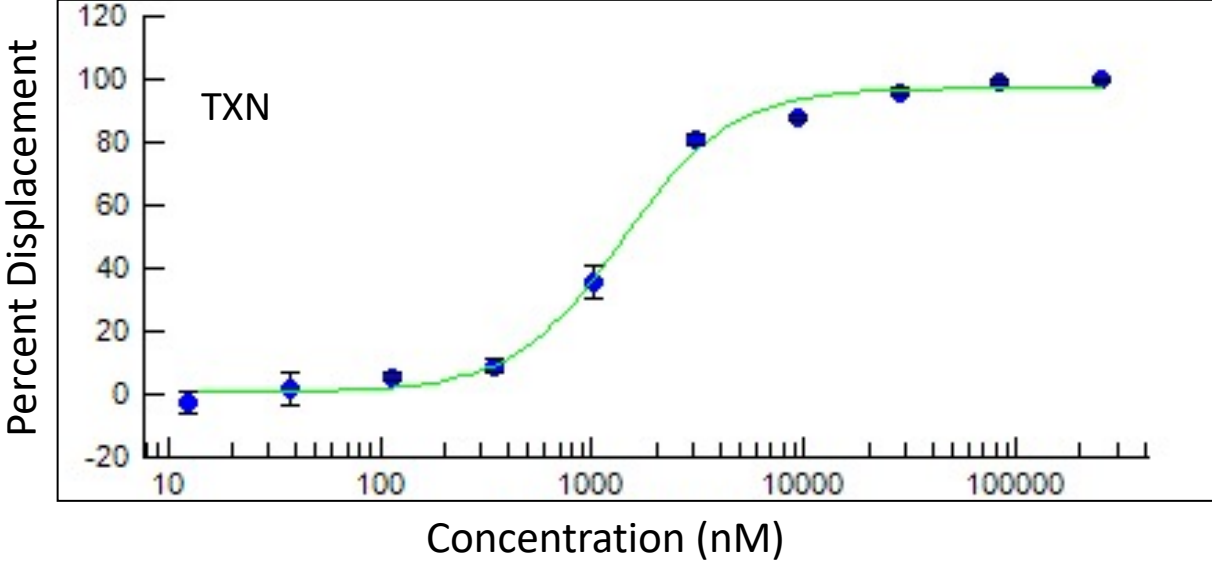

D

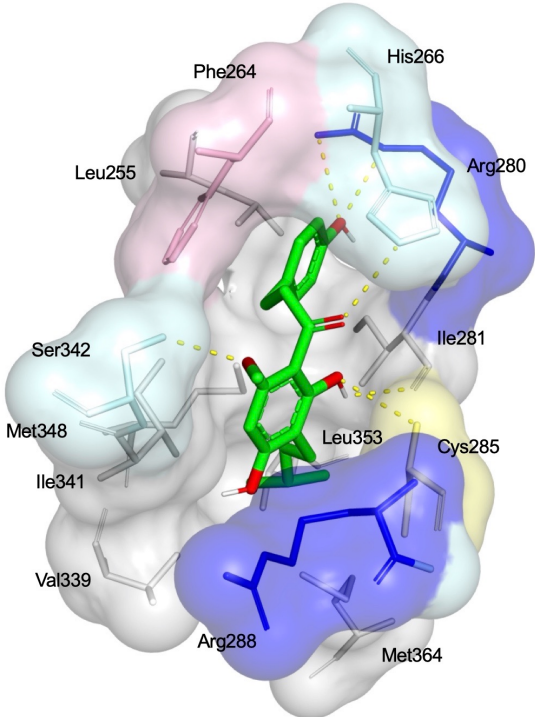

E

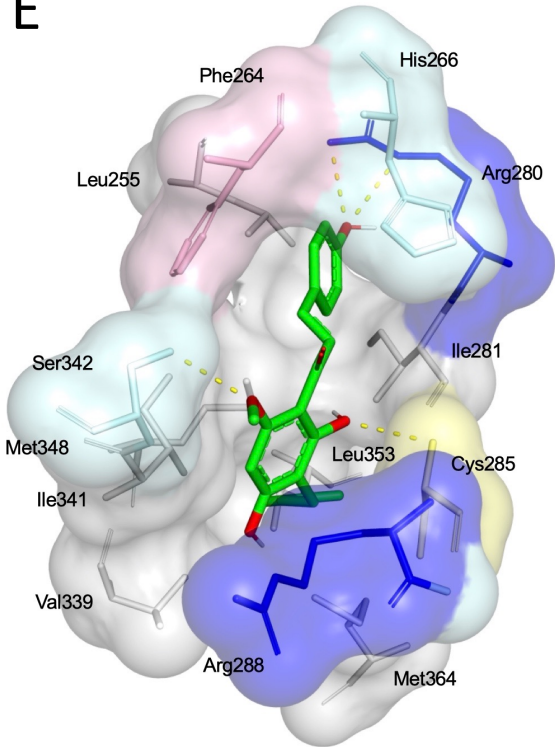

Supplement: Figure 13—source data 1. [file elife-66398-fig13-data1.zip › Figure13/figure13.pdf]
