# Supplementary material for: Tetrahydroxanthohumol, a xanthohumol derivative, attenuates high-fat diet-induced hepatic steatosis by antagonizing PPARγ
Source: eLife. 2021 Jun 15;10:e66398. doi: 10.7554/eLife.66398 (PMC8205491; doi:10.7554/eLife.66398)
Supplement: Figure 3—source data 1. — This zip archive contains the following: (1) One Comma Separated Values file named ‘metabolicGasExchange.csv’ contains metabolic cage gas exchange data. (2) One Comma Separated Values file named ‘fig3_table.csv’ contains phenotypic data directly pertaining to Figure 3. (3) One Comma Separated Values file named ‘fig3_stat_corrected.csv’ contains corrected metabolic cage gas exchange data directly pertaining to Figure 3. (4) A Jupyter Notebook file contains scripts used for statistical analysis and generation of Figure 3. (5) An R script file ‘ggplotRegression.R’. (6) A folder named ‘Figure 3—figure supplement 1’ containing Figure 3—figure supplement 1. (a) One Comma Separated Values file named ‘metabolicGasExchange.csv’ contains metabolic cage gas exchange data. (b) One Comma Separated Values file named ‘supplement1Table.csv’ contains phenotypic data directly pertaining to Figure 3—figure supplement 1. (c) An R script file “ggplotRegression.R. (d) A Jupyter Notebook file contains scripts used for statistical analysis and generation of Figure 3—figure supplement 1. (7) A folder named ‘Fig3Sup2’ containing Figure 3—figure supplement 2. (a) One Comma Separated Values file named ‘supplement2Table.csv’ contains phenotypic data directly pertaining to Figure 3—figure supplement 2. (b) An R script file ggplotRegression.R. (c) A Jupyter Notebook file contains scripts used for statistical analysis and generation of Figure 3—figure supplement 2. [file elife-66398-fig3-data1.zip › Figure3/Fig3Sup1/fig3Sup1.docx]

Figure supplement 1. Relationship of body mass and energy expenditure between (A) LFD and HFD; (B) LXN and HFD; (C) HXN and HFD; (D) TXN and HFD. Energy expenditure was measured between weeks 10 to 14. Data was analyzed using analysis of covariance (ANCOVA) of body mass upon entry into the cages and diet. No statistically significant effect from treatments was detected. HFD data are from the same group of mice and are displayed as a reference on all four panels. Source files of data used for the analysis are available in the Figure supplement 1—source data 1.
