# Supplementary material for: Tetrahydroxanthohumol, a xanthohumol derivative, attenuates high-fat diet-induced hepatic steatosis by antagonizing PPARγ
Source: eLife. 2021 Jun 15;10:e66398. doi: 10.7554/eLife.66398 (PMC8205491; doi:10.7554/eLife.66398)
Supplement: Figure 4—source data 1. — This zip archive contains the following: (1) One Comma Separated Values file named ‘fig4_table.csv’ phenotypic data directly pertaining to Figure 4. (2) An R script file ‘ggplotRegression.R’. (3) A Jupyter Notebook file contains scripts used for statistical analysis and generation of Figure 4. [file elife-66398-fig4-data1.zip › Figure4/Figure4.pdf]

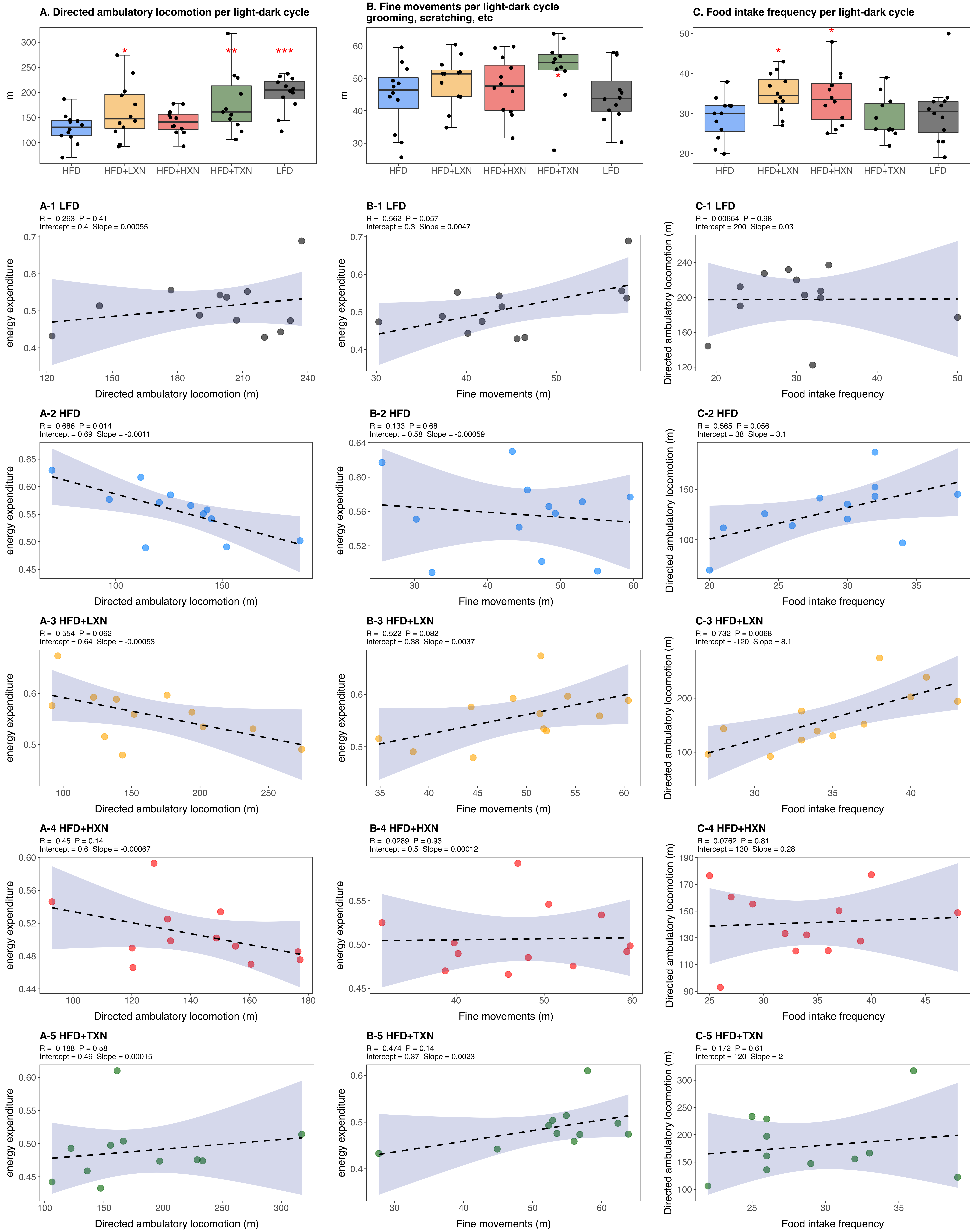
A. Directed ambulatory locomotion per light-dark cycle
B. Fine movements per light-dark cycle
grooming, scratching, etc
C. Food intake frequency per light-dark cycle
m
HFD
HFD+LXN
HFD+HXN
HFD+TXN
LFD
*
**
***
A-1 LFD
R = 0.263 P = 0.41
Intercept = 0.4 Slope = 0.00055
B-1 LFD
R = 0.562 P = 0.057
Intercept = 0.3 Slope = 0.0047
C-1 LFD
R = 0.00664 P = 0.98
Intercept = 200 Slope = 0.03
A-2 HFD
R = 0.686 P = 0.014
Intercept = 0.69 Slope = -0.0011
B-2 HFD
R = 0.133 P = 0.68
Intercept = 0.58 Slope = -0.00059
C-2 HFD
R = 0.565 P = 0.056
Intercept = 38 Slope = 3.1
A-3 HFD+LXN
R = 0.554 P = 0.062
Intercept = 0.64 Slope = -0.00053
B-3 HFD+LXN
R = 0.522 P = 0.082
Intercept = 0.38 Slope = 0.0037
C-3 HFD+LXN
R = 0.732 P = 0.0068
Intercept = -120 Slope = 8.1
A-4 HFD+HXN
R = 0.45 P = 0.14
Intercept = 0.6 Slope = -0.00067
B-4 HFD+HXN
R = 0.0289 P = 0.93
Intercept = 0.5 Slope = 0.00012
C-4 HFD+HXN
R = 0.0762 P = 0.81
Intercept = 130 Slope = 0.28
A-5 HFD+TXN
R = 0.188 P = 0.58
Intercept = 0.46 Slope = 0.00015
B-5 HFD+TXN
R = 0.474 P = 0.14
Intercept = 0.37 Slope = 0.0023
C-5 HFD+TXN
R = 0.172 P = 0.61
Intercept = 120 Slope = 2
energy expenditure
Directed ambulatory locomotion (m)
Fine movements (m)
Food intake frequency
