# Supplementary material for: Tetrahydroxanthohumol, a xanthohumol derivative, attenuates high-fat diet-induced hepatic steatosis by antagonizing PPARγ
Source: eLife. 2021 Jun 15;10:e66398. doi: 10.7554/eLife.66398 (PMC8205491; doi:10.7554/eLife.66398)
Supplement: Figure 8—source data 1. — This zip archive contains the following: (1) A folder named ‘raw’, containing five Excel workbooks. (a) ‘DEGs_TXN_vs_HFD.xlsx’. (b) ‘DOWN-GO_Biological_Process_2018.xlsx’. (c) ‘UP-GO_Biological_Process_2018.xlsx’. (d) ‘DOWN-KEGG_2019_Mouse.xlsx’. (e) ‘UP-KEGG_2019_Mouse.xlsx’. (2) A folder named ‘processed’, containing two Comma Separated Values files: (a) ‘BPTerms.csv’ (b) ‘KEGGterms.csv’. (3) A Jupyter Notebook file contains scripts used for statistical analysis and generation of Figure 8. (4) A pdf file named ‘txnHFDGO.pdf’. [file elife-66398-fig8-data1.zip › Figure8/txnHFDGO.pdf]

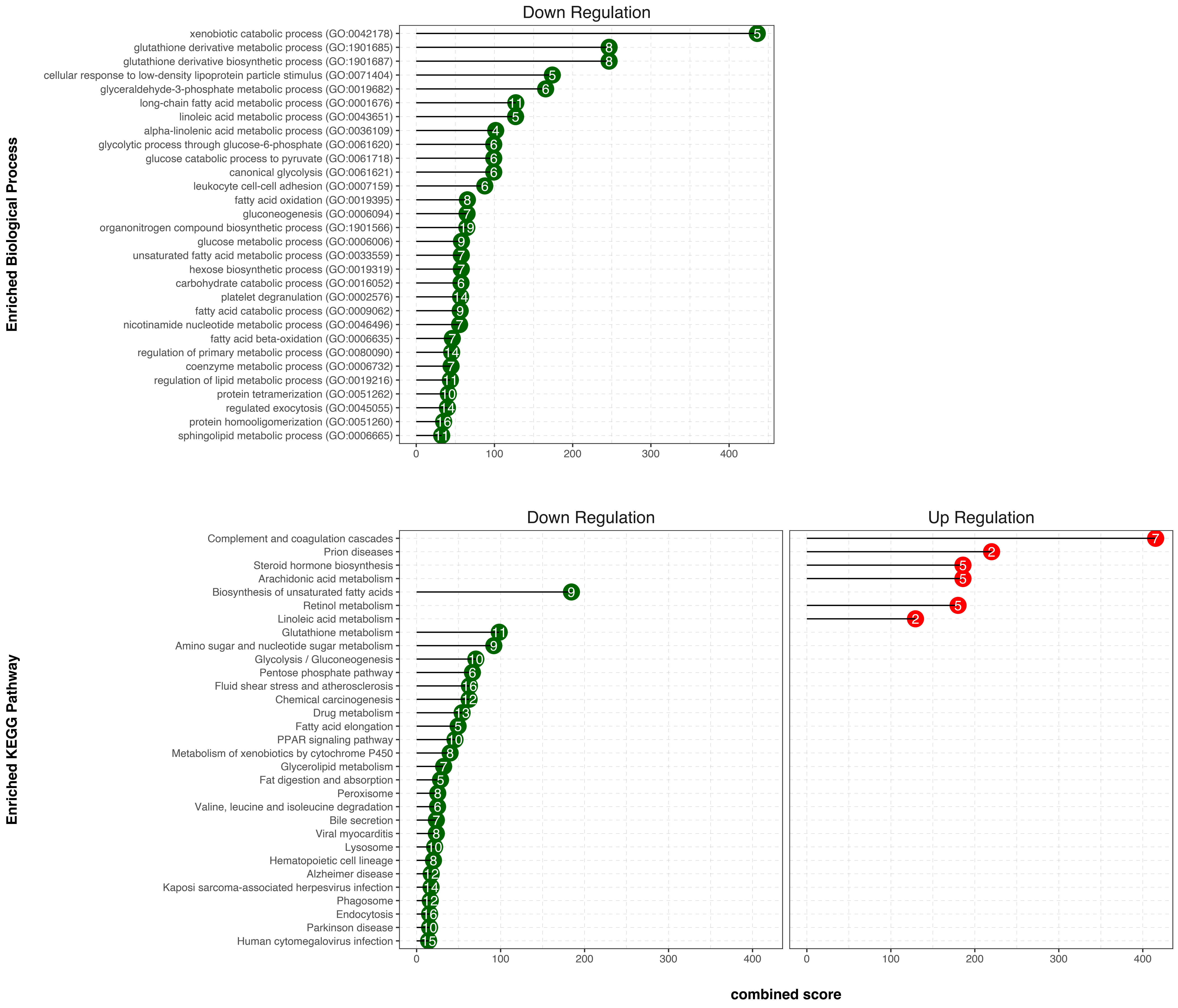
Down Regulation
Enriched Biological Process
xenobiotic catabolic process (GO:0042178)
glutathione derivative metabolic process (GO:1901685)
glutathione derivative biosynthetic process (GO:1901687)
cellular response to low-density lipoprotein particle stimulus (GO:0071404)
glyceraldehyde-3-phosphate metabolic process (GO:0019682)
long-chain fatty acid metabolic process (GO:0001676)
linoleic acid metabolic process (GO:0043651)
alpha-linolenic acid metabolic process (GO:0036109)
glycolytic process through glucose-6-phosphate (GO:0061620)
glucose catabolic process to pyruvate (GO:0061718)
canonical glycolysis (GO:0061621)
leukocyte cell-cell adhesion (GO:0007159)
fatty acid oxidation (GO:0019395)
gluconeogenesis (GO:0006094)
organonitrogen compound biosynthetic process (GO:1901566)
glucose metabolic process (GO:0006006)
unsaturated fatty acid metabolic process (GO:0033559)
hexose biosynthetic process (GO:0019319)
carbohydrate catabolic process (GO:0016052)
platelet degranulation (GO:0002576)
fatty acid catabolic process (GO:0009062)
nicotinamide nucleotide metabolic process (GO:0046496)
fatty acid beta-oxidation (GO:0006635)
regulation of primary metabolic process (GO:0080090)
coenzyme metabolic process (GO:0006732)
regulation of lipid metabolic process (GO:0019216)
protein tetramerization (GO:0051262)
regulated exocytosis (GO:0045055)
protein homooligomerization (GO:0051260)
sphingolipid metabolic process (GO:0006665)
5 8 8 5 6 11 5 4 6 6 6 6 8 7 19 9 7 7 6 14 9 7 7 14 7 11 10 14 16 11
0 100 200 300 400
Down Regulation
Up Regulation
Enriched KEGG Pathway
Complement and coagulation cascades
Prion diseases
Steroid hormone biosynthesis
Arachidonic acid metabolism
Biosynthesis of unsaturated fatty acids
Retinol metabolism
Linoleic acid metabolism
Glutathione metabolism
Amino sugar and nucleotide sugar metabolism
Glycolysis / Gluconeogenesis
Pentose phosphate pathway
Fluid shear stress and atherosclerosis
Chemical carcinogenesis
Drug metabolism
Fatty acid elongation
PPAR signaling pathway
Metabolism of xenobiotics by cytochrome P450
Glycerolipid metabolism
Fat digestion and absorption
Peroxisome
Valine, leucine and isoleucine degradation
Bile secretion
Viral myocarditis
Lysosome
Hematopoietic cell lineage
Alzheimer disease
Kaposi sarcoma-associated herpesvirus infection
Phagosome
Endocytosis
Parkinson disease
Human cytomegalovirus infection
9 11 9 10 6 16 12 13 5 10 8 7 5 8 6 7 8 10 8 12 14 12 16 10 15
7 2 5 5 5 2
0 100 200 300 400
0 100 200 300 400
combined score
